# Supplementary material for: Permanent pacemaker implantation after On-X surgical aortic valve replacement: SWEDEHEART observational study
Source: BMJ Open. 2021 Nov 17;11(11):e047962. doi: 10.1136/bmjopen-2020-047962 (PMC8603281; doi:10.1136/bmjopen-2020-047962)
Supplement: Supplementary data [file bmjopen-2020-047962supp001.pdf]

SUPPLEMENTAL MATERIAL

Permanent pacemaker implantation after On-X aortic valve replacement:

SWEDEHEART observational cohort study

Magnus Dalén, MD, PhD,[1,2] Michael Persson, MD,[1,2] Natalie Glaser, MD, PhD,[2,3]

Ulrik Sartipy, MD, PhD[1,2]

[1]Department of Cardiothoracic Surgery, Karolinska University Hospital, Stockholm, Sweden

[2]Department of Molecular Medicine and Surgery, Karolinska Institutet, Stockholm, Sweden

[3]Department of Cardiology, Stockholm South General Hospital, Stockholm, Sweden

Contents

Supplemental Table 1. Number of patients by year of surgery. 3
Supplemental Table 2. Number of patients according to hospital. 4
Supplemental Table 3. Number of pacemaker implants by year of surgery. 5
Supplemental Table 4. Number of pacemaker implants according to hospital. 6
Supplemental Table 5. Baseline characteristics after IPTW. 7
Supplemental Table 6. Baseline characteristics after overlap weighting. 10
Supplemental Table 7. Height, weight and body surface area. 13
Supplemental Figure 1. Proportion of On-X valves by year of surgery. 14
Supplemental Figure 2. Proportion of On-X valves according to hospital. 15
Supplemental Figure 3. Histogram of propensity scores before and after matching. 16
Supplemental Figure 4. Absolute standardized differences before (hollow circles) and after (filled circles) propensity score matching. 17
Supplemental Figure 5. Proportion of permanent pacemaker implants by year of surgery. 18
Supplemental Figure 6. Proportion of permanent pacemaker implants according to hospital. 19
Supplemental Figure 7. Absolute standardized differences before (hollow circles) and after (filled circles) inverse probability of treatment weighting. 20
Supplemental Figure 8. Absolute standardized differences before (hollow circles) and after (filled circles) overlap weighting. 21
Supplemental Figure 9. Kaplan-Meier estimated pacemaker implantation rate in the inverse probability of treatment weighted population. 22
Supplemental Figure 10. Kaplan-Meier estimated pacemaker implantation rate in the overlap weighted population. 23
Supplemental Figure 11. Crude and adjusted pacemaker implantation rate after On-X valve aortic valve replacement according to alternative approaches for confounding adjustment. 24

Supplemental Table 1. Number of patients by year of surgery.

Year of surgery	Total study population *n = 2602*	On-X valve No *n = 2021*	On-X valve Yes *n = 581*
2005	251 (100%)	244 (97%)	7 (2.8%)
2006	237 (100%)	230 (97%)	7 (3.0%)
2007	215 (100%)	187 (87%)	28 (13%)
2008	214 (100%)	176 (82%)	38 (18%)
2009	152 (100%)	110 (72%)	42 (28%)
2010	166 (100%)	120 (72%)	46 (28%)
2011	133 (100%)	100 (75%)	33 (25%)
2012	145 (100%)	110 (76%)	35 (24%)
2013	157 (100%)	120 (76%)	37 (24%)
2014	158 (100%)	112 (71%)	46 (29%)
2015	141 (100%)	100 (71%)	41 (29%)
2016	221 (100%)	148 (67%)	73 (33%)
2017	193 (100%)	121 (63%)	72 (37%)
2018	219 (100%)	143 (65%)	76 (35%)

Numbers are n (%).

Supplemental Table 2. Number of patients according to hospital.

Hospital	Total study population *n = 2602*	On-X valve	
		No *n = 2021*	Yes *n = 581*
1	467 (100%)	413 (88%)	54 (12%)
2	340 (100%)	227 (67%)	113 (33%)
3	329 (100%)	304 (92%)	25 (7.6%)
4	120 (100%)	120 (100%)	0 (0%)
5	398 (100%)	398 (100%)	0 (0%)
6	331 (100%)	243 (73%)	88 (27%)
7	221 (100%)	215 (97%)	6 (2.7%)
8	396 (100%)	101 (26%)	295 (74%)

Numbers are n (%).

Supplemental Table 3. Number of pacemaker implants by year of surgery.

Year of surgery	Total study population *n = 2602*	Pacemaker within 30 days: No *n = 2487*	Pacemaker within 30 days: Yes *n = 115*
2005	251 (100%)	243 (97%)	8 (3.2%)
2006	237 (100%)	226 (95%)	11 (4.6%)
2007	215 (100%)	209 (97%)	6 (2.8%)
2008	214 (100%)	207 (97%)	7 (3.3%)
2009	152 (100%)	144 (95%)	8 (5.3%)
2010	166 (100%)	157 (95%)	9 (5.4%)
2011	133 (100%)	130 (98%)	3 (2.3%)
2012	145 (100%)	139 (96%)	6 (4.1%)
2013	157 (100%)	151 (96%)	6 (3.8%)
2014	158 (100%)	153 (97%)	5 (3.2%)
2015	141 (100%)	135 (96%)	6 (4.3%)
2016	221 (100%)	207 (94%)	14 (6.3%)
2017	193 (100%)	182 (94%)	11 (5.7%)
2018	219 (100%)	204 (93%)	15 (6.8%)

Numbers are n (%).

Supplemental Table 4. Number of pacemaker implants according to hospital.

Hospital	Total study population *n = 2602*	Pacemaker within 30 days No *n = 2487*	Pacemaker within 30 days Yes *n = 115*
1	467 (100%)	442 (95%)	25 (5.4%)
2	340 (100%)	316 (93%)	24 (7.1%)
3	329 (100%)	311 (95%)	18 (5.5%)
4	120 (100%)	115 (96%)	5 (4.2%)
5	398 (100%)	389 (98%)	9 (2.3%)
6	331 (100%)	319 (96%)	12 (3.6%)
7	221 (100%)	213 (96%)	8 (3.6%)
8	396 (100%)	382 (96%)	14 (3.5%)

Numbers are n (%).

Supplemental Table 5. Baseline characteristics after IPTW.
Baseline characteristics in patients who underwent mechanical aortic valve replacement with an On-X valve or other mechanical valves in Sweden from 2005 to 2018 before and after inverse probability of treatment weighting.

	**Unweighted**			**IPTW***		
	Control	On-X	SMD	Control	On-X	SMD
Number of patients	2021	581		2573.7	2204.4	
Age (years), mean (SD)	54.1 (9.0)	51.0 (10.7)	0.322	53.5 (9.4)	52.8 (9.6)	0.067
Female sex	525 (26.0)	106 (18.2)	0.187	631.4 (24.5)	517.8 (23.5)	0.024
Non-Nordic birth region	207 (10.2)	32 (5.5)	0.176	238.7 (9.3)	148.1 (6.7)	0.094
Educational level			0.151			0.039
<10 years	588 (29.3)	132 (22.8)		716.6 (28.0)	579.0 (26.4)	
10-12 years	945 (47.1)	303 (52.2)		1230.2 (48.1)	1091.8 (49.8)	
>12 years	474 (23.6)	145 (25.0)		611.6 (23.9)	520.9 (23.8)	
Disposable household income (quartiles)			0.119			0.024
Q1 (low)	520 (25.7)	131 (22.5)		642.7 (25.0)	557.5 (25.3)	
Q2	506 (25.0)	145 (25.0)		648.0 (25.2)	547.9 (24.9)	
Q3	512 (25.3)	138 (23.8)		645.7 (25.1)	535.6 (24.3)	
Q4 (high)	483 (23.9)	167 (28.7)		637.3 (24.8)	563.5 (25.6)	
Married	1065 (52.7)	261 (44.9)	0.156	1318.3 (51.2)	1058.1 (48.0)	0.065
Body mass index (tertiles)			0.012			0.042
T1 (low)	623 (33.5)	185 (33.0)		804.0 (33.6)	743.2 (35.5)	

T2	618 (33.2)	189 (33.7)		798.0 (33.3)	685.6 (32.8)	
T3 (high)	620 (33.3)	187 (33.3)		791.3 (33.1)	663.3 (31.7)	
Atrial fibrillation	183 (9.1)	50 (8.6)	0.016	227.8 (8.9)	210.8 (9.6)	0.025
Heart failure	310 (15.3)	72 (12.4)	0.085	379.4 (14.7)	309.3 (14.0)	0.020
Left ventricular ejection fraction (%)			0.079			0.035
>50	1518 (75.1)	435 (74.9)		1935.0 (75.2)	1679.7 (76.2)	
30-50	389 (19.2)	122 (21.0)		499.8 (19.4)	422.0 (19.1)	
<30	114 (5.6)	24 (4.1)		138.8 (5.4)	102.7 (4.7)	
Chronic obstructive pulmonary disease	136 (6.7)	19 (3.3)	0.159	155.5 (6.0)	96.5 (4.4)	0.075
Diabetes	301 (14.9)	60 (10.3)	0.138	360.6 (14.0)	254.6 (11.5)	0.074
eGFR (ml/min/1,73 m^2)			0.154			0.050
>60	1865 (93.6)	554 (96.9)		2392.7 (94.3)	2066.5 (95.0)	
45-59	85 (4.3)	12 (2.1)		97.5 (3.8)	78.0 (3.6)	
30-44	24 (1.2)	4 (0.7)		27.1 (1.1)	22.7 (1.0)	
<30	18 (0.9)	2 (0.3)		20.3 (0.8)	9.2 (0.4)	
Preoperative dialysis	19 (1.0)	2 (0.4)	0.077	21.5 (0.9)	9.5 (0.5)	0.052
Prior myocardial infarction	193 (9.5)	42 (7.2)	0.084	235.7 (9.2)	173.2 (7.9)	0.047
Prior percutaneous coronary intervention	102 (5.0)	25 (4.3)	0.035	123.8 (4.8)	116.7 (5.3)	0.022
Peripheral vascular disease	208 (10.3)	97 (16.7)	0.188	297.4 (11.6)	294.6 (13.4)	0.055
Hypertension	703 (34.8)	217 (37.3)	0.053	905.7 (35.2)	789.8 (35.8)	0.013
Hyperlipidemia	344 (17.0)	89 (15.3)	0.046	431.7 (16.8)	317.2 (14.4)	0.066
Prior stroke	105 (5.2)	31 (5.3)	0.006	139.3 (5.4)	119.6 (5.4)	0.001

History of cancer	98 (4.8)	30 (5.2)	0.014	126.0 (4.9)	116.2 (5.3)	0.017
Alcohol dependence	69 (3.4)	15 (2.6)	0.049	84.6 (3.3)	50.8 (2.3)	0.059
Liver disease	18 (0.9)	3 (0.5)	0.045	20.9 (0.8)	9.5 (0.4)	0.049
Prior bleeding event	67 (3.3)	15 (2.6)	0.043	81.4 (3.2)	72.6 (3.3)	0.008
Prior endocarditis	92 (4.6)	26 (4.5)	0.004	118.3 (4.6)	116.2 (5.3)	0.031
Emergent operation	32 (1.6)	11 (1.9)	0.024	41.5 (1.6)	36.9 (1.7)	0.005
Coronary artery bypass grafting	363 (18.0)	79 (13.6)	0.120	438.9 (17.1)	296.2 (13.4)	0.101
Valve size (mm)			0.254			0.075
19-21	450 (22.7)	149 (25.8)		589.5 (23.2)	548.5 (25.3)	
23	704 (35.4)	256 (44.3)		944.9 (37.3)	841.0 (38.8)	
25-27	832 (41.9)	173 (29.9)		1001.4 (39.5)	780.5 (36.0)	

Numbers are n (%) unless otherwise specified.
eGFR = estimated glomerular filtration rate, SD = standard deviation, SMD = standardized mean difference
* The overall numbers of patients in each group are not necessarily integers owing to inverse probability of treatment weighting.

Supplemental Table 6. Baseline characteristics after overlap weighting.
Baseline characteristics in patients who underwent mechanical aortic valve replacement with an On-X valve or other mechanical valves in Sweden from 2005 to 2018 before and after overlap weighting.

	**Unweighted**			**Overlap weighting**		
	Control	On-X	SMD	Control	On-X	SMD
Number of patients	2021	581		407	407	
Age (years), mean (SD)	54.15 (9.01)	50.97 (10.70)	0.322	51.8 (10.3)	51.8 (10.2)	<0.001
Female sex	525 (26.0)	106 (18.2)	0.187	83 (20.4)	83 (20.4)	<0.001
Non-Nordic birth region	207 (10.2)	32 (5.5)	0.176	25 (6.2)	25 (6.2)	<0.001
Educational level			0.147			<0.001
<10 years	588 (29.1)	132 (22.7)		97 (23.8)	97 (23.8)	
10-12 years	959 (47.5)	304 (52.3)		210 (51.6)	210 (51.6)	
>12 years	474 (23.5)	145 (25.0)		100 (24.6)	100 (24.6)	
Disposable household income (quartiles)			0.119			<0.001
Q1 (low)	520 (25.7)	131 (22.5)		95 (23.4)	95 (23.4)	
Q2	506 (25.0)	145 (25.0)		102 (25.1)	102 (25.1)	
Q3	512 (25.3)	138 (23.8)		98 (24.0)	98 (24.0)	
Q4 (high)	483 (23.9)	167 (28.7)		112 (27.5)	112 (27.5)	
Married	1065 (52.7)	261 (44.9)	0.156	190 (46.7)	190 (46.7)	<0.001
Body mass index (tertiles)			0.053			<0.001
T1 (low)	623 (30.8)	185 (31.8)		131 (32.1)	131 (32.1)	

T2	778 (38.5)	209 (36.0)		149 (36.7)	149 (36.7)	
T3 (high)	620 (30.7)	187 (32.2)		127 (31.2)	127 (31.2)	
Atrial fibrillation	183 (9.1)	50 (8.6)	0.016	35 (8.5)	35 (8.5)	<0.001
Heart failure	310 (15.3)	72 (12.4)	0.085	52 (12.9)	52.4 (12.9)	<0.001
Left ventricular ejection fraction (%)			0.079			<0.001
>50	1518 (75.1)	435 (74.9)		307 (75.4)	307 (75.4)	
30-50	389 (19.2)	122 (21.0)		83 (20.3)	83 (20.3)	
<30	114 (5.6)	24 (4.1)		17 (4.3)	17 (4.3)	
Chronic obstructive pulmonary disease	136 (6.7)	19 (3.3)	0.159	16 (3.8)	16 (3.8)	<0.001
Diabetes	301 (14.9)	60 (10.3)	0.138	45 (11.0)	45 (11.0)	<0.001
eGFR (ml/min/1,73 m^2)			0.153			<0.001
>60	1894 (93.7)	563 (96.9)		392 (96.4)	392 (96.4)	
45-59	85 (4.2)	12 (2.1)		10 (2.4)	10 (2.4)	
30-44	24 (1.2)	4 (0.7)		3 (0.8)	3 (0.8)	
<30	18 (0.9)	2 (0.3)		2 (0.4)	2 (0.4)	
Preoperative dialysis	19 (0.9)	2 (0.3)	0.075	2 (0.4)	2 (0.4)	<0.001
Prior myocardial infarction	193 (9.5)	42 (7.2)	0.084	32 (7.9)	32 (7.9)	<0.001
Prior percutaneous coronary intervention	102 (5.0)	25 (4.3)	0.035	19 (4.6)	19 (4.6)	<0.001
Peripheral vascular disease	208 (10.3)	97 (16.7)	0.188	60 (14.7)	60 (14.7)	<0.001
Hypertension	703 (34.8)	217 (37.3)	0.053	149 (36.6)	149 (36.6)	<0.001
Hyperlipidemia	344 (17.0)	89 (15.3)	0.046	63 (15.6)	63 (15.6)	<0.001
Prior stroke	105 (5.2)	31 (5.3)	0.006	22 (5.4)	22 (5.4)	<0.001

History of cancer	98 (4.8)	30 (5.2)	0.014	22 (5.3)	22 (5.3)	<0.001
Alcohol dependence	69 (3.4)	15 (2.6)	0.049	11 (2.7)	11 (2.7)	<0.001
Liver disease	18 (0.9)	3 (0.5)	0.045	2 (0.6)	2 (0.6)	<0.001
Prior bleeding event	67 (3.3)	15 (2.6)	0.043	11 (2.7)	11 (2.7)	<0.001
Prior endocarditis	92 (4.6)	26 (4.5)	0.004	18 (4.4)	18 (4.4)	<0.001
Emergent operation	32 (1.6)	11 (1.9)	0.024	7 (1.7)	7 (1.7)	<0.001
Coronary artery bypass grafting	363 (18.0)	79 (13.6)	0.120	59 (14.5)	59 (14.5)	<0.001
Valve size (mm)			0.390			<0.001
19	74 (3.7)	32 (5.5)		21 (5.2)	21 (5.2)	
21	376 (18.6)	117 (20.1)		83 (20.4)	83 (20.4)	
23	739 (36.6)	259 (44.6)		176 (43.2)	176 (43.2)	
25	584 (28.9)	157 (27.0)		113 (27.7)	113 (27.7)	
27	248 (12.3)	16 (2.8)		14 (3.5)	14 (3.5)	

Numbers are n (%) unless otherwise specified.
eGFR = estimated glomerular filtration rate, SD = standard deviation, SMD = standardized mean difference

Supplemental Table 7. Height, weight and body surface area.

	Before matching			
	Overall	Control	On-X	SMD
Number of patients	2602	2021	581	
Height, cm	175 (9.4)	175 (9.5)	177 (8.8)	0.216
Weight, kg	86.0 (17.3)	85.5 (17.1)	87.5 (17.8)	0.116
BSA, m^2	2.04 (0.23)	2.03 (0.23)	2.07 (0.23)	0.154

Numbers are mean and standard deviation.
BSA = body surface area, SMD = standardized mean difference

Supplemental Figure 1. Proportion of On-X valves by year of surgery.

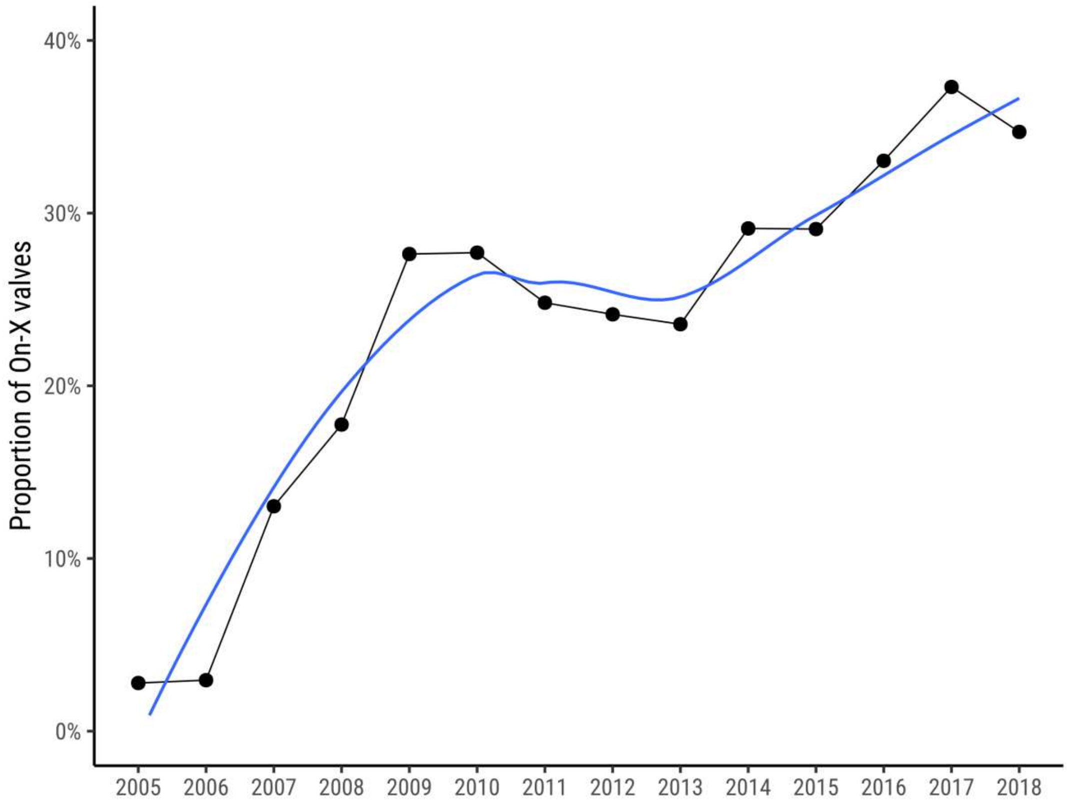

Supplemental Figure 2. Proportion of On-X valves according to hospital.

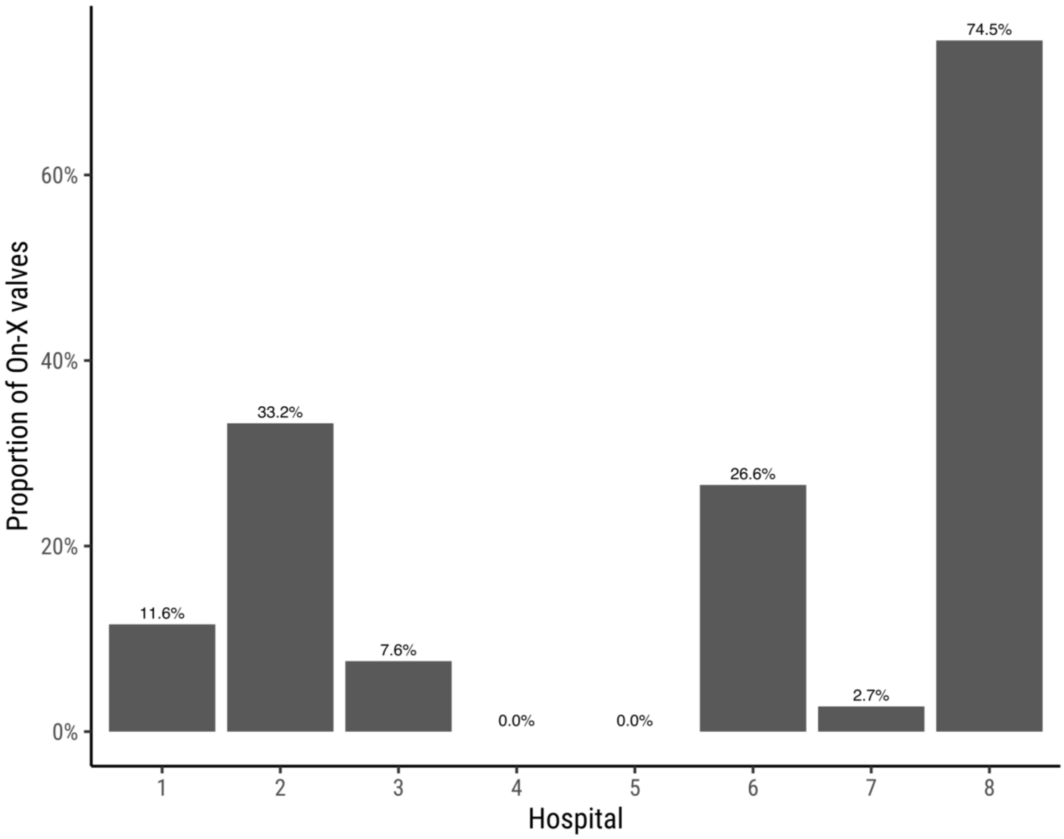

Supplemental Figure 3. Histogram of propensity scores before and after matching.

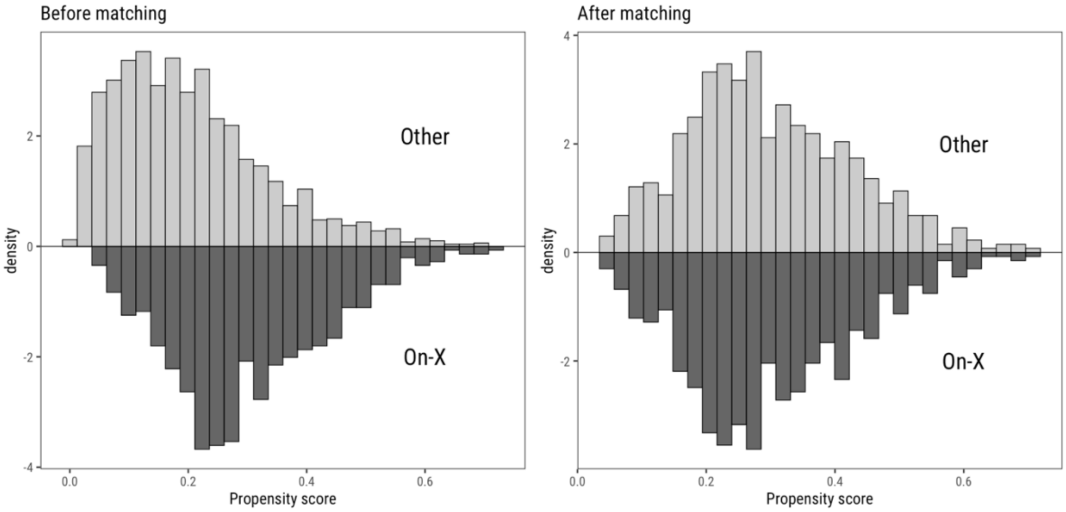

Supplemental Figure 4. Absolute standardized differences before (hollow circles) and after (filled circles) propensity score matching.

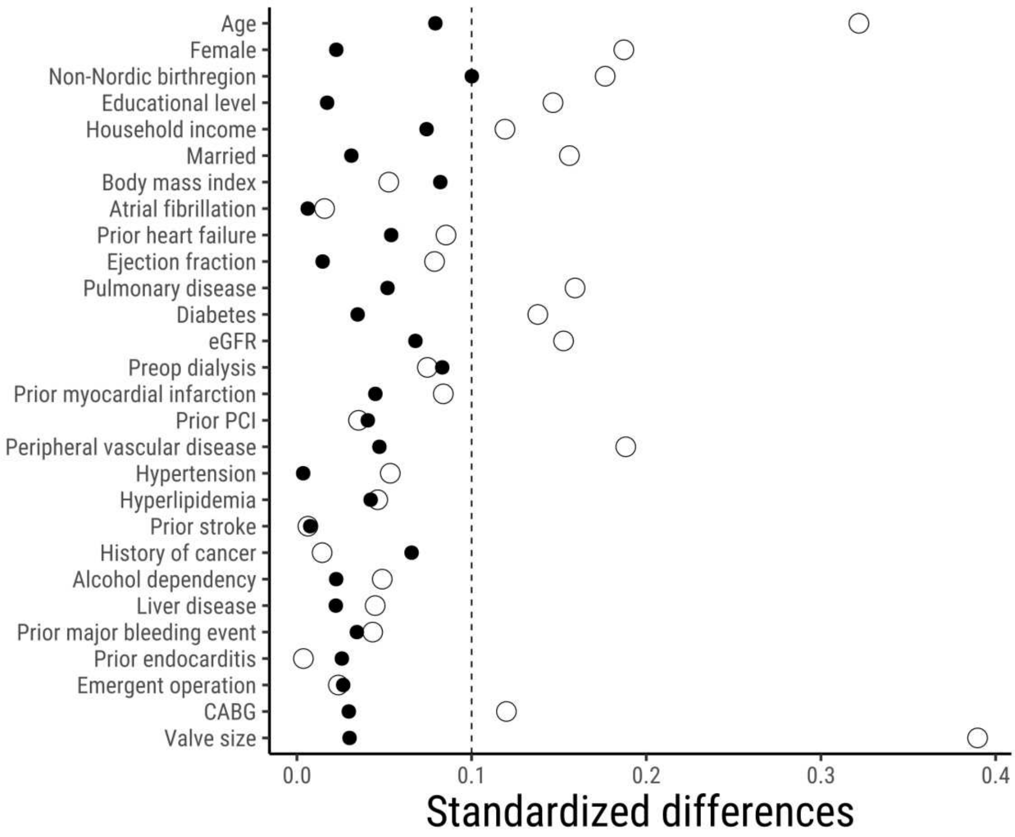

Supplemental Figure 5. Proportion of permanent pacemaker implants by year of surgery.

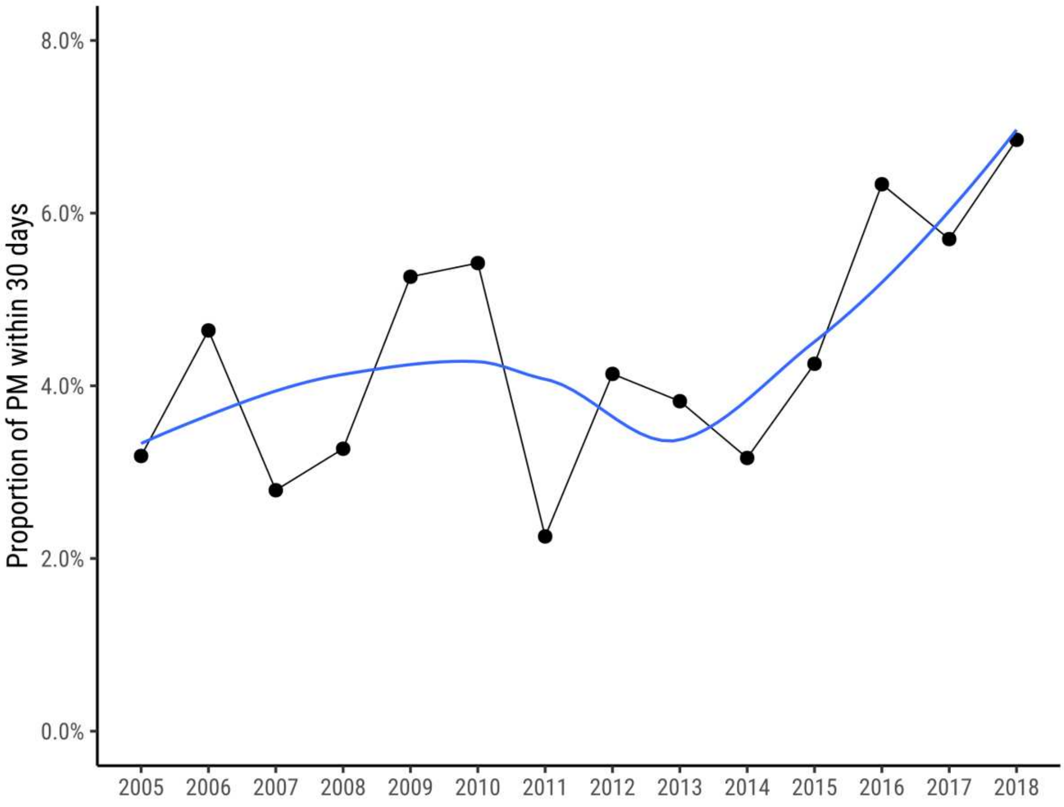

Supplemental Figure 6. Proportion of permanent pacemaker implants according to hospital.

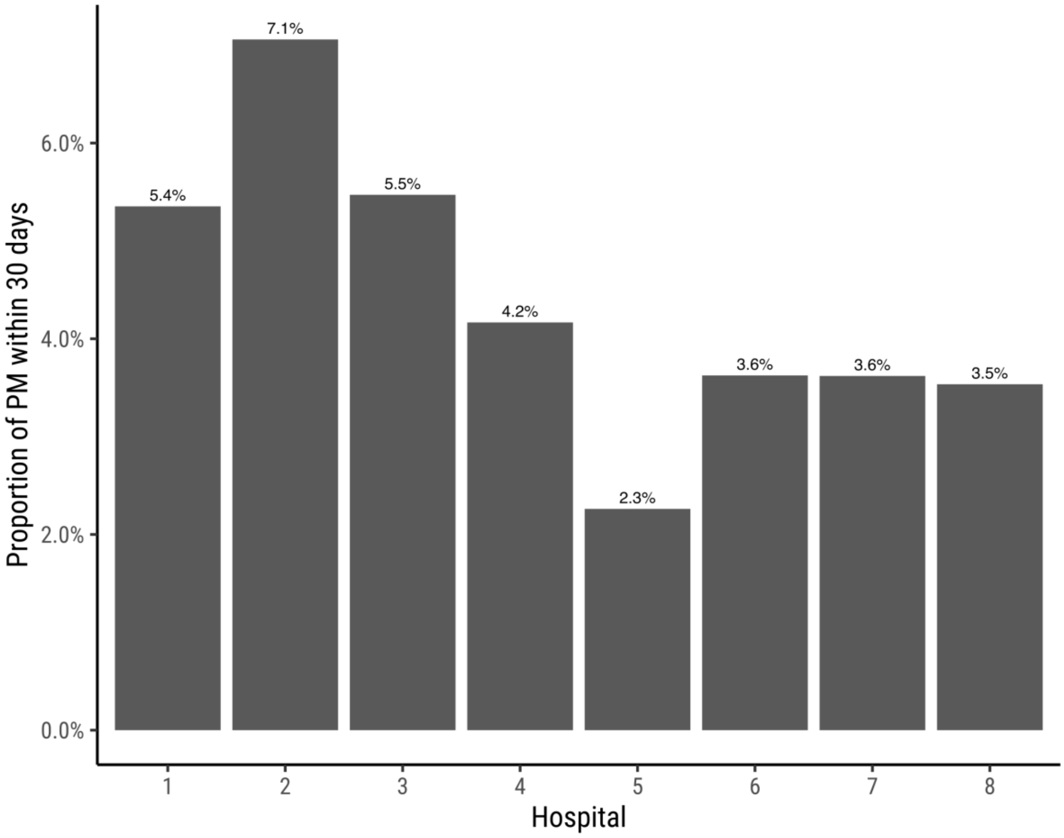

Supplemental Figure 7. Absolute standardized differences before (hollow circles) and after (filled circles) inverse probability of treatment weighting.

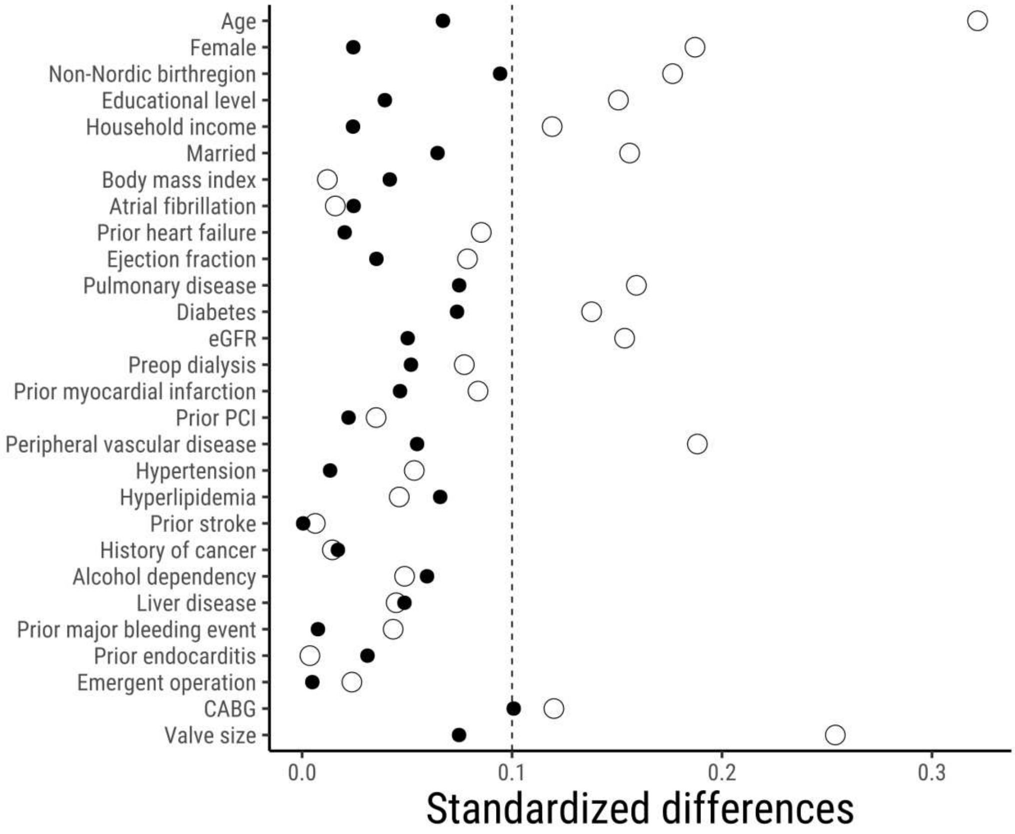

Supplemental Figure 8. Absolute standardized differences before (hollow circles) and after (filled circles) overlap weighting.
Note: This method yields exact covariate balance between treated and reference groups by construction.

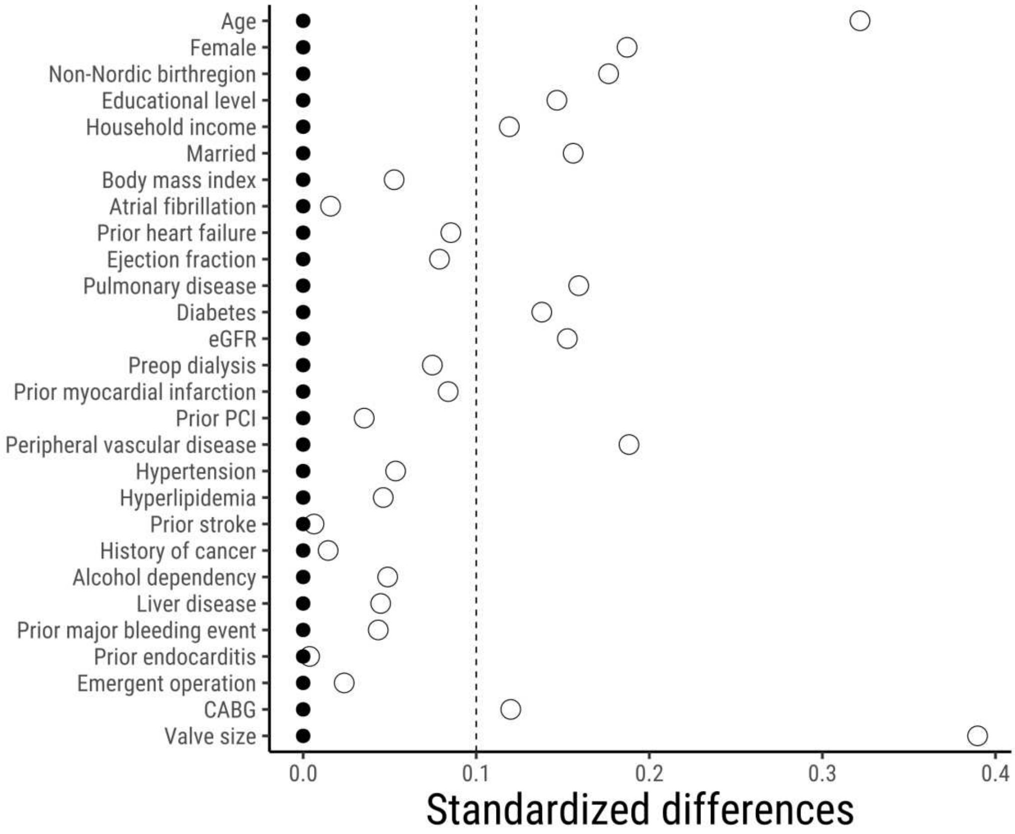

Supplemental Figure 9. Kaplan-Meier estimated pacemaker implantation rate in the inverse probability of treatment weighted population.

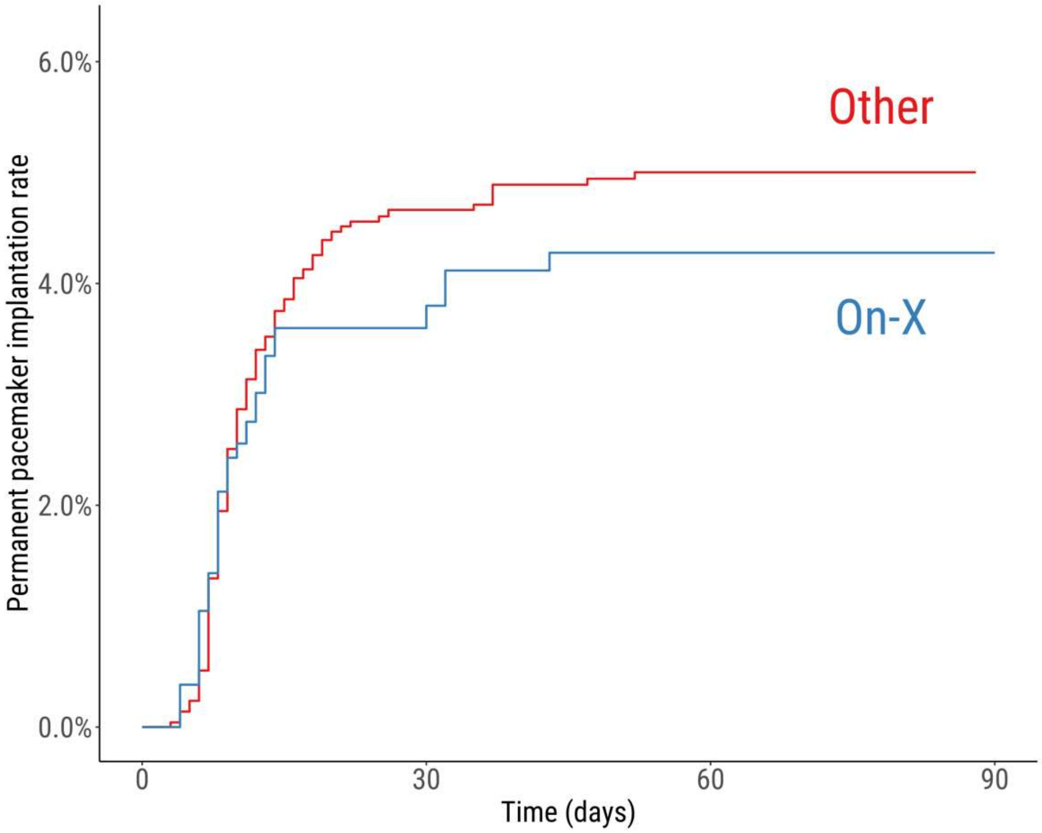

Supplemental Figure 10. Kaplan-Meier estimated pacemaker implantation rate in the overlap weighted population.

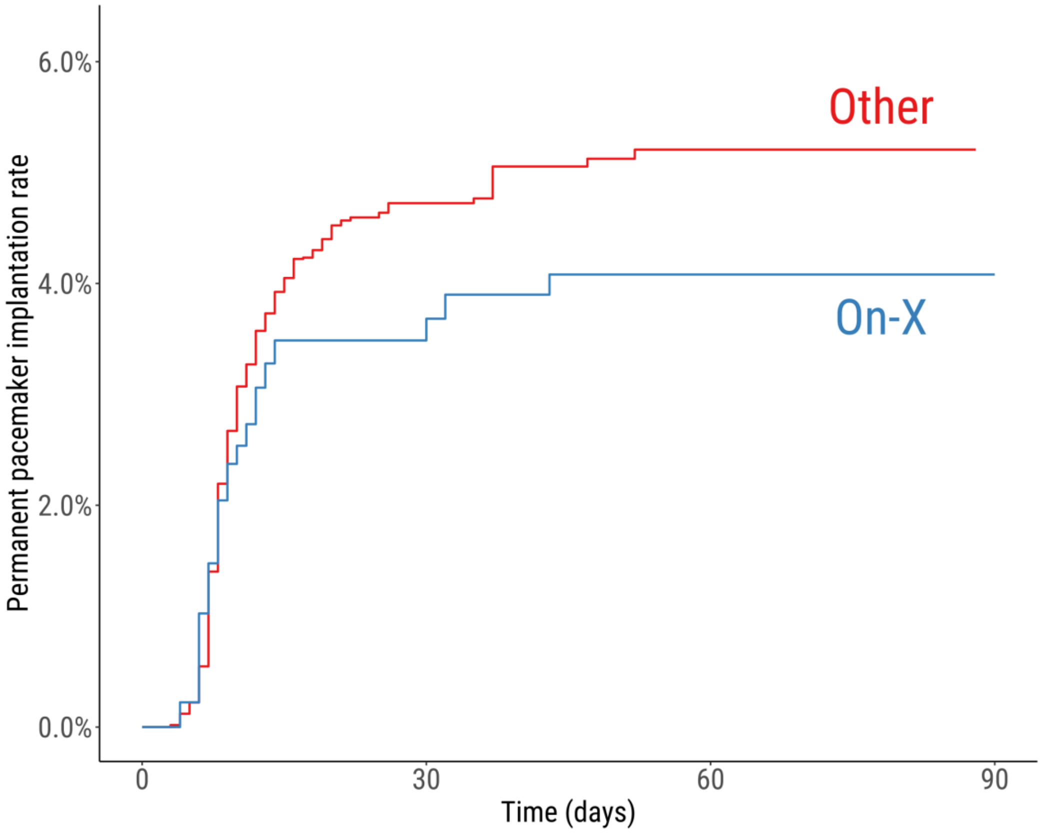

Supplemental Figure 11. Crude and adjusted pacemaker implantation rate after On-X valve aortic valve replacement according to alternative approaches for confounding adjustment.

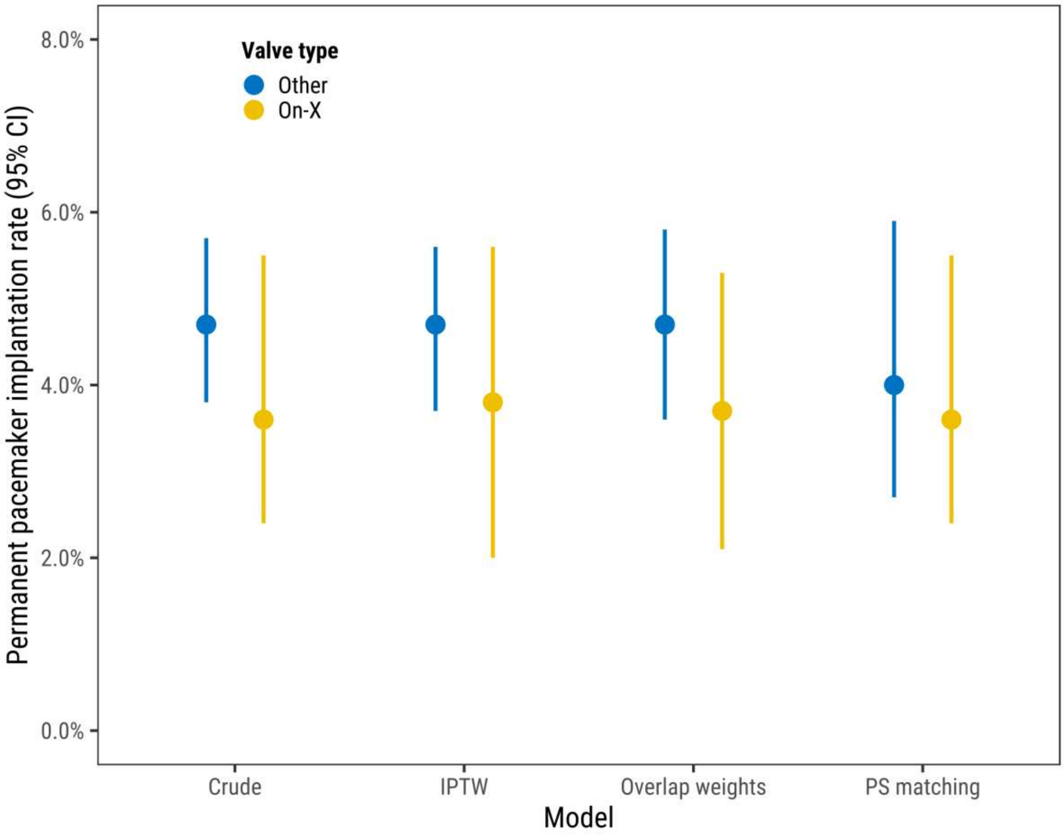

Note: IPTW = inverse probability of treatment weighting, PS = propensity score
