# Supplementary material for: Permanent pacemaker implantation after On-X surgical aortic valve replacement: SWEDEHEART observational study
Source: BMJ Open. 2021 Nov 17;11(11):e047962. doi: 10.1136/bmjopen-2020-047962 (PMC8603281; doi:10.1136/bmjopen-2020-047962)
Supplement: Author's manuscript [file bmjopen-2020-047962.draft_revisions.pdf]

BMJ Open

BMJ Open is committed to open peer review. As part of this commitment we make the peer review history of every article we publish publicly available.

When an article is published we post the peer reviewers' comments and the authors' responses online. We also post the versions of the paper that were used during peer review. These are the versions that the peer review comments apply to.

The versions of the paper that follow are the versions that were submitted during the peer review process. They are not the versions of record or the final published versions. They should not be cited or distributed as the published version of this manuscript.

BMJ Open is an open access journal and the full, final, typeset and author-corrected version of record of the manuscript is available on our site with no access controls, subscription charges or pay-per-view fees (http://bmjopen.bmj.com).

If you have any questions on BMJ Open's open peer review process please email info.bmjopen@bmj.com

Permanent pacemaker implantation after On-X aortic valve replacement: SWEDEHEART observational study

Journal:	*BMJ Open*
Manuscript ID	bmjopen-2020-047962
Article Type:	Original research
Date Submitted by the Author:	15-Dec-2020

Complete List of Authors:	Dalén, Magnus; Karolinska Hospital, Department of Cardiothoracic Surgery; Karolinska Institutet, Department of Molecular Medicine and Surgery<br>Persson, Michael; Karolinska University Hospital, Department of Cardiothoracic Surgery; Karolinska Institutet<br>Glaser, Natalie; Karolinska Institutet, Department of Molecular Medicine and Surgery<br>Sartipy, Ulrik; Karolinska Institutet, Department of Cardiothoracic Surgery and Anesthesiology

Keywords:	Valvular heart disease < CARDIOLOGY, Pacing & electrophysiology < CARDIOLOGY, Cardiac surgery < SURGERY

SCHOLARONE™
Manuscripts

I, the Submitting Author has the right to grant and does grant on behalf of all authors of the Work (as defined in the below author licence), an exclusive licence and/or a non-exclusive licence for contributions from authors who are: i) UK Crown employees; ii) where BMJ has agreed a CC-BY licence shall apply, and/or iii) in accordance with the terms applicable for US Federal Government officers or employees acting as part of their official duties; on a worldwide, perpetual, irrevocable, royalty-free basis to BMJ Publishing Group Ltd ("BMJ") its licensees and where the relevant Journal is co-owned by BMJ to the co-owners of the Journal, to publish the Work in this journal and any other BMJ products and to exploit all rights, as set out in our licence.

The Submitting Author accepts and understands that any supply made under these terms is made by BMJ to the Submitting Author unless you are acting as an employee on behalf of your employer or a postgraduate student of an affiliated institution which is paying any applicable article publishing charge ("APC") for Open Access articles. Where the Submitting Author wishes to make the Work available on an Open Access basis (and intends to pay the relevant APC), the terms of reuse of such Open Access shall be governed by a Creative Commons licence – details of these licences and which Creative Commons licence will apply to this Work are set out in our licence referred to above.

Other than as permitted in any relevant BMJ Author's Self Archiving Policies, I confirm this Work has not been accepted for publication elsewhere, is not being considered for publication elsewhere and does not duplicate material already published. I confirm all authors consent to publication of this Work and authorise the granting of this licence.

For peer review only

Permanent pacemaker implantation after On-X aortic valve replacement: SWEDEHEART observational study

Running head: Pacemaker and On-X aortic valve

Magnus Dalén, MD, PhD,[1,2] Michael Persson, MD,[1,2] Natalie Glaser, MD, PhD,[2,3] Ulrik Sartipy, MD, PhD[1,2]

[1]Department of Cardiothoracic Surgery, Karolinska University Hospital, Stockholm, Sweden

[2]Department of Molecular Medicine and Surgery, Karolinska Institutet, Stockholm, Sweden

[3]Department of Cardiology, Stockholm South General Hospital, Stockholm, Sweden

For peer review only

Disclosures: The authors declare no conflict of interest.

Word count: 4379

Corresponding author:

Magnus Dalén MD, PhD
Department of Cardiothoracic Surgery
Karolinska University Hospital,
SE-171 76 Stockholm, Sweden
Tel.: +46 8 517 708 37
Fax: +46 8 33 19 31
Email address: Magnus.Dalen@ki.se

Abstract

Objective

Bioprosthetic aortic valves with an extended subannular component, such as transcatheter valves, exert increased compression on the cardiac conduction system and increase the risk for permanent pacemaker implantation. It is unknown if the On-X prosthetic valve, which has an elongated subannular valve housing, increases the risk of permanent pacemaker implantation following aortic valve replacement.

Design

Observational nationwide cohort study.

Setting

Swedish population-based study

Participants

All patients aged 18-65 years who underwent primary mechanical aortic valve replacement in Sweden between 2005 and 2018. We used the SWEDEHEART register and other Swedish national health-data registers.

Exposure

Patients implanted with an On-X valve versus patients implanted with other bileaflet mechanical valves.

Primary and secondary outcome measures

Primary outcome measure was permanent pacemaker implantation within 30 days of surgery.

Results

A total of 2602 patients were included, and 581 patients received an On-X valve and 2021 patients received a St Jude Masters/Regent (n=945) or Carbomedics Reduced valve (n=1076). In the total study population, 115 (4.4%) permanent pacemaker implantations were performed within 30 days after aortic valve replacement. In the propensity score matched population, there was no significant difference in the rate of permanent pacemaker implantation in the On-X

For peer review only

group compared to the control group: 3.6% (95% CI: 2.4% to 5.5%) vs. 4.0% (95% CI: 2.7% to 5.9%), p=0.878.

Conclusions

The On-X prosthetic heart valve was associated with a similarly low risk for permanent pacemaker implantation after aortic valve replacement compared with other conventional bileaflet mechanical valves. The On-X elongated subannular valve housing does not interfere with the cardiac conduction system

Key words: Aortic valve replacement; mechanical prosthetic heart valve; pacemaker.

For peer review only

Article summary

Strengths and limitations of this study:

- Data were obtained from high-quality national Swedish health data registers.
- Long and complete follow-up in a nationwide cohort.
- One limitation of the study was that data did not include preoperative conduction disorders known to increase the risk for postoperative pacemaker requirement, such as bundle branch block.
- Another limitation was that we had no information about specific indications of postoperative pacemaker implantation.

For peer review only

Introduction

Surgical aortic valve replacement can induce cardiac conduction disorders that require permanent pacemaker implantation in 2-6% of the patients.[1-4] The close proximity of the atrioventricular node and the bundle of His to the aortic valve annulus and the left ventricular outflow tract makes the cardiac conduction system prone to injury during implantation of an aortic valve prosthesis.[3] Compared with conventional intra-/supraannulary prosthetic valves, bioprosthetic valves with an extended subannular component, such as transcatheter and rapid deployment valves, exerts increased compression on the conduction system.[5, 6] These prosthetic valves, as well as the subannular implantation depth, are associated with markedly increased risks for permanent pacemaker implantation.[5-8] The current standard for mechanical aortic valves used in contemporary clinical practice are bileaflet valves implanted intra- or supraannulary. The On-X prosthetic valve (On-X Life Technologies, Austin, Texas) is one of the most commonly used bileaflet mechanical valves today. Its' design differs from other contemporary mechanical valves,[9] and it is the only mechanical heart valve approved for reduced international normalized ratio target range.[10, 11] In order to reduce turbulence and enhance blood flow over the prosthetic valve, the On-X consists of an elongated valve housing with a flared inlet that protrudes subannulary into the left ventricular outflow tract.[9] It is unknown whether this design increases the risk for conduction disorders requiring permanent pacemaker implantation.

We performed a nationwide population-based cohort study to analyze whether aortic valve replacement with implantation of the On-X prosthetic heart valve, compared with other conventional bileaflet mechanical valves, was associated with an increased risk for permanent pacemaker implantation.

For peer review only

Methods

This nationwide population-based observational cohort study followed the Strengthening the Reporting of Observational Studies in Epidemiology (STROBE) and the REporting of studies Conducted using Observational Routinely collected health Data (RECORD) guidelines for observational studies using routinely collected data.[12, 13] The study was approved by the Swedish Ethical Review Authority and the need for informed consent was waived (Registration number: 2019-04131).

For peer review only

Study population

The Swedish Web system for Enhancement and Development of Evidence-based care in Heart disease Evaluated According to Recommended Therapies (SWEDEHEART) register[14, 15] was used to identify all adult patients aged 18-65 who underwent primary mechanical aortic valve replacement between 2005 and 2018. Patients who had a permanent pacemaker or implantable cardioverter-defibrillator prior to surgery or who underwent valve replacement because of infective endocarditis, or who had concomitant mitral valve surgery were excluded. Implantation of the On-X prosthetic valve was the exposure and the two most common valve types (Carbomedics Reduced [LivaNova, London, United Kingdom] and St Jude Masters/Regent [St Jude Medical, St Paul, Minnesota, USA]) served as the control group. Thus, patients who received other valve models were excluded.

As previously described, individual-level data linking to other nationwide health care registries was performed using the unique personal identity numbers assigned to all Swedish residents.[16, 17] The National Patient Register[18] was used to acquire information regarding relevant prior medical history, and the LISA (Longitudinal integration database for health insurance and labor market studies) database, managed by Statistics Sweden, was used to obtain information regarding socioeconomic variabels.[19]

Outcomes

The primary outcome measure was the implantation of a permanent pacemaker or implantable cardioverter-defibrillator within 30 days following aortic valve replacement as identified by the International Classification of Diseases-codes (FPE00, FPE10, FPE20, FPE26, FPF00, FPF10, FPF20, FPG10, FPG20, FPG30, and FPG33) from the Swedish National Patient Register.[18] We included implantable cardioverter defibrillator in the primary outcome measure in order to capture patients who had dual indications for both pacing and defibrillation.

Statistical methods

Baseline characteristics were described with frequencies and percentages for categorical variables and means and standard deviations for continuous variables. To minimize confounding due to measured baseline covariates, we estimated propensity scores (the probability of receiving an On-X valve based on the observed data) using a logistic regression model that included all variables reported in Table 1. A propensity score matched cohort was created by 1:1 nearest neighbor matching on the logit of the propensity score without replacement and a caliper width of 0.2 times the standard deviation of the logit of the propensity score.[20] In a separate analysis, we also estimated propensity scores using generalized boosted regression modeling[21, 22] and used the scores for inverse probability of treatment weighting. Finally, we applied the propensity scores from a logistic regression model to construct overlap weights, a weighting method that yields exact covariate balance between the exposed and reference groups by construction.[23] Balance between the groups was assessed by standardized mean differences. An absolute standardized difference ≤0.1 was considered an ideal balance.[24] In the matched and weighed populations, respectively, the percentage of pacemaker implants and 95% confidence interval was compared between the groups using chi-square tests. To illustrate the pacemaker implantation rate the first 90 days postoperatively, we constructed Kaplan-Meier curves. Although death could be considered a competing event, it occurred rarely during the first 90 days of follow-up, and was therefore not accounted for. The

For peer review only

statistical analyses were performed with R version 4.0.2 (R Foundation for Statistical Computing, Vienna, Austria) and the Matching[25] and twang[22] packages.

Missing data

Although data were complete for most variables, including exposure and outcome, there were some missing data. The variables with missing data were body mass index, estimated glomerular filtration rate, valve size, and educational level with 6.9%, 1.5%, 0.7%, and 0.6% missing data, respectively. For these variables, missing data was imputed with the most common category. Missing data was handled differently in the inverse probability of treatment weighted analysis because the weights were constructed to also balance rates of missingness in both groups.[21, 22]

Patient and Public Involvement

There was no patient or public involvement in the design or conduct of this study.

For peer review only

Results

A total of 2602 patients were included in the study, and 581 patients received an On-X valve and 2021 patients received a St Jude Masters/Regent (n=945) or Carbomedics Reduced valve (n=1076) at the eight hospitals performing cardiac surgery in Sweden during the study period. The proportion of On-X valve implantations increased during the study period but varied markedly by hospital as shown in Supplemental Figure 1-2 and Supplemental Table 1-2. The mean age in the total study population was 53.4 years, and 24% were women. Before propensity score matching, there were differences in baseline characteristics between the groups as shown in Table 1. There were more women and the mean age was higher in the control group. Peripheral vascular disease was more common in the On-X valve group, whereas pulmonary disease, diabetes, and concomitant coronary artery bypass grafting was more common in the control group. The 30-day all-cause mortality was 0.69% in the On-X valve group vs. 0.64% in the control group (p=0.905). After propensity score matching, the two groups were well balanced across all baseline characteristics and no standardized mean differences were greater than 10% (Table 1 and Supplemental Figure 3).

Permanent pacemaker implantation following mechanical aortic valve replacement

In the total study population, 115 (4.4%) permanent pacemaker implantations were performed within 30 days after aortic valve replacement. In the On-X group, the number of pacemakers implanted was 21 (3.6%) vs. 94 (4.6%) in the control group (p=0.284). Implantable cardioverter-defibrillators accounted for 5% of the implantations in both groups. The pacemaker implantation rate increased slightly during the study period but was fairly similar between hospitals (Supplemental Figure 4-5 and Supplemental Table 3-4).

The Kaplan-Meier estimated rate of permanent pacemaker implantation in the propensity score matched population is shown in Figure 1. There was no significant difference in the rate of permanent pacemaker implantation within 30 days after aortic valve replacement with the On-X valve compared to the control group: 3.6% (95% CI: 2.4% to 5.5%) vs. 4.0% (95% CI: 2.7% to

For peer review only

5.9%), p=0.878. The odds ratio (95% CI) for a permanent pacemaker implantation within 30 days after On-X aortic valve replacement was 0.91 (0.49-1.67). The majority of permanent pacemaker implantations occurred during the first 30 days. No pacemakers were implanted from postoperative day 60 to postoperative day 120.

Alternative approaches for confounding adjustment

Two alternative approaches for confounding adjustment using propensity scores for construction of weights were conducted: inverse probability of treatment weighting and overlap weighting. Both approaches resulted in excellent balance in baseline characteristics between the groups (Supplemental Table 5-6 and Supplemental Figure 6-7). The results were very similar to the results obtained in the propensity score matched analysis, and we found no significant difference in the rate of permanent pacemaker implantation within 30 days after aortic valve replacement with an On-X valve compared to the control group.

In the inverse probability of treatment weighted population, the rate of permanent pacemaker implantation within 30 days after aortic valve replacement was 3.8% (95% CI: 2.0% to 5.6%) with an On-X valve vs. 4.7% (95% CI: 3.7% to 5.6%) in the control group, p = 0.428. The odds ratio (95% CI) for a permanent pacemaker implantation within 30 days after On-X aortic valve replacement was 0.81 (0.48-1.37).

In the population where overlap weights were used, the rate of permanent pacemaker implantation within 30 days after aortic valve replacement was 3.7% (95% CI: 2.1% to 5.3%) with an On-X valve vs. 4.7% (95% CI: 3.6% to 5.8%) in the control group, p = 0.357. The odds ratio (95% CI) for a permanent pacemaker implantation within 30 days after On-X aortic valve replacement was 0.79 (0.47-1.31).

The Kaplan-Meier estimated rates of permanent pacemaker implantation in the inverse probability of treatment weighted population, and the population where overlap weights were used, respectively, are shown in Supplemental Figure 8-9.

The results from all approaches are summarized in Supplemental Figure 10.

For peer review only

Discussion

The On-X prosthetic heart valve has an elongated subannular valve housing with a flared inlet that protrudes subannulary into the left ventricular outflow tract. This design distinguishes the On-X prosthetic valve from other contemporary bileaflet mechanical aortic valves that are implanted intra-/supraannulary and that do not extend subannulary (Figure 2). In this nationwide cohort study, aortic valve replacement with implantation of the On-X prosthetic heart valve was not associated with an increased risk for permanent pacemaker implantation compared with other intra-/supraannulary bileaflet mechanical valves.

Cardiac conduction abnormalities requiring permanent pacemaker implantation is a well-known complication of aortic valve replacement.[1] The widespread use of transcatheter and rapid deployment prosthetic valves, which are associated with a significant risk of injury to the conduction system, has generated an increased interest in research concerning permanent pacemaker implantation after aortic valve replacement.[5-7] Although the use of mechanical prosthetic valves has decreased during later years, these valves are still the primary choice for younger patients, for whom mechanical valves have been shown to be superior to biological valves, in terms of mortality and reoperation rates.[17, 26] Permanent pacemaker requirement has in some studies been associated with adverse events and impaired long-term survival.[2] The potential risks associated with permanent pacemaker requirement are particularly relevant for younger patients since they have a longer life expectancy.[27]

The incidence of permanent pacemaker implantation in the current study (4.4%) is in line with previous studies of mechanical aortic valve replacement in younger adults, reporting rates of 2.5-5%.[28, 29] Few head-to-head comparisons of mechanical bileaflet prosthetic valves have been reported[30] and none of these have analyzed permanent pacemaker implantation rates between different valves. Generally, there are very few reports regarding rates of permanent pacemaker implantation after mechanical aortic valve replacement and most prior studies concerning mechanical prosthetic valves are completely lacking information on permanent pacemaker rates.

For peer review only

Compression on the subannunlar tissue, and thereby subsequent possible compression of the cardiac conduction system can induce conduction disturbances necessitating permanent pacemaker implantation. Because bioprosthetic valves with an extended subannular component, such as transcatheter and rapid deployment prosthetic valves, have been associated with a markedly increased risk for permanent pacemaker implantation,[5-7] we aimed to analyze whether this would also be true for the On-X prosthetic valve. Despite its' elongated subannular valve housing, our results demonstrated that the On-X prosthetic heart valve did not confer a higher risk of permanent pacemaker implantation compared with other conventional intra-/supraannulary bileaflet mechanical valves. This might be explained by the differences in design and annular fixation between the On-X prosthetic valve and transcatheter/rapid deployment prosthetic valves. These valves, unlike the On-X, are balloon- or self-expandable bioprosthetic valves that rely on radial forces for deployment of a subannular frame for stabilization in the aortic annulus and left ventricular outflow tract. These valves are therefore oversized in relation to the aortic annulus. This is in contrast with the On-X valve which is sutured to the aortic annulus with no expansion of the subannular valve component. Correct implantation requires that the On-X subannular valve housing fit into the left ventricular outflow tract and oversizing is thereby not possible.

Limitations

This analysis has limitations. First, the data did not include preoperative conduction disorders known to increase the risk for postoperative pacemaker requirement, such as bundle branch block. We were therefore not able to adjust for preoperative conduction abnormalities. Second, we did not have information about specific indications of postoperative pacemaker implantation. Third, we included implantable cardiac defibrillators in the primary outcome measure, in order to capture patients who had dual indications for both pacing and defibrillation. It is possible that some of these patients only had indication for defibrillation.

For peer review only

Conclusions

The On-X prosthetic heart valve was associated with a similarly low risk for permanent pacemaker implantation after aortic valve replacement compared with other conventional bileaflet mechanical valves. This indicates that the On-X elongated subannular valve housing does not seem to inflict on the cardiac conduction system.

For peer review only

Data availability statement

All data relevant to the study are included in the article or uploaded as supplementary information.

Disclosures

The authors declare no conflict of interest.

Funding

This work was supported by a donation from Mr. Fredrik Lundberg (to MD, no grant number), the Swedish Heart-Lung Foundation (grant numbers 20180400 and 20190533 to US, and 20190570 to NG), Åke Wiberg Foundation (grant number M18-0016 to US), Region Stockholm (ALF project) (grant number 20180114 to US), the Swedish Society of Medicine (grant number SLS-934749 to NG), the Capio Research Foundation (grant number 2019-3239 to NG), the Eva and Oscar Ahrén Research Foundation (to NG), and the Seraphim Hospital Foundation (to NG).

Acknowledgments

We thank the SWEDEHEART steering committee for providing data for this study.

Author statement

All authors (MD, MP, NG, US) have made substantial contributions to the conception or design of the work; or the acquisition, analysis, or interpretation of data for the work; and drafting the work and revising it critically for important intellectual content; and made final approval of the version to be published; and agreed to be accountable for all aspects of the work in ensuring that questions related to the accuracy or integrity of any part of the work are appropriately investigated and resolved.

For peer review only

References

1. Moskowitz G, Hong KN, Giustino G, et al. Incidence and Risk Factors for Permanent Pacemaker Implantation Following Mitral or Aortic Valve Surgery. J Am Coll Cardiol. 2019;74:2607-2620
2. Mehaffey JH, Haywood NS, Hawkins RB, et al. Need for Permanent Pacemaker After Surgical Aortic Valve Replacement Reduces Long-Term Survival. Ann Thorac Surg. 2018;106:460-465
3. Wiggins NB, Chong DT, Houghtaling PL, et al. Incidence, indications, risk factors, and survival of patients undergoing cardiac implantable electronic device implantation after open heart surgery. Europace. 2017;19:1335-1342
4. Levack MM, Kapadia SR, Soltesz EG, et al. Prevalence of and Risk Factors for Permanent Pacemaker Implantation After Aortic Valve Replacement. Ann Thorac Surg. 2019;108:700-707
5. Coti I, Schukro C, Drevinja F, et al. Conduction disturbances following surgical aortic valve replacement with a rapid-deployment bioprosthesis. J Thorac Cardiovasc Surg. 2020 DOI:10.1016/j.jtcvs.2020.01.083
6. van der Boon RM, Nuis RJ, Van Mieghem NM, et al. New conduction abnormalities after TAVI--frequency and causes. Nat Rev Cardiol. 2012;9:454-463
7. Bouhout I, Mazine A, Rivard L, et al. Conduction Disorders After Sutureless Aortic Valve Replacement. Ann Thorac Surg. 2017;103:1254-1260
8. Fraccaro C, Napodano M, Tarantini G. Conduction disorders in the setting of transcatheter aortic valve implantation: a clinical perspective. Catheter Cardiovasc Interv. 2013;81:1217-1223
9. Chaudhary R, Garg J, Krishnamoorthy P, et al. On-X Valve: The Next Generation Aortic Valve. Cardiol Rev. 2017;25:77-83

For peer review only

10. Puskas J, Gerdisch M, Nichols D, et al. Reduced anticoagulation after mechanical aortic valve replacement: interim results from the prospective randomized on-X valve anticoagulation clinical trial randomized Food and Drug Administration investigational device exemption trial. J Thorac Cardiovasc Surg. 2014;147:1202-1210

11. Puskas JD, Gerdisch M, Nichols D, et al. Anticoagulation and Antiplatelet Strategies After On-X Mechanical Aortic Valve Replacement. J Am Coll Cardiol. 2018;71:2717-2726

12. Benchimol EI, Smeeth L, Guttmann A, et al. The REporting of studies Conducted using Observational Routinely-collected health Data (RECORD) statement. PLoS Med. 2015;12:e1001885

13. von Elm E, Altman DG, Egger M, et al. The Strengthening the Reporting of Observational Studies in Epidemiology (STROBE) statement: guidelines for reporting observational studies. J Clin Epidemiol. 2008;61:344-349

14. Jernberg T, Attebring MF, Hambraeus K, et al. The Swedish Web-system for Enhancement and Development of Evidence-based care in Heart disease Evaluated According to Recommended Therapies (SWEDEHEART). Heart. 2010;96:1617-1621

15. Vikholm P, Ivert T, Nilsson J, et al. Validity of the Swedish Cardiac Surgery Registry. Interact Cardiovasc Thorac Surg. 2018;27:67-74

16. Ludvigsson JF, Otterblad-Olausson P, Pettersson BU, Ekbom A. The Swedish personal identity number: possibilities and pitfalls in healthcare and medical research. Eur J Epidemiol. 2009;24:659-667

17. Glaser N, Jackson V, Holzmann MJ, Franco-Cereceda A, Sartipy U. Aortic valve replacement with mechanical vs. biological prostheses in patients aged 50-69 years. Eur Heart J. 2016;37:2658-2667

18. Ludvigsson JF, Andersson E, Ekbom A, et al. External review and validation of the Swedish national inpatient register. BMC Public Health. 2011;11:450

For peer review only

19. Ludvigsson JF, Svedberg P, Olen O, Bruze G, Neovius M. The longitudinal integrated database for health insurance and labour market studies (LISA) and its use in medical research. Eur J Epidemiol. 2019;34:423-437

20. Austin PC. Optimal caliper widths for propensity-score matching when estimating differences in means and differences in proportions in observational studies. Pharm Stat. 2011;10:150-161

21. Griffin BA, Ridgeway G, Morral A, et al. Toolkit for Weighting and Analysis of Nonequivalent Groups (TWANG) Website. http://www.rand.org/statistics/twang

22. Ridgeway G, McCaffrey D, Morral A, Griffin BA, Burgette L. twang: Toolkit for Weighting and Analysis of Nonequivalent Groups. https://CRAN.R-project.org/package=twang

23. Desai RJ, Franklin JM. Alternative approaches for confounding adjustment in observational studies using weighting based on the propensity score: a primer for practitioners. BMJ. 2019;367:l5657

24. Austin PC, Stuart EA. Moving towards best practice when using inverse probability of treatment weighting (IPTW) using the propensity score to estimate causal treatment effects in observational studies. Stat Med. 2015;34:3661-3679

25. Sekhon JS. Multivariate and Propensity Score Matching Software with Automated Balance Optimization: The Matching package for R. Journal of Statistical Software. 2011;42:1-52

26. Goldstone AB, Chiu P, Baiocchi M, et al. Mechanical or Biologic Prostheses for Aortic-Valve and Mitral-Valve Replacement. N Engl J Med. 2017;377:1847-1857

27. Glaser N, Persson M, Jackson V, Holzmann MJ, Franco-Cereceda A, Sartipy U. Loss in Life Expectancy After Surgical Aortic Valve Replacement: SWEDEHEART Study. J Am Coll Cardiol. 2019;74:26-33

28. Korteland NM, Etnel JRG, Arabkhani B, et al. Mechanical aortic valve replacement in non-elderly adults: meta-analysis and microsimulation. Eur Heart J. 2017;38:3370-3377

For peer review only

29. Bouhout I, Stevens LM, Mazine A, et al I. Long-term outcomes after elective isolated mechanical aortic valve replacement in young adults. J Thorac Cardiovasc Surg. 2014;148:1341-1346 e1341

30. Hatoum H, Maureira P, Dasi LP. A turbulence in vitro assessment of On-X and St Jude Medical prostheses. J Thorac Cardiovasc Surg. 2019. DOI: 10.1016/j.jtcvs.2019.02.046

For peer review only

Figure legends

Figure 1. The graph shows the Kaplan-Meier estimated permanent pacemaker implantation rate in propensity score matched patients who received an On-X valve compared to other valves. There was no significant difference in the pacemaker implant rate between the groups. The majority of permanent pacemaker implantations occurred during the first 30 days. No pacemakers were implanted from day 60 to day 120.

Figure 2. The On-X prosthetic heart valve (On-X Life Technologies, Austin, Texas, USA; left panel) has an elongated subannular valve housing with a flared inlet that protrudes subannulary into the left ventricular outflow tract, in close proximity to the cardiac conduction system. This design distinguishes the On-X prosthetic valve from other contemporary bileaflet mechanical aortic valves, such as the St Jude Regent (St Jude Medical, St Paul, Minnesota, USA; right panel), that are implanted intra-/supraannulary and do not extend subannulary. Illustration: Magnus Dalén.

For peer review only

Table 1. Baseline characteristics in patients who underwent aortic valve replacement in Sweden with an On-X valve or other mechanical valves before and after propensity score matching

	**Before matching**				**After propensity score matching**			
	Overall	Control	On-X	SMD	Overall	Control	On-X	SMD
Number of patients	2602	2021	581		1160	580	580	
Age (years), mean (SD)	53.4 (9.5)	54.1 (9.0)	51.0 (10.7)	0.322	51.4 (10.6)	51.9 (10.6)	51.0 (10.6)	0.079
Female sex	631 (24.3)	525 (26.0)	106 (18.2)	0.187	207 (17.8)	101 (17.4)	106 (18.3)	0.023
Non-Nordic birth region	239 (9.2)	207 (10.2)	32 (5.5)	0.176	52 (4.5)	20 (3.4)	32 (5.5)	0.100
Educational level				0.147				0.017
<10 years	720 (27.7)	588 (29.1)	132 (22.7)		266 (22.9)	135 (23.3)	131 (22.6)	
10-12 years	1263 (48.5)	959 (47.5)	304 (52.3)		604 (52.1)	300 (51.7)	304 (52.4)	
>12 years	619 (23.8)	474 (23.5)	145 (25.0)		290 (25.0)	145 (25.0)	145 (25.0)	
Disposable household income (quartiles)				0.119				0.074
Q1 (low)	651 (25.0)	520 (25.7)	131 (22.5)		260 (22.4)	129 (22.2)	131 (22.6)	
Q2	651 (25.0)	506 (25.0)	145 (25.0)		289 (24.9)	144 (24.8)	145 (25.0)	
Q3	650 (25.0)	512 (25.3)	138 (23.8)		262 (22.6)	124 (21.4)	138 (23.8)	
Q4 (high)	650 (25.0)	483 (23.9)	167 (28.7)		349 (30.1)	183 (31.6)	166 (28.6)	
Married	1326 (51.0)	1065 (52.7)	261 (44.9)	0.156	531 (45.8)	270 (46.6)	261 (45.0)	0.031
Body mass index (tertiles)				0.053				0.082
T1 (low)	808 (31.1)	623 (30.8)	185 (31.8)		387 (33.4)	203 (35.0)	184 (31.7)	
T2	987 (37.9)	778 (38.5)	209 (36.0)		418 (36.0)	209 (36.0)	209 (36.0)	

For peer review only

T3 (high)	807 (31.0)	620 (30.7)	187 (32.2)		355 (30.6)	168 (29.0)	187 (32.2)	
Atrial fibrillation	233 (9.0)	183 (9.1)	50 (8.6)	0.016	99 (8.5)	49 (8.4)	50 (8.6)	0.006
Heart failure	382 (14.7)	310 (15.3)	72 (12.4)	0.085	134 (11.6)	62 (10.7)	72 (12.4)	0.054
Left ventricular ejection fraction (%)				0.079				0.015
>50	1953 (75.1)	1518 (75.1)	435 (74.9)		872 (75.2)	437 (75.3)	435 (75.0)	
30-50	511 (19.6)	389 (19.2)	122 (21.0)		239 (20.6)	118 (20.3)	121 (20.9)	
<30	138 (5.3)	114 (5.6)	24 (4.1)		49 (4.2)	25 (4.3)	24 (4.1)	
Chronic obstructive pulmonary disease	155 (6.0)	136 (6.7)	19 (3.3)	0.159	33 (2.8)	14 (2.4)	19 (3.3)	0.052
Diabetes	361 (13.9)	301 (14.9)	60 (10.3)	0.138	114 (9.8)	54 (9.3)	60 (10.3)	0.035
eGFR (ml/min/1,73 m^2)				0.153				0.068
>60	2457 (94.4)	1894 (93.7)	563 (96.9)		1124 (96.9)	562 (96.9)	562 (96.9)	
45-59	97 (3.7)	85 (4.2)	12 (2.1)		22 (1.9)	10 (1.7)	12 (2.1)	
30-44	28 (1.1)	24 (1.2)	4 (0.7)		11 (0.9)	7 (1.2)	4 (0.7)	
<30	20 (0.8)	18 (0.9)	2 (0.3)		3 (0.3)	1 (0.2)	2 (0.3)	
Preoperative dialysis	21 (0.8)	19 (0.9)	2 (0.3)	0.075	2 (0.2)	0 (0.0)	2 (0.3)	0.083
Prior myocardial infarction	235 (9.0)	193 (9.5)	42 (7.2)	0.084	91 (7.8)	49 (8.4)	42 (7.2)	0.045
Prior percutaneous coronary intervention	127 (4.9)	102 (5.0)	25 (4.3)	0.035	55 (4.7)	30 (5.2)	25 (4.3)	0.041
Peripheral vascular disease	305 (11.7)	208 (10.3)	97 (16.7)	0.188	184 (15.9)	87 (15.0)	97 (16.7)	0.047
Hypertension	920 (35.4)	703 (34.8)	217 (37.3)	0.053	431 (37.2)	215 (37.1)	216 (37.2)	0.004
Hyperlipidemia	433 (16.6)	344 (17.0)	89 (15.3)	0.046	187 (16.1)	98 (16.9)	89 (15.3)	0.042
Prior stroke	136 (5.2)	105 (5.2)	31 (5.3)	0.006	63 (5.4)	32 (5.5)	31 (5.3)	0.008

For peer review only

History of cancer	128 (4.9)	98 (4.8)	30 (5.2)	0.014	69 (5.9)	39 (6.7)	30 (5.2)	0.066
Alcohol dependence	84 (3.2)	69 (3.4)	15 (2.6)	0.049	28 (2.4)	13 (2.2)	15 (2.6)	0.022
Liver disease	21 (0.8)	18 (0.9)	3 (0.5)	0.045	7 (0.6)	4 (0.7)	3 (0.5)	0.022
Prior bleeding event	82 (3.2)	67 (3.3)	15 (2.6)	0.043	27 (2.3)	12 (2.1)	15 (2.6)	0.034
Prior endocarditis	118 (4.5)	92 (4.6)	26 (4.5)	0.004	49 (4.2)	23 (4.0)	26 (4.5)	0.026
Emergent operation	43 (1.7)	32 (1.6)	11 (1.9)	0.024	20 (1.7)	9 (1.6)	11 (1.9)	0.026
Coronary artery bypass grafting	442 (17.0)	363 (18.0)	79 (13.6)	0.120	164 (14.1)	85 (14.7)	79 (13.6)	0.030
Valve size (mm)				0.390				0.030
19	106 (4.1)	74 (3.7)	32 (5.5)		63 (5.4)	31 (5.3)	32 (5.5)	
21	493 (18.9)	376 (18.6)	117 (20.1)		238 (20.5)	121 (20.9)	117 (20.2)	
23	998 (38.4)	739 (36.6)	259 (44.6)		520 (44.8)	262 (45.2)	258 (44.5)	
25	741 (28.5)	584 (28.9)	157 (27.0)		308 (26.6)	151 (26.0)	157 (27.1)	
27	264 (10.1)	248 (12.3)	16 (2.8)		31 (2.7)	15 (2.6)	16 (2.8)	

Numbers are n (%) unless otherwise specified.
eGFR = estimated glomerular filtration rate, SD = standard deviation, SMD = standardized mean difference

For peer review only

For peer review only

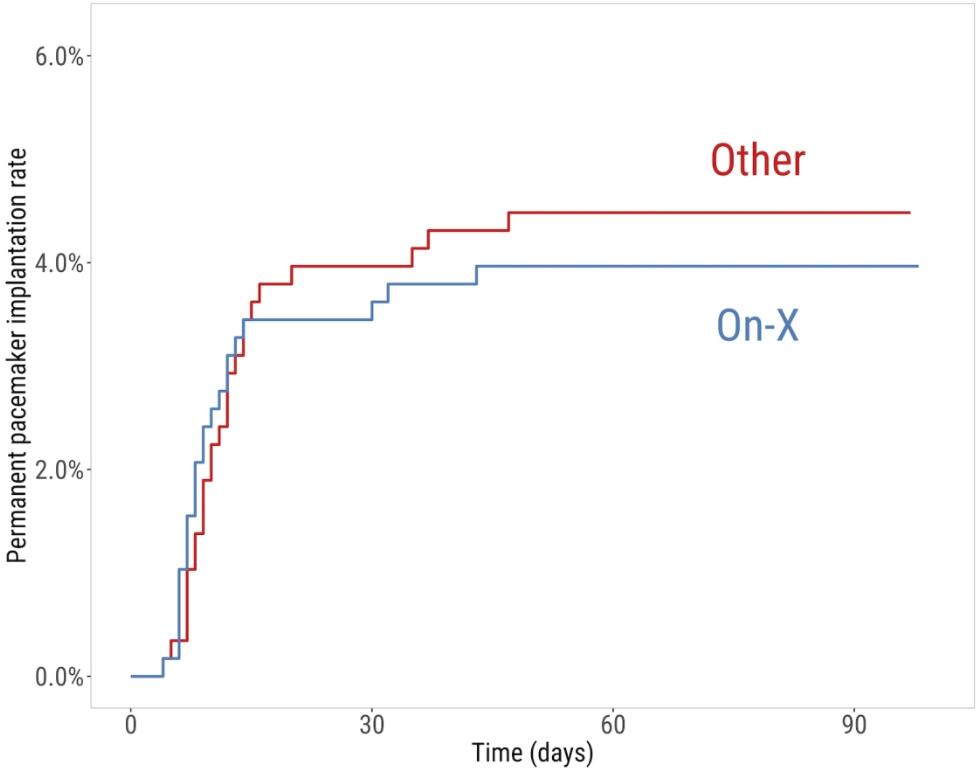

Figure 1. The graph shows the Kaplan-Meier estimated permanent pacemaker implantation rate in propensity score matched patients who received an On-X valve compared to other valves. There was no significant difference in the pacemaker implant rate between the groups. The majority of permanent pacemaker implantations occurred during the first 30 days. No pacemakers were implanted from day 60 to day 120.

150x117mm (300 x 300 DPI)

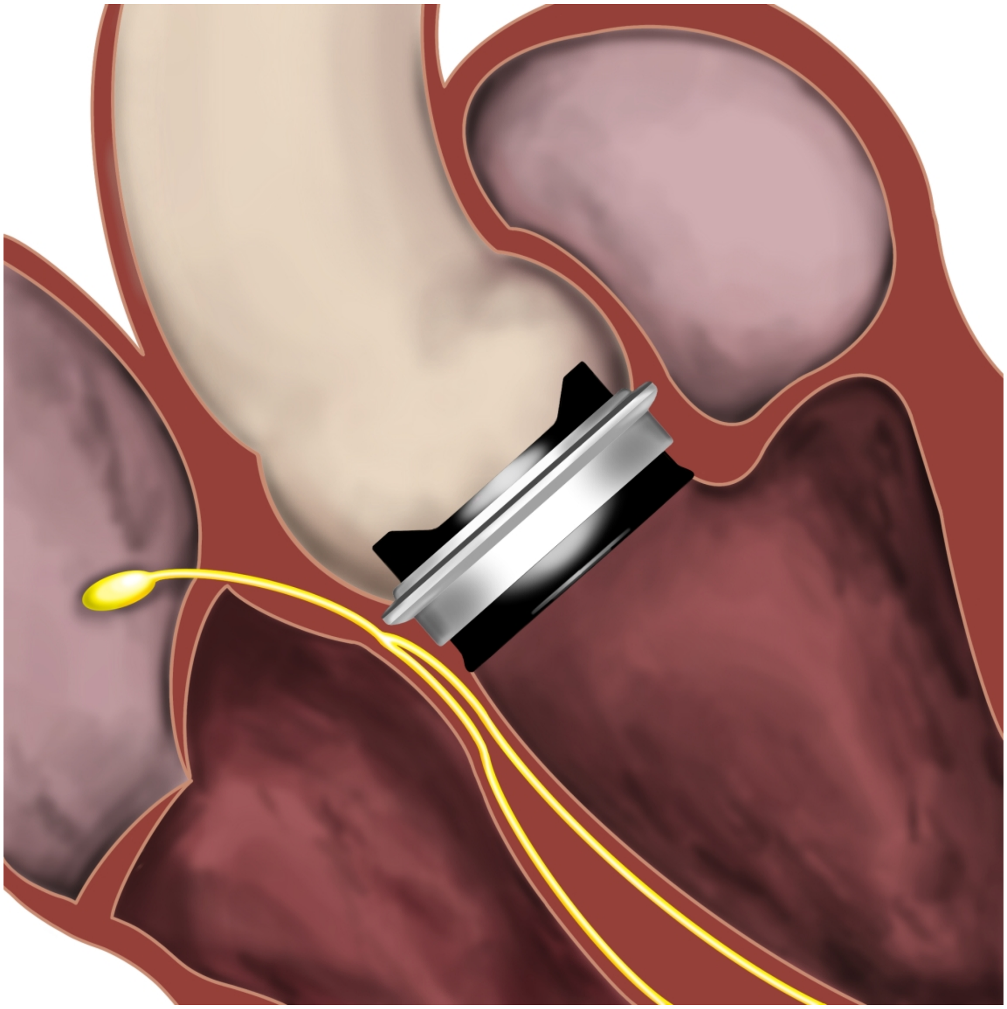

The On-X prosthetic heart valve (On-X Life Technologies, Austin, Texas, USA; left panel) has an elongated subannular valve housing with a flared inlet that protrudes subannulary into the left ventricular outflow tract, in close proximity to the cardiac conduction system. This design distinguishes the On-X prosthetic valve from other contemporary bileaflet mechanical aortic valves, such as the St Jude Regent (St Jude Medical, St Paul, Minnesota, USA; right panel), that are implanted intra-/supraannulary and do not extend subannulary. Illustration: Magnus Dalén.

394x394mm (132 x 132 DPI)

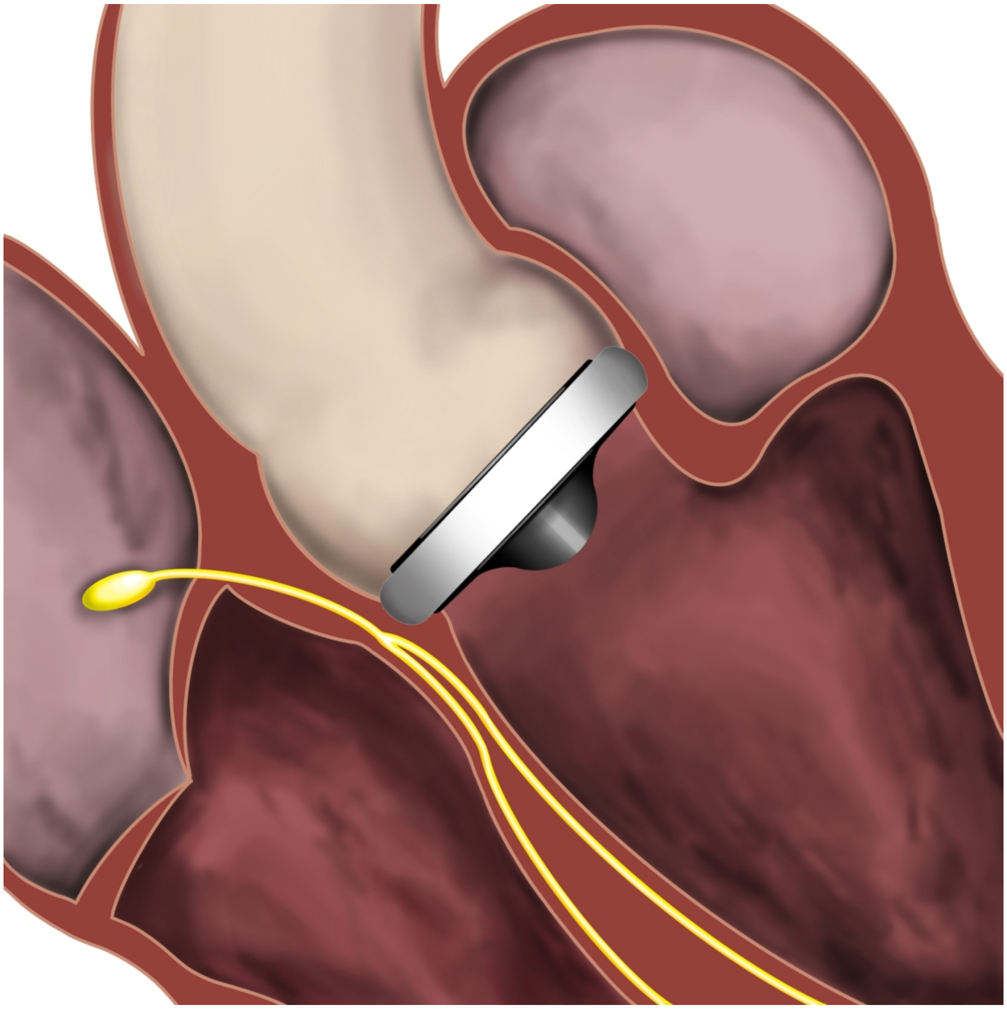

The On-X prosthetic heart valve (On-X Life Technologies, Austin, Texas, USA; left panel) has an elongated subannular valve housing with a flared inlet that protrudes subannulary into the left ventricular outflow tract, in close proximity to the cardiac conduction system. This design distinguishes the On-X prosthetic valve from other contemporary bileaflet mechanical aortic valves, such as the St Jude Regent (St Jude Medical, St Paul, Minnesota, USA; right panel), that are implanted intra-/supraannulary and do not extend subannulary. Illustration: Magnus Dalén.

394x394mm (132 x 132 DPI)

SUPPLEMENTAL MATERIAL

Permanent pacemaker implantation after On-X aortic valve replacement:

SWEDEHEART observational cohort study

Magnus Dalén, MD, PhD,[1,2] Michael Persson, MD,[1,2] Natalie Glaser, MD, PhD,[2,3]

Ulrik Sartipy, MD, PhD[1,2]

[1]Department of Cardiothoracic Surgery, Karolinska University Hospital, Stockholm, Sweden

[2]Department of Molecular Medicine and Surgery, Karolinska Institutet, Stockholm, Sweden

[3]Department of Cardiology, Stockholm South General Hospital, Stockholm, Sweden

For peer review only

Contents

Supplemental Table 1. Number of patients by year of surgery. 3
Supplemental Table 2. Number of patients according to hospital. 4
Supplemental Table 3. Number of pacemaker implants by year of surgery. 5
Supplemental Table 4. Number of pacemaker implants according to hospital. 6
Supplemental Table 5. Baseline characteristics after IPTW. 7
Supplemental Table 6. Baseline characteristics after overlap weighting. 10
Supplemental Figure 1. Proportion of On-X valves by year of surgery. 13
Supplemental Figure 1. Proportion of On-X valves according to hospital. 14
Supplemental Figure 3. Absolute standardized differences before (hollow circles) and after (filled circles) propensity score matching. 15
Supplemental Figure 4. Proportion of pacemaker implants by year of surgery. 16
Supplemental Figure 5. Proportion of pacemaker implants according to hospital. 17
Supplemental Figure 6. Absolute standardized differences before (hollow circles) and after (filled circles) inverse probability of treatment weighting. 18
Supplemental Figure 7. Absolute standardized differences before (hollow circles) and after (filled circles) overlap weighting. 19
Supplemental Figure 8. Kaplan-Meier estimated pacemaker implantation rate in the inverse probability of treatment weighted population. 20
Supplemental Figure 9. Kaplan-Meier estimated pacemaker implantation rate in the overlap weighted population. 21
Supplemental Figure 10. Crude and adjusted pacemaker implantation rate after On-X valve aortic valve replacement according to alternative approaches for confounding adjustment. 22

For peer review only

Supplemental Table 1. Number of patients by year of surgery.

		On-X valve	
Year of surgery	Total study population *n = 2602*	No *n = 2021*	Yes *n = 581*
2005	251 (100%)	244 (97%)	7 (2.8%)
2006	237 (100%)	230 (97%)	7 (3.0%)
2007	215 (100%)	187 (87%)	28 (13%)
2008	214 (100%)	176 (82%)	38 (18%)
2009	152 (100%)	110 (72%)	42 (28%)
2010	166 (100%)	120 (72%)	46 (28%)
2011	133 (100%)	100 (75%)	33 (25%)
2012	145 (100%)	110 (76%)	35 (24%)
2013	157 (100%)	120 (76%)	37 (24%)
2014	158 (100%)	112 (71%)	46 (29%)
2015	141 (100%)	100 (71%)	41 (29%)
2016	221 (100%)	148 (67%)	73 (33%)
2017	193 (100%)	121 (63%)	72 (37%)
2018	219 (100%)	143 (65%)	76 (35%)

Numbers are n (%).

For peer review only

Supplemental Table 2. Number of patients according to hospital.

Hospital	Total study population *n = 2602*	On-X valve: No *n = 2021*	On-X valve: Yes *n = 581*
1	467 (100%)	413 (88%)	54 (12%)
2	340 (100%)	227 (67%)	113 (33%)
3	329 (100%)	304 (92%)	25 (7.6%)
4	120 (100%)	120 (100%)	0 (0%)
5	398 (100%)	398 (100%)	0 (0%)
6	331 (100%)	243 (73%)	88 (27%)
7	221 (100%)	215 (97%)	6 (2.7%)
8	396 (100%)	101 (26%)	295 (74%)

Numbers are n (%).

For peer review only

Supplemental Table 3. Number of pacemaker implants by year of surgery.

Year of surgery	Total study population *n = 2602*	Pacemaker within 30 days: No *n = 2487*	Pacemaker within 30 days: Yes *n = 115*
2005	251 (100%)	243 (97%)	8 (3.2%)
2006	237 (100%)	226 (95%)	11 (4.6%)
2007	215 (100%)	209 (97%)	6 (2.8%)
2008	214 (100%)	207 (97%)	7 (3.3%)
2009	152 (100%)	144 (95%)	8 (5.3%)
2010	166 (100%)	157 (95%)	9 (5.4%)
2011	133 (100%)	130 (98%)	3 (2.3%)
2012	145 (100%)	139 (96%)	6 (4.1%)
2013	157 (100%)	151 (96%)	6 (3.8%)
2014	158 (100%)	153 (97%)	5 (3.2%)
2015	141 (100%)	135 (96%)	6 (4.3%)
2016	221 (100%)	207 (94%)	14 (6.3%)
2017	193 (100%)	182 (94%)	11 (5.7%)
2018	219 (100%)	204 (93%)	15 (6.8%)

Numbers are n (%).

For peer review only

Supplemental Table 4. Number of pacemaker implants according to hospital.

Hospital	Total study population *n = 2602*	Pacemaker within 30 days No *n = 2487*	Pacemaker within 30 days Yes *n = 115*
1	467 (100%)	442 (95%)	25 (5.4%)
2	340 (100%)	316 (93%)	24 (7.1%)
3	329 (100%)	311 (95%)	18 (5.5%)
4	120 (100%)	115 (96%)	5 (4.2%)
5	398 (100%)	389 (98%)	9 (2.3%)
6	331 (100%)	319 (96%)	12 (3.6%)
7	221 (100%)	213 (96%)	8 (3.6%)
8	396 (100%)	382 (96%)	14 (3.5%)

Numbers are n (%).

For peer review only

Supplemental Table 5. Baseline characteristics after IPTW.

Baseline characteristics in patients who underwent mechanical aortic valve replacement with an On-X valve or other mechanical valves in Sweden from 2005 to 2018 before and after inverse probability of treatment weighting.

	Unweighted			IPTW*		
	Control	On-X	SMD	Control	On-X	SMD
Number of patients	2021	581		2573.7	2204.4	
Age (years), mean (SD)	54.1 (9.0)	51.0 (10.7)	0.322	53.5 (9.4)	52.8 (9.6)	0.067
Female sex	525 (26.0)	106 (18.2)	0.187	631.4 (24.5)	517.8 (23.5)	0.024
Non-Nordic birth region	207 (10.2)	32 (5.5)	0.176	238.7 (9.3)	148.1 (6.7)	0.094
Educational level			0.151			0.039
<10 years	588 (29.3)	132 (22.8)		716.6 (28.0)	579.0 (26.4)	
10-12 years	945 (47.1)	303 (52.2)		1230.2 (48.1)	1091.8 (49.8)	
>12 years	474 (23.6)	145 (25.0)		611.6 (23.9)	520.9 (23.8)	
Disposable household income (quartiles)			0.119			0.024
Q1 (low)	520 (25.7)	131 (22.5)		642.7 (25.0)	557.5 (25.3)	
Q2	506 (25.0)	145 (25.0)		648.0 (25.2)	547.9 (24.9)	
Q3	512 (25.3)	138 (23.8)		645.7 (25.1)	535.6 (24.3)	
Q4 (high)	483 (23.9)	167 (28.7)		637.3 (24.8)	563.5 (25.6)	
Married	1065 (52.7)	261 (44.9)	0.156	1318.3 (51.2)	1058.1 (48.0)	0.065
Body mass index (tertiles)			0.012			0.042
T1 (low)	623 (33.5)	185 (33.0)		804.0 (33.6)	743.2 (35.5)	

For peer review only

T2	618 (33.2)	189 (33.7)		798.0 (33.3)	685.6 (32.8)	
T3 (high)	620 (33.3)	187 (33.3)		791.3 (33.1)	663.3 (31.7)	
Atrial fibrillation	183 (9.1)	50 (8.6)	0.016	227.8 (8.9)	210.8 (9.6)	0.025
Heart failure	310 (15.3)	72 (12.4)	0.085	379.4 (14.7)	309.3 (14.0)	0.020
Left ventricular ejection fraction (%)			0.079			0.035
>50	1518 (75.1)	435 (74.9)		1935.0 (75.2)	1679.7 (76.2)	
30-50	389 (19.2)	122 (21.0)		499.8 (19.4)	422.0 (19.1)	
<30	114 (5.6)	24 (4.1)		138.8 (5.4)	102.7 (4.7)	
Chronic obstructive pulmonary disease	136 (6.7)	19 (3.3)	0.159	155.5 (6.0)	96.5 (4.4)	0.075
Diabetes	301 (14.9)	60 (10.3)	0.138	360.6 (14.0)	254.6 (11.5)	0.074
eGFR (ml/min/1,73 m^2)			0.154			0.050
>60	1865 (93.6)	554 (96.9)		2392.7 (94.3)	2066.5 (95.0)	
45-59	85 (4.3)	12 (2.1)		97.5 (3.8)	78.0 (3.6)	
30-44	24 (1.2)	4 (0.7)		27.1 (1.1)	22.7 (1.0)	
<30	18 (0.9)	2 (0.3)		20.3 (0.8)	9.2 (0.4)	
Preoperative dialysis	19 (1.0)	2 (0.4)	0.077	21.5 (0.9)	9.5 (0.5)	0.052
Prior myocardial infarction	193 (9.5)	42 (7.2)	0.084	235.7 (9.2)	173.2 (7.9)	0.047
Prior percutaneous coronary intervention	102 (5.0)	25 (4.3)	0.035	123.8 (4.8)	116.7 (5.3)	0.022
Peripheral vascular disease	208 (10.3)	97 (16.7)	0.188	297.4 (11.6)	294.6 (13.4)	0.055
Hypertension	703 (34.8)	217 (37.3)	0.053	905.7 (35.2)	789.8 (35.8)	0.013
Hyperlipidemia	344 (17.0)	89 (15.3)	0.046	431.7 (16.8)	317.2 (14.4)	0.066
Prior stroke	105 (5.2)	31 (5.3)	0.006	139.3 (5.4)	119.6 (5.4)	0.001

For peer review only

History of cancer	98 (4.8)	30 (5.2)	0.014	126.0 (4.9)	116.2 (5.3)	0.017
Alcohol dependence	69 (3.4)	15 (2.6)	0.049	84.6 (3.3)	50.8 (2.3)	0.059
Liver disease	18 (0.9)	3 (0.5)	0.045	20.9 (0.8)	9.5 (0.4)	0.049
Prior bleeding event	67 (3.3)	15 (2.6)	0.043	81.4 (3.2)	72.6 (3.3)	0.008
Prior endocarditis	92 (4.6)	26 (4.5)	0.004	118.3 (4.6)	116.2 (5.3)	0.031
Emergent operation	32 (1.6)	11 (1.9)	0.024	41.5 (1.6)	36.9 (1.7)	0.005
Coronary artery bypass grafting	363 (18.0)	79 (13.6)	0.120	438.9 (17.1)	296.2 (13.4)	0.101
Valve size (mm)			0.254			0.075
19-21	450 (22.7)	149 (25.8)		589.5 (23.2)	548.5 (25.3)	
23	704 (35.4)	256 (44.3)		944.9 (37.3)	841.0 (38.8)	
25-27	832 (41.9)	173 (29.9)		1001.4 (39.5)	780.5 (36.0)	

Numbers are n (%) unless otherwise specified.
eGFR = estimated glomerular filtration rate, SD = standard deviation, SMD = standardized mean difference
* The overall numbers of patients in each group are not necessarily integers owing to inverse probability of treatment weighting.

For peer review only

Supplemental Table 6. Baseline characteristics after overlap weighting.
Baseline characteristics in patients who underwent mechanical aortic valve replacement with an On-X valve or other mechanical valves in Sweden from 2005 to 2018 before and after overlap weighting.

	Unweighted			Overlap weighting		
	Control	On-X	SMD	Control	On-X	SMD
Number of patients	2021	581		407	407	
Age (years), mean (SD)	54.15 (9.01)	50.97 (10.70)	0.322	51.8 (10.3)	51.8 (10.2)	<0.001
Female sex	525 (26.0)	106 (18.2)	0.187	83 (20.4)	83 (20.4)	<0.001
Non-Nordic birth region	207 (10.2)	32 (5.5)	0.176	25 (6.2)	25 (6.2)	<0.001
Educational level			0.147			<0.001
<10 years	588 (29.1)	132 (22.7)		97 (23.8)	97 (23.8)	
10-12 years	959 (47.5)	304 (52.3)		210 (51.6)	210 (51.6)	
>12 years	474 (23.5)	145 (25.0)		100 (24.6)	100 (24.6)	
Disposable household income (quartiles)			0.119			<0.001
Q1 (low)	520 (25.7)	131 (22.5)		95 (23.4)	95 (23.4)	
Q2	506 (25.0)	145 (25.0)		102 (25.1)	102 (25.1)	
Q3	512 (25.3)	138 (23.8)		98 (24.0)	98 (24.0)	
Q4 (high)	483 (23.9)	167 (28.7)		112 (27.5)	112 (27.5)	
Married	1065 (52.7)	261 (44.9)	0.156	190 (46.7)	190 (46.7)	<0.001
Body mass index (tertiles)			0.053			<0.001
T1 (low)	623 (30.8)	185 (31.8)		131 (32.1)	131 (32.1)	

For peer review only

T2	778 (38.5)	209 (36.0)		149 (36.7)	149 (36.7)	
T3 (high)	620 (30.7)	187 (32.2)		127 (31.2)	127 (31.2)	
Atrial fibrillation	183 (9.1)	50 (8.6)	0.016	35 (8.5)	35 (8.5)	<0.001
Heart failure	310 (15.3)	72 (12.4)	0.085	52 (12.9)	52.4 (12.9)	<0.001
Left ventricular ejection fraction (%)			0.079			<0.001
>50	1518 (75.1)	435 (74.9)		307 (75.4)	307 (75.4)	
30-50	389 (19.2)	122 (21.0)		83 (20.3)	83 (20.3)	
<30	114 (5.6)	24 (4.1)		17 (4.3)	17 (4.3)	
Chronic obstructive pulmonary disease	136 (6.7)	19 (3.3)	0.159	16 (3.8)	16 (3.8)	<0.001
Diabetes	301 (14.9)	60 (10.3)	0.138	45 (11.0)	45 (11.0)	<0.001
eGFR (ml/min/1,73 m^2)			0.153			<0.001
>60	1894 (93.7)	563 (96.9)		392 (96.4)	392 (96.4)	
45-59	85 (4.2)	12 (2.1)		10 (2.4)	10 (2.4)	
30-44	24 (1.2)	4 (0.7)		3 (0.8)	3 (0.8)	
<30	18 (0.9)	2 (0.3)		2 (0.4)	2 (0.4)	
Preoperative dialysis	19 (0.9)	2 (0.3)	0.075	2 (0.4)	2 (0.4)	<0.001
Prior myocardial infarction	193 (9.5)	42 (7.2)	0.084	32 (7.9)	32 (7.9)	<0.001
Prior percutaneous coronary intervention	102 (5.0)	25 (4.3)	0.035	19 (4.6)	19 (4.6)	<0.001
Peripheral vascular disease	208 (10.3)	97 (16.7)	0.188	60 (14.7)	60 (14.7)	<0.001
Hypertension	703 (34.8)	217 (37.3)	0.053	149 (36.6)	149 (36.6)	<0.001
Hyperlipidemia	344 (17.0)	89 (15.3)	0.046	63 (15.6)	63 (15.6)	<0.001
Prior stroke	105 (5.2)	31 (5.3)	0.006	22 (5.4)	22 (5.4)	<0.001

For peer review only

History of cancer	98 (4.8)	30 (5.2)	0.014	22 (5.3)	22 (5.3)	<0.001
Alcohol dependence	69 (3.4)	15 (2.6)	0.049	11 (2.7)	11 (2.7)	<0.001
Liver disease	18 (0.9)	3 (0.5)	0.045	2 (0.6)	2 (0.6)	<0.001
Prior bleeding event	67 (3.3)	15 (2.6)	0.043	11 (2.7)	11 (2.7)	<0.001
Prior endocarditis	92 (4.6)	26 (4.5)	0.004	18 (4.4)	18 (4.4)	<0.001
Emergent operation	32 (1.6)	11 (1.9)	0.024	7 (1.7)	7 (1.7)	<0.001
Coronary artery bypass grafting	363 (18.0)	79 (13.6)	0.120	59 (14.5)	59 (14.5)	<0.001
Valve size (mm)			0.390			<0.001
19	74 (3.7)	32 (5.5)		21 (5.2)	21 (5.2)	
21	376 (18.6)	117 (20.1)		83 (20.4)	83 (20.4)	
23	739 (36.6)	259 (44.6)		176 (43.2)	176 (43.2)	
25	584 (28.9)	157 (27.0)		113 (27.7)	113 (27.7)	
27	248 (12.3)	16 (2.8)		14 (3.5)	14 (3.5)	

Numbers are n (%) unless otherwise specified.
eGFR = estimated glomerular filtration rate, SD = standard deviation, SMD = standardized mean difference

For peer review only

Supplemental Figure 1. Proportion of On-X valves by year of surgery.

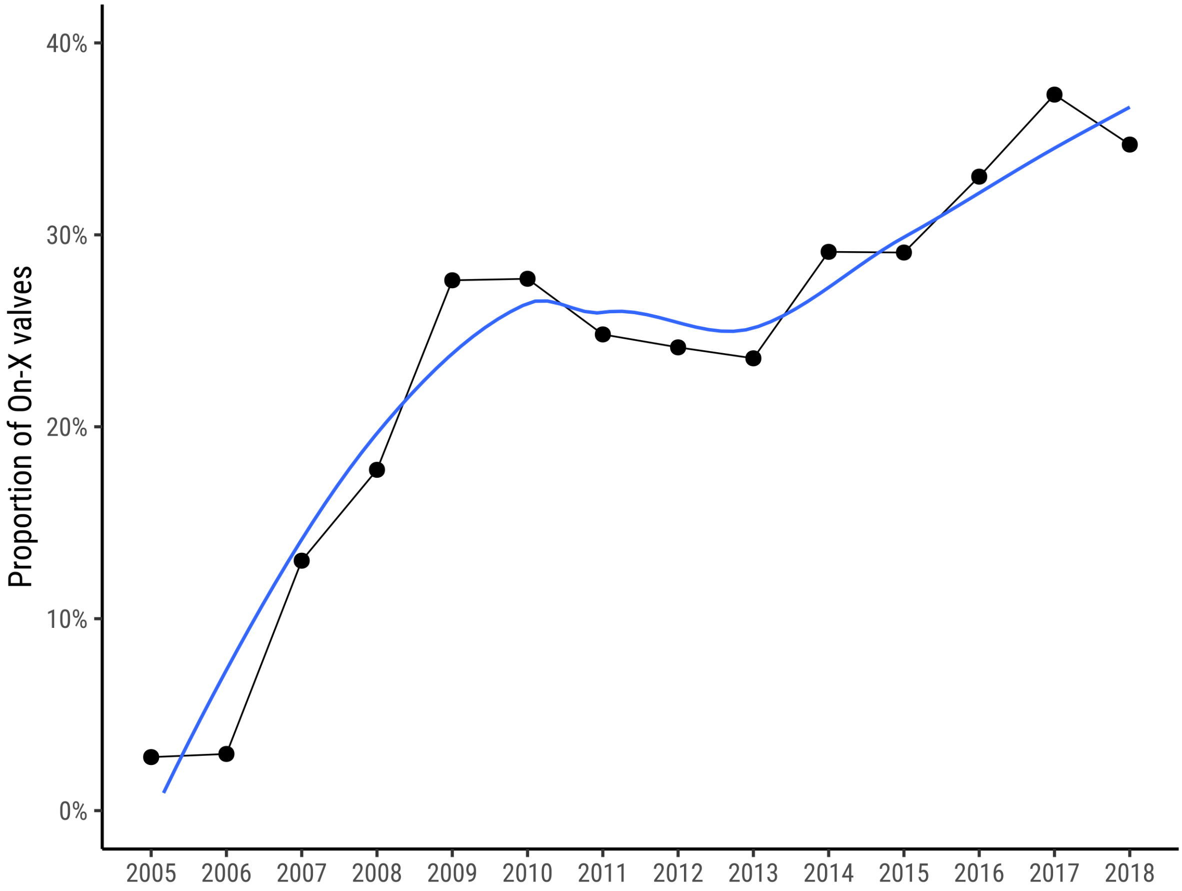

w only

Supplemental Figure 1. Proportion of On-X valves according to hospital.

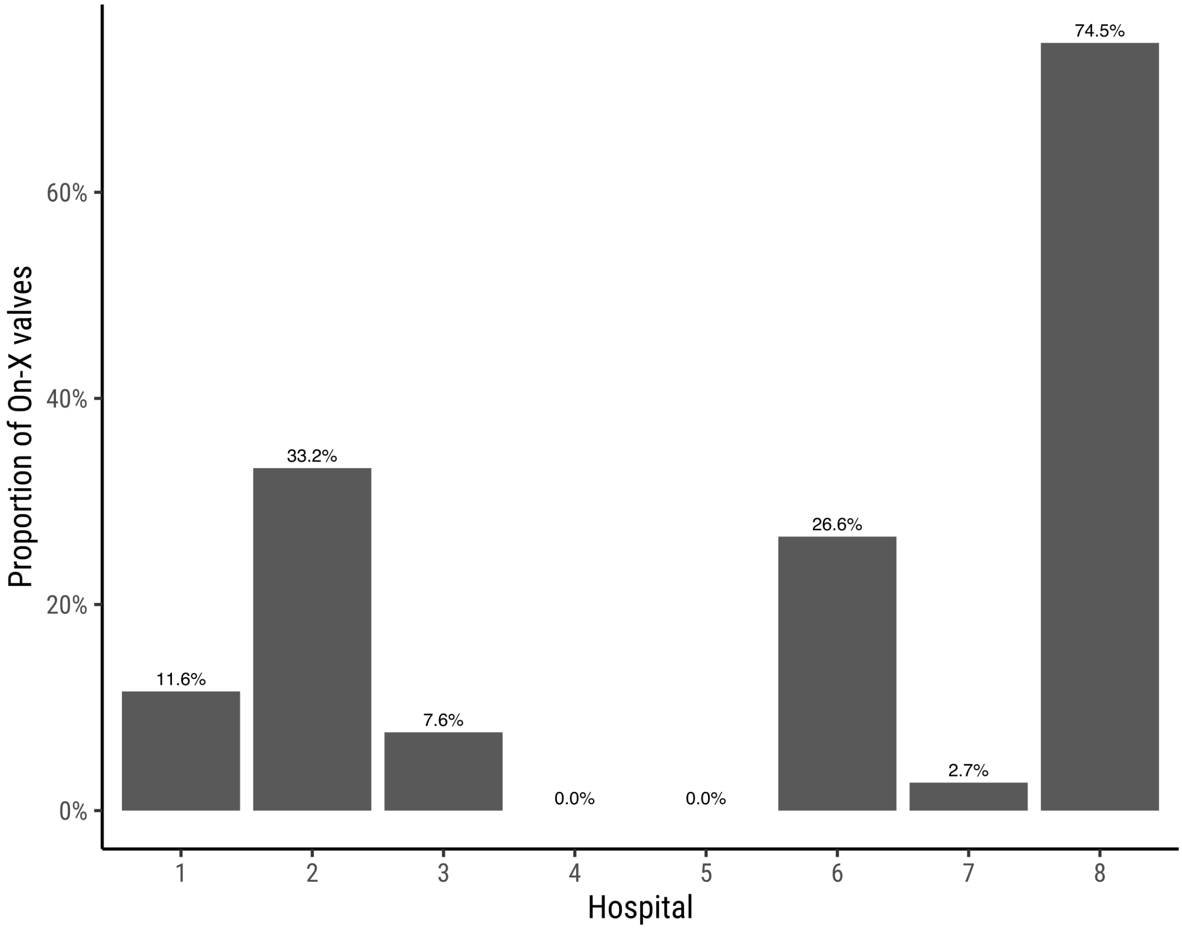

w only

Supplemental Figure 3. Absolute standardized differences before (hollow circles) and after (filled circles) propensity score matching.

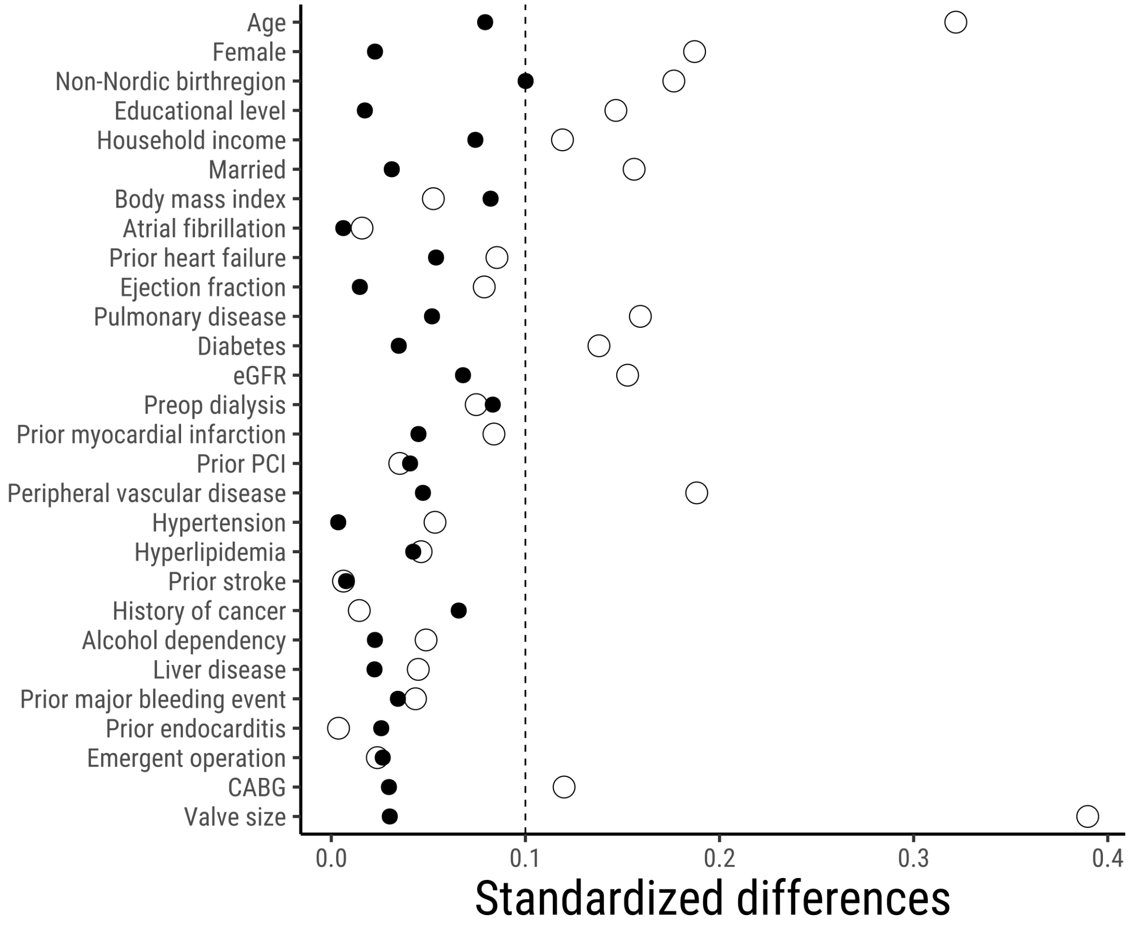

Supplemental Figure 4. Proportion of permanent pacemaker implants by year of surgery.

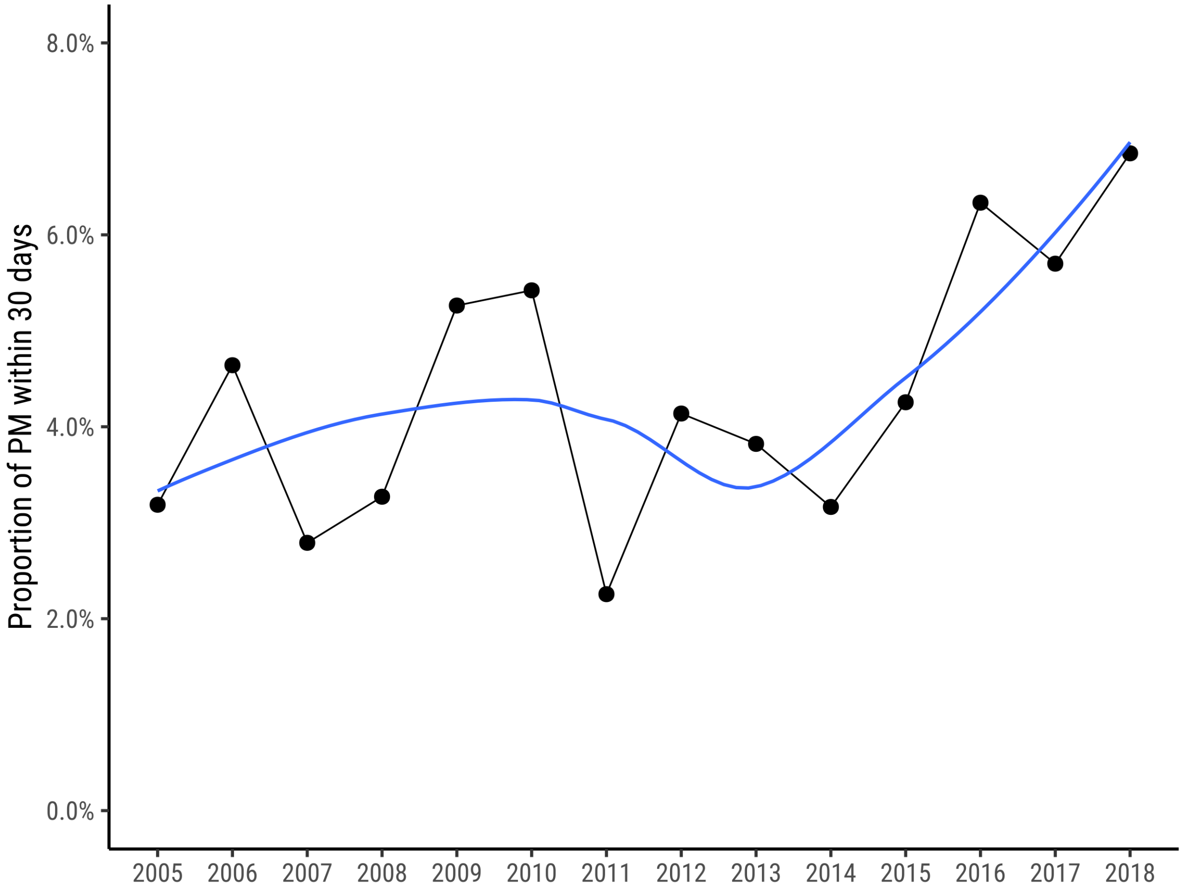

w only

Supplemental Figure 5. Proportion of permanent pacemaker implants according to hospital.

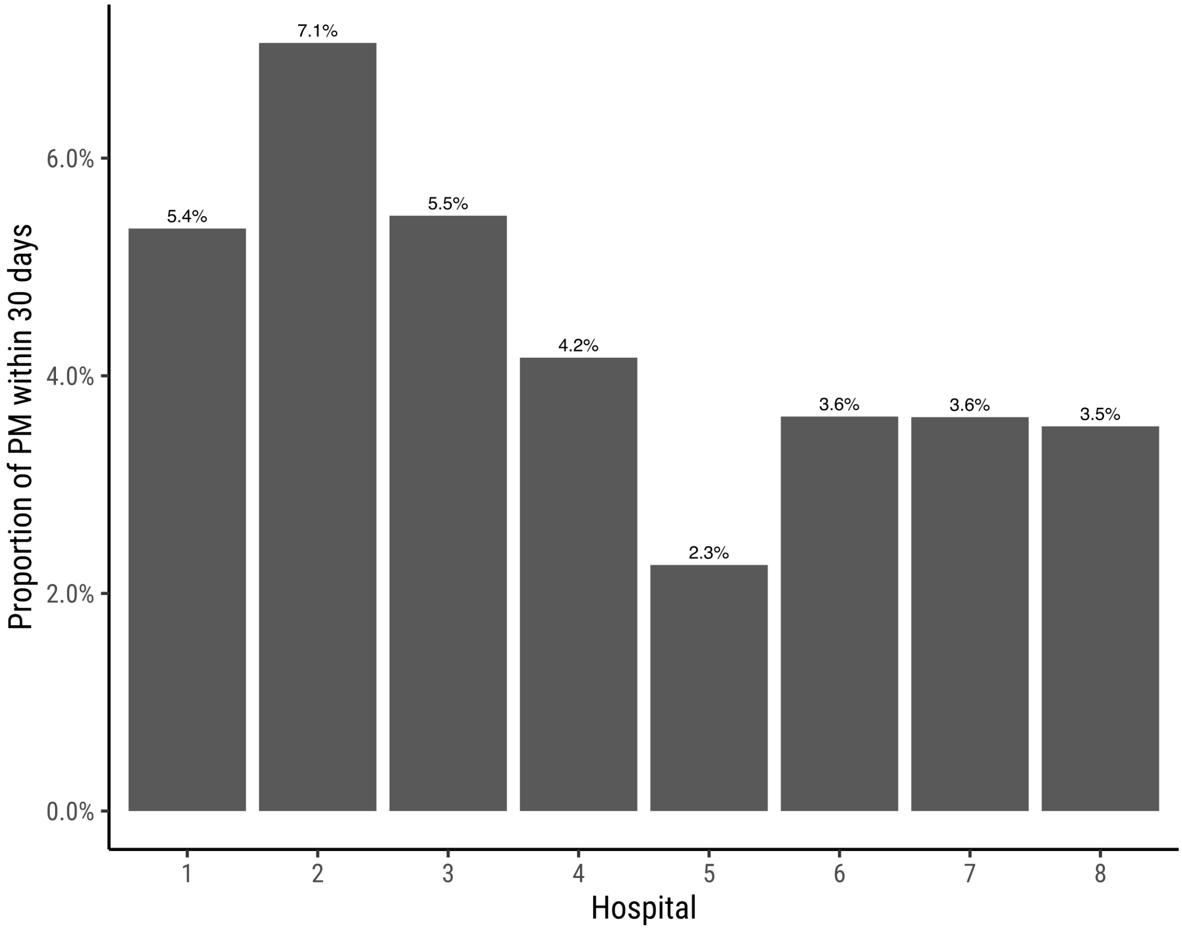

w only

Supplemental Figure 6. Absolute standardized differences before (hollow circles) and after (filled circles) inverse probability of treatment weighting.

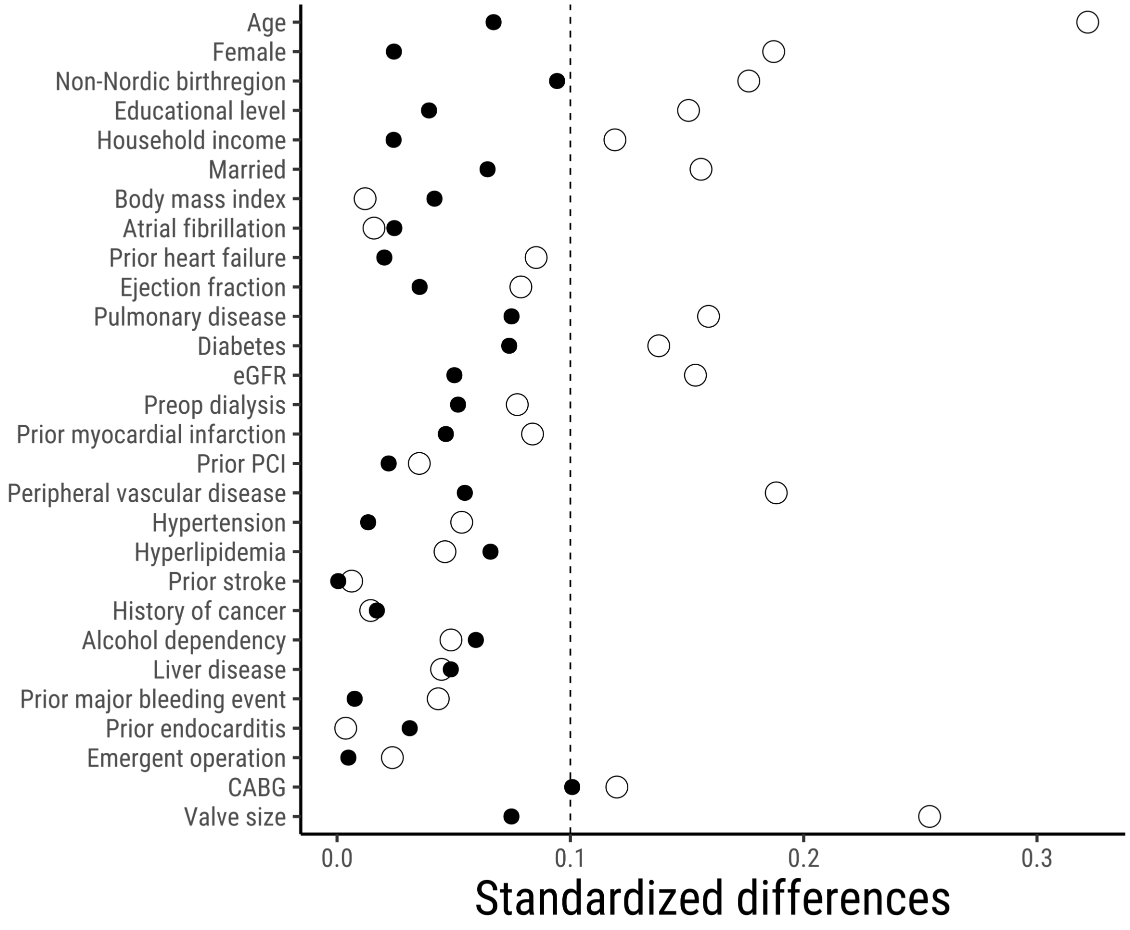

Supplemental Figure 7. Absolute standardized differences before (hollow circles) and after (filled circles) overlap weighting.
Note: This method yields exact covariate balance between treated and reference groups by construction.

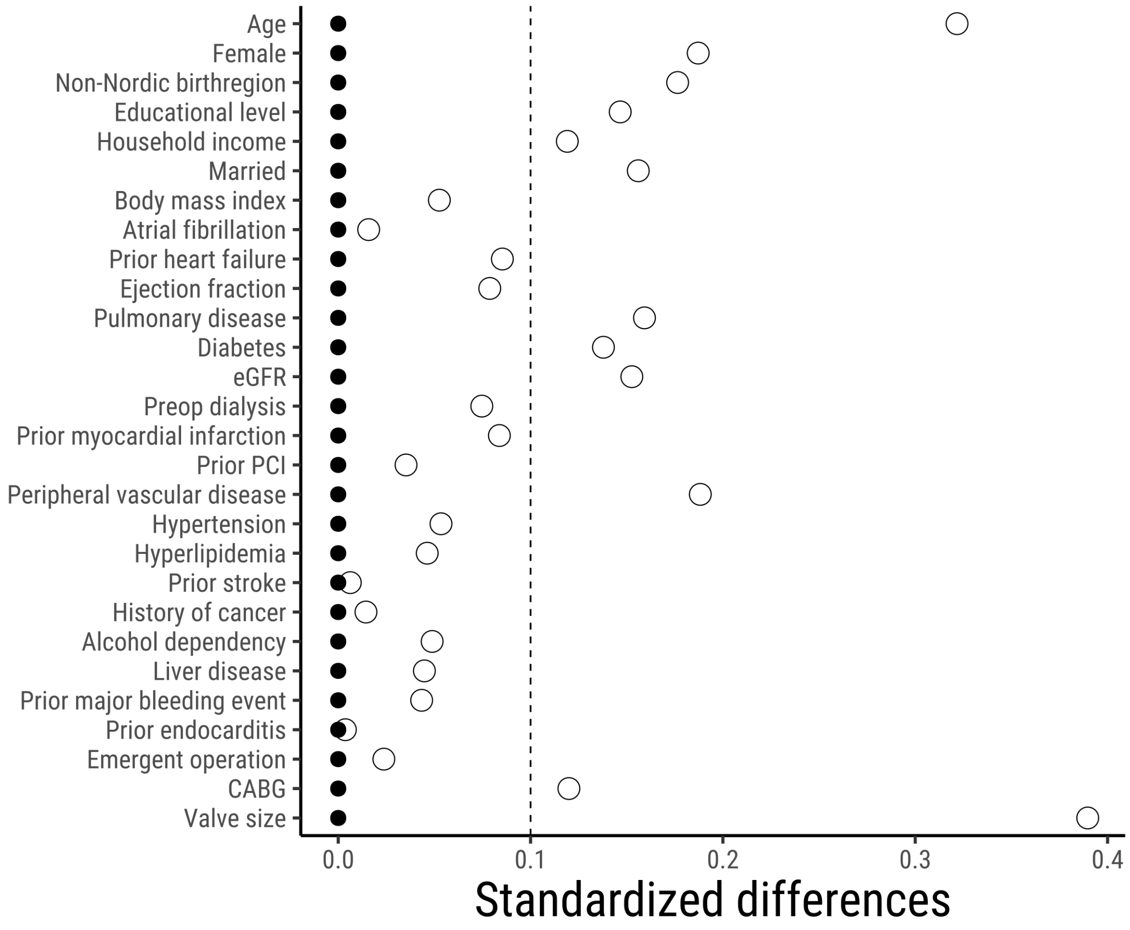

For peer review only

Supplemental Figure 8. Kaplan-Meier estimated pacemaker implantation rate in the inverse probability of treatment weighted population.

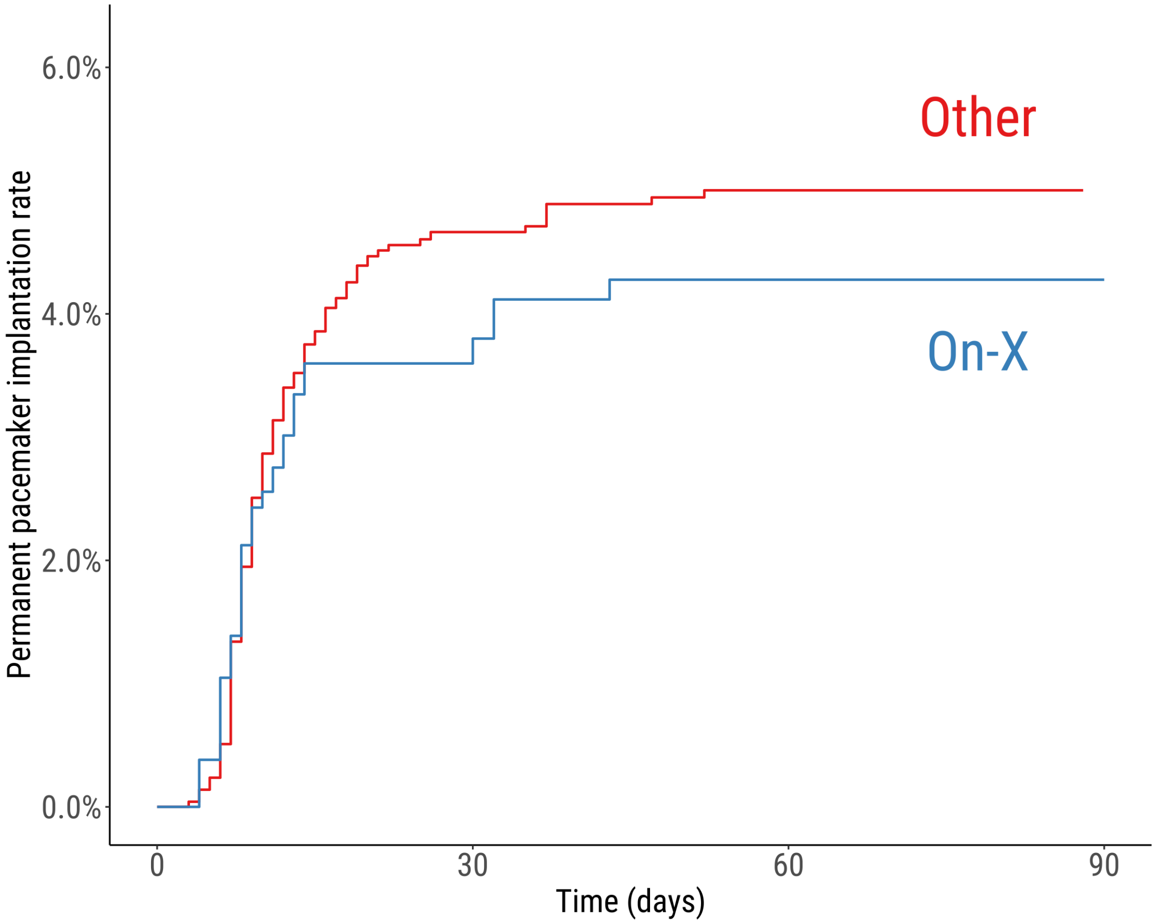

Supplemental Figure 9. Kaplan-Meier estimated pacemaker implantation rate in the overlap weighted population.

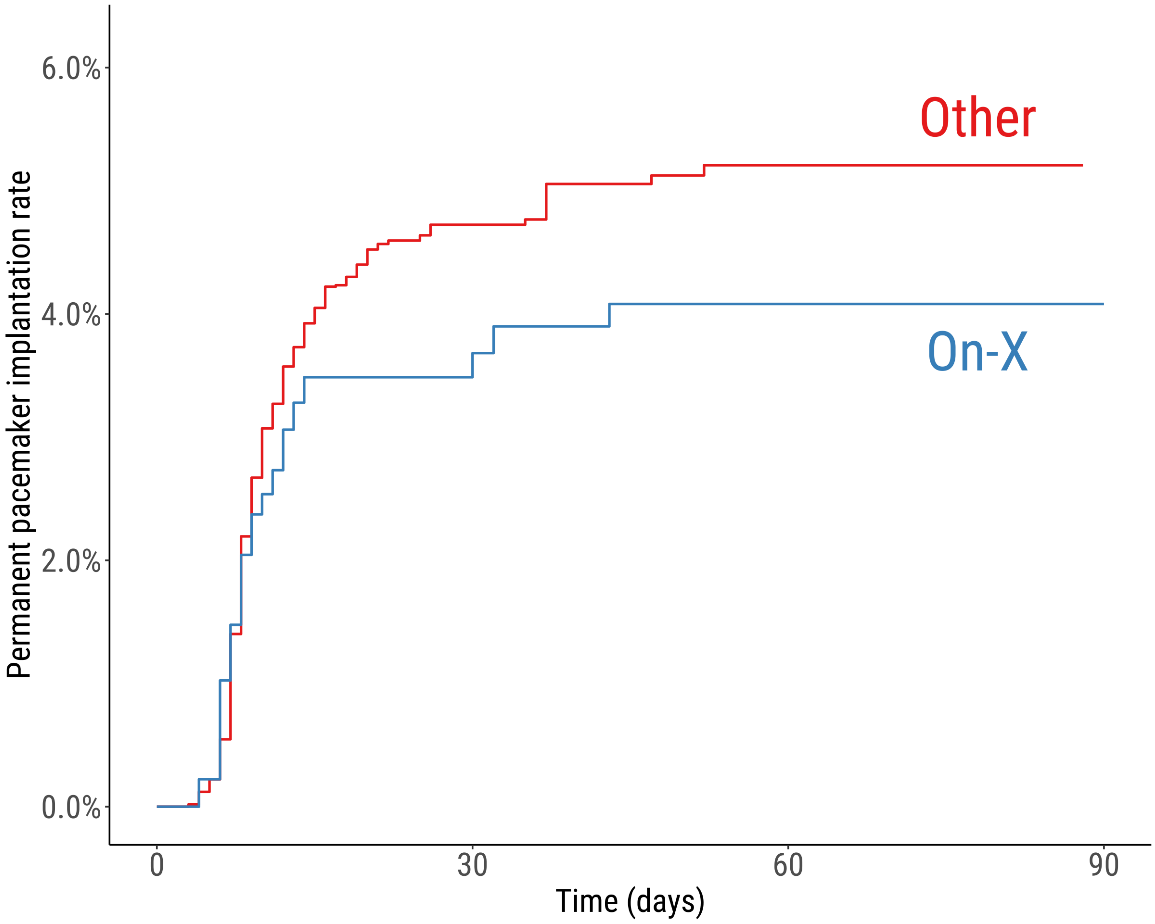

For peer review only

Supplemental Figure 10. Crude and adjusted pacemaker implantation rate after On-X valve aortic valve replacement according to alternative approaches for confounding adjustment.

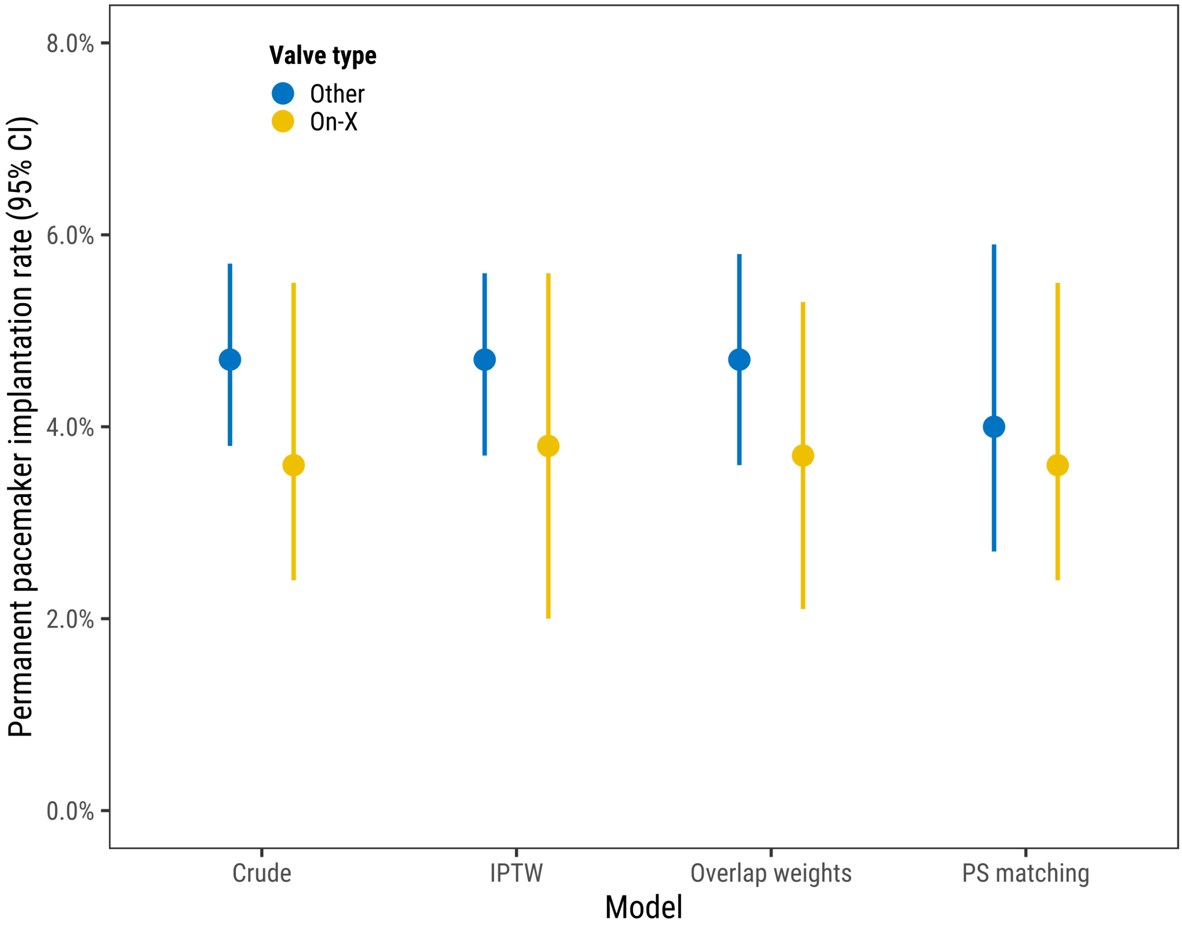

Note: IPTW = inverse probability of treatment weighting, PS = propensity score

only

Reporting checklist for cohort study.

Based on the STROBE cohort guidelines.

Instructions to authors

Complete this checklist by entering the page numbers from your manuscript where readers will find each of the items listed below.

Your article may not currently address all the items on the checklist. Please modify your text to include the missing information. If you are certain that an item does not apply, please write "n/a" and provide a short explanation.

Upload your completed checklist as an extra file when you submit to a journal.

In your methods section, say that you used the STROBE cohortreporting guidelines, and cite them as:

von Elm E, Altman DG, Egger M, Pocock SJ, Gotzsche PC, Vandenbroucke JP. The Strengthening the Reporting of Observational Studies in Epidemiology (STROBE) Statement: guidelines for reporting observational studies.

		Reporting Item	Page Number
**Title and abstract**			
Title	#1a	Indicate the study's design with a commonly used term in the title or the abstract	1
Abstract	#1b	Provide in the abstract an informative and balanced summary of what was done and what was found	2
**Introduction**			
Background / rationale	#2	Explain the scientific background and rationale for the investigation being reported	4
Objectives	#3	State specific objectives, including any prespecified hypotheses	5
**Methods**			
Study design	#4	Present key elements of study design early in the paper	5
Setting	#5	Describe the setting, locations, and relevant dates, including periods	5

For peer review only

		of recruitment, exposure, follow-up, and data collection	
Eligibility criteria	#6a	Give the eligibility criteria, and the sources and methods of selection of participants. Describe methods of follow-up.	5
Eligibility criteria	#6b	For matched studies, give matching criteria and number of exposed and unexposed	5
Variables	#7	Clearly define all outcomes, exposures, predictors, potential confounders, and effect modifiers. Give diagnostic criteria, if applicable	6
Data sources / measurement	#8	For each variable of interest give sources of data and details of methods of assessment (measurement). Describe comparability of assessment methods if there is more than one group. Give information separately for for exposed and unexposed groups if applicable.	5
Bias	#9	Describe any efforts to address potential sources of bias	9
Study size	#10	Explain how the study size was arrived at	n/a
Quantitative variables	#11	Explain how quantitative variables were handled in the analyses. If applicable, describe which groupings were chosen, and why	6
Statistical methods	#12a	Describe all statistical methods, including those used to control for confounding	
6			
Statistical methods	#12b	Describe any methods used to examine subgroups and interactions	6
Statistical methods	#12c	Explain how missing data were addressed	7
Statistical methods	#12d	If applicable, explain how loss to follow-up was addressed	7
Statistical methods	#12e	Describe any sensitivity analyses	
n/a			
**Results**			
Participants	#13a	Report numbers of individuals at each stage of study—eg numbers potentially eligible, examined for eligibility, confirmed eligible,	8

For peer review only

included in the study, completing follow-up, and analysed. Give information separately for for exposed and unexposed groups if applicable.

Participants	#13b	Give reasons for non-participation at each stage	n/a
Participants	#13c	Consider use of a flow diagram	

n/a

Descriptive data	#14a	Give characteristics of study participants (eg demographic, clinical, social) and information on exposures and potential confounders. Give information separately for exposed and unexposed groups if applicable.	8
Descriptive data	#14b	Indicate number of participants with missing data for each variable of interest	

7

Descriptive data	#14c	Summarise follow-up time (eg, average and total amount)	

8

Outcome data	#15	Report numbers of outcome events or summary measures over time. Give information separately for exposed and unexposed groups if applicable.	

8

Main results	#16a	Give unadjusted estimates and, if applicable, confounder-adjusted estimates and their precision (eg, 95% confidence interval). Make clear which confounders were adjusted for and why they were included	8
Main results	#16b	Report category boundaries when continuous variables were categorized	8
Main results	#16c	If relevant, consider translating estimates of relative risk into absolute risk for a meaningful time period	

n/a

Other analyses	#17	Report other analyses done—eg analyses of subgroups and interactions, and sensitivity analyses	9

Discussion

For peer review only

Key results	#18	Summarise key results with reference to study objectives	10
Limitations	#19	Discuss limitations of the study, taking into account sources of potential bias or imprecision. Discuss both direction and magnitude of any potential bias.	11
Interpretation	#20	Give a cautious overall interpretation considering objectives, limitations, multiplicity of analyses, results from similar studies, and other relevant evidence.	11
Generalisability	#21	Discuss the generalisability (external validity) of the study results	11
**Other Information**			
Funding	#22	Give the source of funding and the role of the funders for the present study and, if applicable, for the original study on which the present article is based	13

The STROBE checklist is distributed under the terms of the Creative Commons Attribution License CC-BY. This checklist was completed on 13. December 2020 using https://www.goodreports.org/, a tool made by the EQUATOR Network in collaboration with Penelope.ai

For peer review only

BMJ Open

Permanent pacemaker implantation after On-X surgical aortic valve replacement: SWEDEHEART observational study

Journal:	*BMJ Open*
Manuscript ID	bmjopen-2020-047962.R1
Article Type:	Original research
Date Submitted by the Author:	10-Jul-2021
Complete List of Authors:	Dalén, Magnus; Karolinska Hospital, Department of Cardiothoracic Surgery; Karolinska Institutet, Department of Molecular Medicine and Surgery<br>Persson, Michael; Karolinska University Hospital, Department of Cardiothoracic Surgery; Karolinska Institutet<br>Glaser, Natalie; Karolinska Institutet, Department of Molecular Medicine and Surgery<br>Sartipy, Ulrik; Karolinska Institutet, Department of Cardiothoracic Surgery and Anesthesiology
**Primary Subject Heading**:	Surgery
Secondary Subject Heading:	Cardiovascular medicine, Epidemiology
Keywords:	Valvular heart disease < CARDIOLOGY, Pacing & electrophysiology < CARDIOLOGY, Cardiac surgery < SURGERY

SCHOLARONE™
Manuscripts

I, the Submitting Author has the right to grant and does grant on behalf of all authors of the Work (as defined in the below author licence), an exclusive licence and/or a non-exclusive licence for contributions from authors who are: i) UK Crown employees; ii) where BMJ has agreed a CC-BY licence shall apply, and/or iii) in accordance with the terms applicable for US Federal Government officers or employees acting as part of their official duties; on a worldwide, perpetual, irrevocable, royalty-free basis to BMJ Publishing Group Ltd ("BMJ") its licensees and where the relevant Journal is co-owned by BMJ to the co-owners of the Journal, to publish the Work in this journal and any other BMJ products and to exploit all rights, as set out in our licence.

The Submitting Author accepts and understands that any supply made under these terms is made by BMJ to the Submitting Author unless you are acting as an employee on behalf of your employer or a postgraduate student of an affiliated institution which is paying any applicable article publishing charge ("APC") for Open Access articles. Where the Submitting Author wishes to make the Work available on an Open Access basis (and intends to pay the relevant APC), the terms of reuse of such Open Access shall be governed by a Creative Commons licence – details of these licences and which Creative Commons licence will apply to this Work are set out in our licence referred to above.

Other than as permitted in any relevant BMJ Author's Self Archiving Policies, I confirm this Work has not been accepted for publication elsewhere, is not being considered for publication elsewhere and does not duplicate material already published. I confirm all authors consent to publication of this Work and authorise the granting of this licence.

For peer review only

Permanent pacemaker implantation after On-X surgical aortic valve replacement: SWEDEHEART observational study

Running head: Pacemaker and On-X aortic valve

Magnus Dalén, MD, PhD,[1,2] Michael Persson, MD,[1,2] Natalie Glaser, MD, PhD,[2,3]

Ulrik Sartipy, MD, PhD[1,2]

[1]Department of Cardiothoracic Surgery, Karolinska University Hospital, Stockholm, Sweden

[2]Department of Molecular Medicine and Surgery, Karolinska Institutet, Stockholm, Sweden

[3]Department of Cardiology, Stockholm South General Hospital, Stockholm, Sweden

For peer review only

Disclosures: The authors declare no conflict of interest.

Word count: 4677

Corresponding author:

Magnus Dalén MD, PhD
Department of Cardiothoracic Surgery
Karolinska University Hospital,
SE-171 76 Stockholm, Sweden
Tel.: +46 8 517 708 37
Fax: +46 8 33 19 31
Email address: Magnus.Dalen@ki.se

Abstract

Objective

Bioprosthetic aortic valves with an extended subannular component, such as transcatheter valves, exert increased compression on the cardiac conduction system and increase the risk for permanent pacemaker implantation. It is unknown if the On-X prosthetic valve, which has an elongated subannular valve housing, increases the risk of permanent pacemaker implantation following aortic valve replacement.

Design

Observational nationwide cohort study.

Setting

Swedish population-based study

Participants

All patients aged 18-65 years who underwent primary mechanical aortic valve replacement in Sweden between 2005 and 2018. We used the SWEDEHEART register and other Swedish national health-data registers.

Exposure

Patients implanted with an On-X valve versus patients implanted with other bileaflet mechanical valves.

Primary and secondary outcome measures

Primary outcome measure was permanent pacemaker implantation within 30 days of surgery.

Results

A total of 2602 patients were included, and 581 patients received an On-X valve and 2021 patients received a St Jude Masters/Regent (n=945) or Carbomedics Reduced valve (n=1076). In the total study population, 115 (4.4%) permanent pacemaker implantations were performed within 30 days after aortic valve replacement. In the propensity score matched population, there was no significant difference in the rate of permanent pacemaker implantation in the On-X

For peer review only

group compared to the control group: 3.6% (95% CI: 2.4% to 5.5%) vs. 4.0% (95% CI: 2.7% to 5.9%), p=0.877.

Conclusions

The On-X prosthetic heart valve was associated with a similarly low risk for permanent pacemaker implantation after aortic valve replacement compared with other conventional bileaflet mechanical valves. The On-X elongated subannular valve housing does not interfere with the cardiac conduction system

Key words: Aortic valve replacement; mechanical prosthetic heart valve; pacemaker.

For peer review only

Article summary

Strengths and limitations of this study:

- Data were obtained from high-quality national Swedish health data registers.
- Long and complete follow-up in a nationwide cohort.
- One limitation of the study was that data did not include preoperative conduction disorders known to increase the risk for postoperative pacemaker requirement, such as bundle branch block.
- Another limitation was that we had no information about specific indications of postoperative pacemaker implantation.

For peer review only

Introduction

Surgical aortic valve replacement can induce cardiac conduction disorders that require permanent pacemaker implantation in 2-6% of the patients.[1-4] The close proximity of the atrioventricular node and the bundle of His to the aortic valve annulus and the left ventricular outflow tract makes the cardiac conduction system prone to injury during implantation of an aortic valve prosthesis.[3] Compared with conventional intra-/supraannulary prosthetic valves, bioprosthetic valves with an extended subannular component, such as transcatheter and rapid deployment valves, exerts increased compression on the conduction system.[5, 6] These prosthetic valves, as well as the subannular implantation depth, are associated with markedly increased risks for permanent pacemaker implantation.[5-8] The current standard for mechanical aortic valves used in contemporary clinical practice are bileaflet valves implanted intra- or supraannulary. The On-X prosthetic valve (On-X Life Technologies, Austin, Texas) is one of the most commonly used bileaflet mechanical valves today. Its' design differs from other contemporary mechanical valves,[9] and it is the only mechanical heart valve approved for reduced international normalized ratio target range.[10, 11] In order to reduce turbulence and enhance blood flow over the prosthetic valve, the On-X consists of an elongated valve housing with a flared inlet that protrudes subannulary into the left ventricular outflow tract.[9] It is unknown whether this design increases the risk for conduction disorders requiring permanent pacemaker implantation.

We performed a nationwide population-based cohort study to analyze whether aortic valve replacement with implantation of the On-X prosthetic heart valve, compared with other conventional bileaflet mechanical valves, was associated with an increased risk for permanent pacemaker implantation.

For peer review only

Methods

This nationwide population-based observational cohort study followed the Strengthening the Reporting of Observational Studies in Epidemiology (STROBE) and the REporting of studies Conducted using Observational Routinely collected health Data (RECORD) guidelines for observational studies using routinely collected data.[12, 13] The study was approved by the Swedish Ethical Review Authority and the need for informed consent was waived (Registration number: 2019-04131).

Study population

The Swedish Web system for Enhancement and Development of Evidence-based care in Heart disease Evaluated According to Recommended Therapies (SWEDEHEART) register[14, 15] was used to identify all adult patients aged 18-65 who underwent primary mechanical aortic valve replacement between 2005 and 2018. Patients who had a permanent pacemaker or implantable cardioverter-defibrillator prior to surgery or who underwent valve replacement because of infective endocarditis, or who had concomitant mitral valve surgery were excluded. Implantation of the On-X prosthetic valve was the exposure and the two most common valve types (Carbomedics Reduced [LivaNova, London, United Kingdom] and St Jude Masters/Regent [St Jude Medical, St Paul, Minnesota, USA]) served as the control group. Thus, patients who received other valve models were excluded.

As previously described, individual-level data linking to other nationwide health care registries was performed using the unique personal identity numbers assigned to all Swedish residents.[16, 17] The National Patient Register[18] was used to acquire information regarding relevant prior medical history, and the LISA (Longitudinal integration database for health insurance and labor market studies) database, managed by Statistics Sweden, was used to obtain information regarding socioeconomic variabels.[19]

Outcomes

For peer review only

The primary outcome measure was the implantation of a permanent pacemaker or implantable cardioverter-defibrillator within 30 days following aortic valve replacement as identified by the International Classification of Diseases-codes (FPE00, FPE10, FPE20, FPE26, FPF00, FPF10, FPF20, FPG10, FPG20, FPG30, and FPG33) from the Swedish National Patient Register.[18] We included implantable cardioverter defibrillator in the primary outcome measure in order to capture patients who had dual indications for both pacing and defibrillation.

Statistical methods

Baseline characteristics were described with frequencies and percentages for categorical variables and means and standard deviations for continuous variables. To minimize confounding due to measured baseline covariates, we estimated propensity scores (the probability of receiving an On-X valve based on the observed data) using a logistic regression model that included all variables reported in Table 1. A propensity score matched cohort was created by 1:1 nearest neighbor matching on the logit of the propensity score without replacement and a caliper width of 0.2 times the standard deviation of the logit of the propensity score.[20] In a separate analysis, we also estimated propensity scores using generalized boosted regression modeling[21, 22] and used the scores for inverse probability of treatment weighting. Finally, we applied the propensity scores from a logistic regression model to construct overlap weights, a weighting method that yields exact covariate balance between the exposed and reference groups by construction.[23] Balance between the groups was assessed by standardized mean differences. An absolute standardized difference ≤0.1 was considered an ideal balance.[24] In the matched and weighed populations, respectively, the percentage of pacemaker implants and 95% confidence interval was compared between the groups using McNemar's and chi-square tests, respectively. To illustrate the pacemaker implantation rate the first 90 days postoperatively, we constructed Kaplan-Meier curves. Although death could be considered a competing event, it occurred rarely during the first 90 days of follow-up, and was therefore not accounted for. The statistical analyses were performed with R version 4.0.2 (R

For peer review only

Foundation for Statistical Computing, Vienna, Austria) and the Matching[25] and twang[22] packages.

Missing data

Although data were complete for most variables, including exposure and outcome, there were some missing data. The variables with missing data were body mass index, estimated glomerular filtration rate, valve size, and educational level with 6.9%, 1.5%, 0.7%, and 0.6% missing data, respectively. For these variables, missing data was imputed with the most common category. Missing data was handled differently in the inverse probability of treatment weighted analysis because the weights were constructed to also balance rates of missingness in both groups.[21, 22]

Patient and Public Involvement

There was no patient or public involvement in the design or conduct of this study.

For peer review only

Results

A total of 2602 patients were included in the study, and 581 patients received an On-X valve and 2021 patients received a St Jude Masters/Regent (n=945) or Carbomedics Reduced valve (n=1076) at the eight hospitals performing cardiac surgery in Sweden during the study period. The proportion of On-X valve implantations increased during the study period but varied markedly by hospital as shown in Supplemental Figure 1-2 and Supplemental Table 1-2. The mean age in the total study population was 53.4 years, and 24% were women. Before propensity score matching, there were differences in baseline characteristics between the groups as shown in Table 1. There were more women and the mean age was higher in the control group. Peripheral vascular disease was more common in the On-X valve group, whereas pulmonary disease, diabetes, and concomitant coronary artery bypass grafting was more common in the control group. The 30-day all-cause mortality was 0.69% in the On-X valve group vs. 0.64% in the control group (p=0.905). After propensity score matching, the two groups were well balanced across all baseline characteristics and no standardized mean differences were greater than 10% (Table 1 and Supplemental Figure 3-4).

Permanent pacemaker implantation following mechanical aortic valve replacement

In the total study population, 115 (4.4%) permanent pacemaker implantations were performed within 30 days after aortic valve replacement. In the On-X group, the number of pacemakers implanted was 21 (3.6%) vs. 94 (4.6%) in the control group (p=0.284). Implantable cardioverter-defibrillators accounted for 5% of the implantations in both groups. The pacemaker implantation rate increased slightly during the study period but was fairly similar between hospitals (Supplemental Figure 5-6 and Supplemental Table 3-4).

The Kaplan-Meier estimated rate of permanent pacemaker implantation in the propensity score matched population is shown in Figure 1. There was no significant difference in the rate of permanent pacemaker implantation within 30 days after aortic valve replacement with the On-X valve compared to the control group: 3.6% (95% CI: 2.4% to 5.5%) vs. 4.0% (95% CI: 2.7% to

For peer review only

5.9%), p=0.877. The odds ratio (95% CI) for a permanent pacemaker implantation within 30 days after On-X aortic valve replacement was 0.91 (0.49-1.67). The majority of permanent pacemaker implantations occurred during the first 30 days. No pacemakers were implanted from postoperative day 60 to postoperative day 120.

Alternative approaches for confounding adjustment

Two alternative approaches for confounding adjustment using propensity scores for construction of weights were conducted: inverse probability of treatment weighting and overlap weighting. Both approaches resulted in excellent balance in baseline characteristics between the groups (Supplemental Table 5-6 and Supplemental Figure 7-8). The results were very similar to the results obtained in the propensity score matched analysis, and we found no significant difference in the rate of permanent pacemaker implantation within 30 days after aortic valve replacement with an On-X valve compared to the control group.

In the inverse probability of treatment weighted population, the rate of permanent pacemaker implantation within 30 days after aortic valve replacement was 3.8% (95% CI: 2.0% to 5.6%) with an On-X valve vs. 4.7% (95% CI: 3.7% to 5.6%) in the control group, p = 0.428. The odds ratio (95% CI) for a permanent pacemaker implantation within 30 days after On-X aortic valve replacement was 0.81 (0.48-1.37).

In the population where overlap weights were used, the rate of permanent pacemaker implantation within 30 days after aortic valve replacement was 3.7% (95% CI: 2.1% to 5.3%) with an On-X valve vs. 4.7% (95% CI: 3.6% to 5.8%) in the control group, p = 0.357. The odds ratio (95% CI) for a permanent pacemaker implantation within 30 days after On-X aortic valve replacement was 0.79 (0.47-1.31).

The Kaplan-Meier estimated rates of permanent pacemaker implantation in the inverse probability of treatment weighted population, and the population where overlap weights were used, respectively, are shown in Supplemental Figure 9-10.

For peer review only

The results from all approaches are summarized in Supplemental Figure 11. As height and weight were not included in the propensity score model, these data are presented in Supplemental Table 7.

For peer review only

Discussion

The On-X prosthetic heart valve has an elongated subannular valve housing with a flared inlet that protrudes subannulary into the left ventricular outflow tract. This design distinguishes the On-X prosthetic valve from other contemporary bileaflet mechanical aortic valves that are implanted intra-/supraannulary and that do not extend subannulary (Figure 2). In this nationwide cohort study, aortic valve replacement with implantation of the On-X prosthetic heart valve was not associated with an increased risk for permanent pacemaker implantation compared with other intra-/supraannulary bileaflet mechanical valves.

Cardiac conduction abnormalities requiring permanent pacemaker implantation is a well-known complication of aortic valve replacement.[1] The widespread use of transcatheter and rapid deployment prosthetic valves, which are associated with a significant risk of injury to the conduction system, has generated an increased interest in research concerning permanent pacemaker implantation after aortic valve replacement.[5-7] Although the use of mechanical prosthetic valves has decreased during later years, these valves are still the primary choice for younger patients, for whom mechanical valves have been shown to be superior to biological valves, in terms of mortality and reoperation rates.[17, 26] Permanent pacemaker requirement has in some studies been associated with adverse events and impaired long-term survival.[2] The potential risks associated with permanent pacemaker requirement are particularly relevant for younger patients since they have a longer life expectancy.[27]

The incidence of permanent pacemaker implantation in the current study (4.4%) is in line with previous studies of mechanical aortic valve replacement in younger adults, reporting rates of 2.5-5%.[28, 29] Few head-to-head comparisons of mechanical bileaflet prosthetic valves have been reported[30] and none of these have analyzed permanent pacemaker implantation rates between different valves. Generally, there are very few reports regarding rates of permanent pacemaker implantation after mechanical aortic valve replacement and most prior studies concerning mechanical prosthetic valves are completely lacking information on permanent pacemaker rates.

For peer review only

Compression on the subannunlar tissue, and thereby subsequent possible compression of the cardiac conduction system can induce conduction disturbances necessitating permanent pacemaker implantation. Because bioprosthetic valves with an extended subannular component, such as transcatheter and rapid deployment prosthetic valves, have been associated with a markedly increased risk for permanent pacemaker implantation,[5-7] we aimed to analyze whether this would also be true for the On-X prosthetic valve. Despite its' elongated subannular valve housing, our results demonstrated that the On-X prosthetic heart valve did not confer a higher risk of permanent pacemaker implantation compared with other conventional intra-/supraannulary bileaflet mechanical valves. This might be explained by the differences in design and annular fixation between the On-X prosthetic valve and transcatheter/rapid deployment prosthetic valves. These valves, unlike the On-X, are balloon- or self-expandable bioprosthetic valves that rely on radial forces for deployment of a subannular frame for stabilization in the aortic annulus and left ventricular outflow tract. These valves are therefore oversized in relation to the aortic annulus. This is in contrast with the On-X valve which is sutured to the aortic annulus with no expansion of the subannular valve component. Correct implantation requires that the On-X subannular valve housing fit into the left ventricular outflow tract and oversizing is thereby not possible.

Limitations

This analysis has limitations. First, the data did not include preoperative conduction disorders known to increase the risk for postoperative pacemaker requirement, such as bundle branch block, as well as other risk factors for postoperative pacemaker requirement such as mitral annular calcification, left ventricular outflow tract calcification, and surgical suture technique that was used for valve implantation. We were therefore not able to adjust for such risk factors. We were unable to account for possible center effects, owing to a low number of events (pacemaker implantations) in the On-X group. Second, we did not have information about specific indications of postoperative pacemaker implantation. Third, we included implantable

For peer review only

cardiac defibrillators in the primary outcome measure, in order to capture patients who had dual indications for both pacing and defibrillation. It is possible that some of these patients only had indication for defibrillation.

Conclusions

The On-X prosthetic heart valve was associated with a similarly low risk for permanent pacemaker implantation after aortic valve replacement compared with other conventional bileaflet mechanical valves. This indicates that the On-X elongated subannular valve housing does not seem to inflict on the cardiac conduction system.

For peer review only

Data availability statement

All data relevant to the study are included in the article or uploaded as supplementary information.

Competing Interest statement

There are no competing interests for any author.

Funding

This work was supported by a donation from Mr. Fredrik Lundberg (to MD, no grant number), the Swedish Heart-Lung Foundation (grant numbers 20180400 and 20190533 to US, and 20190570 to NG), Åke Wiberg Foundation (grant number M18-0016 to US), Region Stockholm (ALF project) (grant number 20180114 to US), the Swedish Society of Medicine (grant number SLS-934749 to NG), the Capio Research Foundation (grant number 2019-3239 to NG), the Eva and Oscar Ahrén Research Foundation (to NG), and the Seraphim Hospital Foundation (to NG).

Acknowledgments

We thank the SWEDEHEART steering committee for providing data for this study.

Contributorship statement

All authors (MD, MP, NG, US) have made substantial contributions to the conception or design of the work; or the acquisition, analysis, or interpretation of data for the work; and drafting the work and revising it critically for important intellectual content; and made final approval of the version to be published; and agreed to be accountable for all aspects of the work in ensuring that questions related to the accuracy or integrity of any part of the work are appropriately investigated and resolved.

For peer review only

Ethical approval statement

The study was approved by the Swedish Ethical Review Authority and the need for informed consent was waived (Registration number: 2019-04131).

For peer review only

References

1. Moskowitz G, Hong KN, Giustino G, et al. Incidence and Risk Factors for Permanent Pacemaker Implantation Following Mitral or Aortic Valve Surgery. J Am Coll Cardiol. 2019;74:2607-2620
2. Mehaffey JH, Haywood NS, Hawkins RB, et al. Need for Permanent Pacemaker After Surgical Aortic Valve Replacement Reduces Long-Term Survival. Ann Thorac Surg. 2018;106:460-465
3. Wiggins NB, Chong DT, Houghtaling PL, et al. Incidence, indications, risk factors, and survival of patients undergoing cardiac implantable electronic device implantation after open heart surgery. Europace. 2017;19:1335-1342
4. Levack MM, Kapadia SR, Soltesz EG, et al. Prevalence of and Risk Factors for Permanent Pacemaker Implantation After Aortic Valve Replacement. Ann Thorac Surg. 2019;108:700-707
5. Coti I, Schukro C, Drevinja F, et al. Conduction disturbances following surgical aortic valve replacement with a rapid-deployment bioprosthesis. J Thorac Cardiovasc Surg. 2020 DOI:10.1016/j.jtcvs.2020.01.083
6. van der Boon RM, Nuis RJ, Van Mieghem NM, et al. New conduction abnormalities after TAVI--frequency and causes. Nat Rev Cardiol. 2012;9:454-463
7. Bouhout I, Mazine A, Rivard L, et al. Conduction Disorders After Sutureless Aortic Valve Replacement. Ann Thorac Surg. 2017;103:1254-1260
8. Fraccaro C, Napodano M, Tarantini G. Conduction disorders in the setting of transcatheter aortic valve implantation: a clinical perspective. Catheter Cardiovasc Interv. 2013;81:1217-1223
9. Chaudhary R, Garg J, Krishnamoorthy P, et al. On-X Valve: The Next Generation Aortic Valve. Cardiol Rev. 2017;25:77-83

For peer review only

10. Puskas J, Gerdisch M, Nichols D, et al. Reduced anticoagulation after mechanical aortic valve replacement: interim results from the prospective randomized on-X valve anticoagulation clinical trial randomized Food and Drug Administration investigational device exemption trial. J Thorac Cardiovasc Surg. 2014;147:1202-1210

11. Puskas JD, Gerdisch M, Nichols D, et al. Anticoagulation and Antiplatelet Strategies After On-X Mechanical Aortic Valve Replacement. J Am Coll Cardiol. 2018;71:2717-2726

12. Benchimol EI, Smeeth L, Guttmann A, et al. The REporting of studies Conducted using Observational Routinely-collected health Data (RECORD) statement. PLoS Med. 2015;12:e1001885

13. von Elm E, Altman DG, Egger M, et al. The Strengthening the Reporting of Observational Studies in Epidemiology (STROBE) statement: guidelines for reporting observational studies. J Clin Epidemiol. 2008;61:344-349

14. Jernberg T, Attebring MF, Hambraeus K, et al. The Swedish Web-system for Enhancement and Development of Evidence-based care in Heart disease Evaluated According to Recommended Therapies (SWEDEHEART). Heart. 2010;96:1617-1621

15. Vikholm P, Ivert T, Nilsson J, et al. Validity of the Swedish Cardiac Surgery Registry. Interact Cardiovasc Thorac Surg. 2018;27:67-74

16. Ludvigsson JF, Otterblad-Olausson P, Pettersson BU, Ekbom A. The Swedish personal identity number: possibilities and pitfalls in healthcare and medical research. Eur J Epidemiol. 2009;24:659-667

17. Glaser N, Jackson V, Holzmann MJ, Franco-Cereceda A, Sartipy U. Aortic valve replacement with mechanical vs. biological prostheses in patients aged 50-69 years. Eur Heart J. 2016;37:2658-2667

18. Ludvigsson JF, Andersson E, Ekbom A, et al. External review and validation of the Swedish national inpatient register. BMC Public Health. 2011;11:450

For peer review only

19. Ludvigsson JF, Svedberg P, Olen O, Bruze G, Neovius M. The longitudinal integrated database for health insurance and labour market studies (LISA) and its use in medical research. Eur J Epidemiol. 2019;34:423-437

20. Austin PC. Optimal caliper widths for propensity-score matching when estimating differences in means and differences in proportions in observational studies. Pharm Stat. 2011;10:150-161

21. Griffin BA, Ridgeway G, Morral A, et al. Toolkit for Weighting and Analysis of Nonequivalent Groups (TWANG) Website. http://www.rand.org/statistics/twang

22. Ridgeway G, McCaffrey D, Morral A, Griffin BA, Burgette L. twang: Toolkit for Weighting and Analysis of Nonequivalent Groups. https://CRAN.R-project.org/package=twang

23. Desai RJ, Franklin JM. Alternative approaches for confounding adjustment in observational studies using weighting based on the propensity score: a primer for practitioners. BMJ. 2019;367:l5657

24. Austin PC, Stuart EA. Moving towards best practice when using inverse probability of treatment weighting (IPTW) using the propensity score to estimate causal treatment effects in observational studies. Stat Med. 2015;34:3661-3679

25. Sekhon JS. Multivariate and Propensity Score Matching Software with Automated Balance Optimization: The Matching package for R. Journal of Statistical Software. 2011;42:1-52

26. Goldstone AB, Chiu P, Baiocchi M, et al. Mechanical or Biologic Prostheses for Aortic-Valve and Mitral-Valve Replacement. N Engl J Med. 2017;377:1847-1857

27. Glaser N, Persson M, Jackson V, Holzmann MJ, Franco-Cereceda A, Sartipy U. Loss in Life Expectancy After Surgical Aortic Valve Replacement: SWEDEHEART Study. J Am Coll Cardiol. 2019;74:26-33

28. Korteland NM, Etnel JRG, Arabkhani B, et al. Mechanical aortic valve replacement in non-elderly adults: meta-analysis and microsimulation. Eur Heart J. 2017;38:3370-3377

For peer review only

29. Bouhout I, Stevens LM, Mazine A, et al I. Long-term outcomes after elective isolated mechanical aortic valve replacement in young adults. J Thorac Cardiovasc Surg. 2014;148:1341-1346 e1341

30. Hatoum H, Maureira P, Dasi LP. A turbulence in vitro assessment of On-X and St Jude Medical prostheses. J Thorac Cardiovasc Surg. 2019. DOI: 10.1016/j.jtcvs.2019.02.046

For peer review only

Figure legends

Figure 1. The graph shows the Kaplan-Meier estimated permanent pacemaker implantation rate in propensity score matched patients who received an On-X valve compared to other valves. There was no significant difference in the pacemaker implant rate between the groups. The majority of permanent pacemaker implantations occurred during the first 30 days. No pacemakers were implanted from day 60 to day 120.

Figure 2. The On-X prosthetic heart valve (On-X Life Technologies, Austin, Texas, USA; left panel) has an elongated subannular valve housing with a flared inlet that protrudes subannulary into the left ventricular outflow tract, in close proximity to the cardiac conduction system. This design distinguishes the On-X prosthetic valve from other contemporary bileaflet mechanical aortic valves, such as the St Jude Regent (St Jude Medical, St Paul, Minnesota, USA; right panel), that are implanted intra-/supraannulary and do not extend subannulary. Illustration: Magnus Dalén.

For peer review only

Table 1. Baseline characteristics in patients who underwent aortic valve replacement in Sweden with an On-X valve or other mechanical valves before and after propensity score matching

	Before matching				After propensity score matching			
	Overall	Control	On-X	SMD	Overall	Control	On-X	SMD
Number of patients	2602	2021	581		1160	580	580	
Age (years), mean (SD)	53.4 (9.5)	54.1 (9.0)	51.0 (10.7)	0.322	51.4 (10.6)	51.9 (10.6)	51.0 (10.6)	0.079
Female sex	631 (24.3)	525 (26.0)	106 (18.2)	0.187	207 (17.8)	101 (17.4)	106 (18.3)	0.023
Non-Nordic birth region	239 (9.2)	207 (10.2)	32 (5.5)	0.176	52 (4.5)	20 (3.4)	32 (5.5)	0.100
Educational level				0.147				0.017
<10 years	720 (27.7)	588 (29.1)	132 (22.7)		266 (22.9)	135 (23.3)	131 (22.6)	
10-12 years	1263 (48.5)	959 (47.5)	304 (52.3)		604 (52.1)	300 (51.7)	304 (52.4)	
>12 years	619 (23.8)	474 (23.5)	145 (25.0)		290 (25.0)	145 (25.0)	145 (25.0)	
Disposable household income (quartiles)				0.119				0.074
Q1 (low)	651 (25.0)	520 (25.7)	131 (22.5)		260 (22.4)	129 (22.2)	131 (22.6)	
Q2	651 (25.0)	506 (25.0)	145 (25.0)		289 (24.9)	144 (24.8)	145 (25.0)	
Q3	650 (25.0)	512 (25.3)	138 (23.8)		262 (22.6)	124 (21.4)	138 (23.8)	
Q4 (high)	650 (25.0)	483 (23.9)	167 (28.7)		349 (30.1)	183 (31.6)	166 (28.6)	
Married	1326 (51.0)	1065 (52.7)	261 (44.9)	0.156	531 (45.8)	270 (46.6)	261 (45.0)	0.031
Body mass index (tertiles)				0.053				0.082
T1 (low)	808 (31.1)	623 (30.8)	185 (31.8)		387 (33.4)	203 (35.0)	184 (31.7)	
T2	987 (37.9)	778 (38.5)	209 (36.0)		418 (36.0)	209 (36.0)	209 (36.0)	
T3 (high)	807 (31.0)	620 (30.7)	187 (32.2)		355 (30.6)	168 (29.0)	187 (32.2)	
Atrial fibrillation	233 (9.0)	183 (9.1)	50 (8.6)	0.016	99 (8.5)	49 (8.4)	50 (8.6)	0.006
Heart failure	382 (14.7)	310 (15.3)	72 (12.4)	0.085	134 (11.6)	62 (10.7)	72 (12.4)	0.054
Left ventricular ejection fraction (%)				0.079				0.015
>50	1953 (75.1)	1518 (75.1)	435 (74.9)		872 (75.2)	437 (75.3)	435 (75.0)	
30-50	511 (19.6)	389 (19.2)	122 (21.0)		239 (20.6)	118 (20.3)	121 (20.9)	
<30	138 (5.3)	114 (5.6)	24 (4.1)		49 (4.2)	25 (4.3)	24 (4.1)	
Chronic obstructive pulmonary disease	155 (6.0)	136 (6.7)	19 (3.3)	0.159	33 (2.8)	14 (2.4)	19 (3.3)	0.052

Diabetes	361 (13.9)	301 (14.9)	60 (10.3)	0.138	114 (9.8)	54 (9.3)	60 (10.3)	0.035
eGFR (ml/min/1,73 m^2)				0.153				0.068
>60	2457 (94.4)	1894 (93.7)	563 (96.9)		1124 (96.9)	562 (96.9)	562 (96.9)	
45-59	97 (3.7)	85 (4.2)	12 (2.1)		22 (1.9)	10 (1.7)	12 (2.1)	
30-44	28 (1.1)	24 (1.2)	4 (0.7)		11 (0.9)	7 (1.2)	4 (0.7)	
<30	20 (0.8)	18 (0.9)	2 (0.3)		3 (0.3)	1 (0.2)	2 (0.3)	
Preoperative dialysis	21 (0.8)	19 (0.9)	2 (0.3)	0.075	2 (0.2)	0 (0.0)	2 (0.3)	0.083
Prior myocardial infarction	235 (9.0)	193 (9.5)	42 (7.2)	0.084	91 (7.8)	49 (8.4)	42 (7.2)	0.045
Prior percutaneous coronary intervention	127 (4.9)	102 (5.0)	25 (4.3)	0.035	55 (4.7)	30 (5.2)	25 (4.3)	0.041
Peripheral vascular disease	305 (11.7)	208 (10.3)	97 (16.7)	0.188	184 (15.9)	87 (15.0)	97 (16.7)	0.047
Hypertension	920 (35.4)	703 (34.8)	217 (37.3)	0.053	431 (37.2)	215 (37.1)	216 (37.2)	0.004
Hyperlipidemia	433 (16.6)	344 (17.0)	89 (15.3)	0.046	187 (16.1)	98 (16.9)	89 (15.3)	0.042
Prior stroke	136 (5.2)	105 (5.2)	31 (5.3)	0.006	63 (5.4)	32 (5.5)	31 (5.3)	0.008
History of cancer	128 (4.9)	98 (4.8)	30 (5.2)	0.014	69 (5.9)	39 (6.7)	30 (5.2)	0.066
Alcohol dependence	84 (3.2)	69 (3.4)	15 (2.6)	0.049	28 (2.4)	13 (2.2)	15 (2.6)	0.022
Liver disease	21 (0.8)	18 (0.9)	3 (0.5)	0.045	7 (0.6)	4 (0.7)	3 (0.5)	0.022
Prior bleeding event	82 (3.2)	67 (3.3)	15 (2.6)	0.043	27 (2.3)	12 (2.1)	15 (2.6)	0.034
Prior endocarditis	118 (4.5)	92 (4.6)	26 (4.5)	0.004	49 (4.2)	23 (4.0)	26 (4.5)	0.026
Emergent operation	43 (1.7)	32 (1.6)	11 (1.9)	0.024	20 (1.7)	9 (1.6)	11 (1.9)	0.026
Coronary artery bypass grafting	442 (17.0)	363 (18.0)	79 (13.6)	0.120	164 (14.1)	85 (14.7)	79 (13.6)	0.030
Valve size (mm)				0.390				0.030
19	106 (4.1)	74 (3.7)	32 (5.5)		63 (5.4)	31 (5.3)	32 (5.5)	
21	493 (18.9)	376 (18.6)	117 (20.1)		238 (20.5)	121 (20.9)	117 (20.2)	
23	998 (38.4)	739 (36.6)	259 (44.6)		520 (44.8)	262 (45.2)	258 (44.5)	
25	741 (28.5)	584 (28.9)	157 (27.0)		308 (26.6)	151 (26.0)	157 (27.1)	
27	264 (10.1)	248 (12.3)	16 (2.8)		31 (2.7)	15 (2.6)	16 (2.8)	

Numbers are n (%) unless otherwise specified.

eGFR = estimated glomerular filtration rate, SD = standard deviation, SMD = standardized mean difference

For peer review only

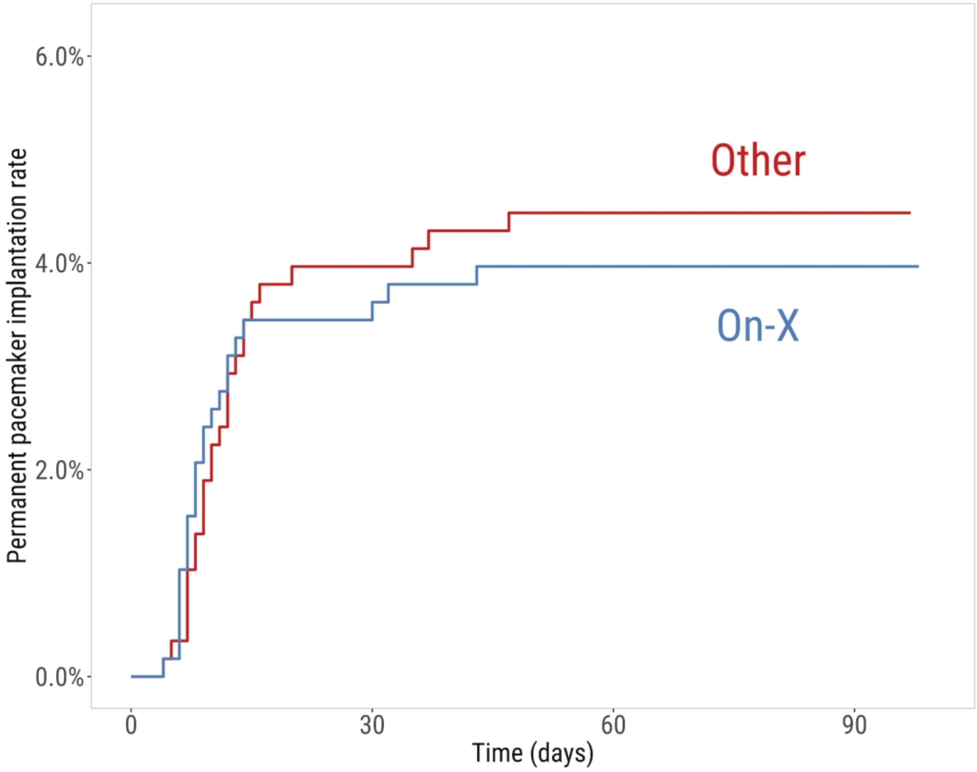

Figure 1. The graph shows the Kaplan-Meier estimated permanent pacemaker implantation rate in propensity score matched patients who received an On-X valve compared to other valves. There was no significant difference in the pacemaker implant rate between the groups. The majority of permanent pacemaker implantations occurred during the first 30 days. No pacemakers were implanted from day 60 to day 120.

150x117mm (300 x 300 DPI)

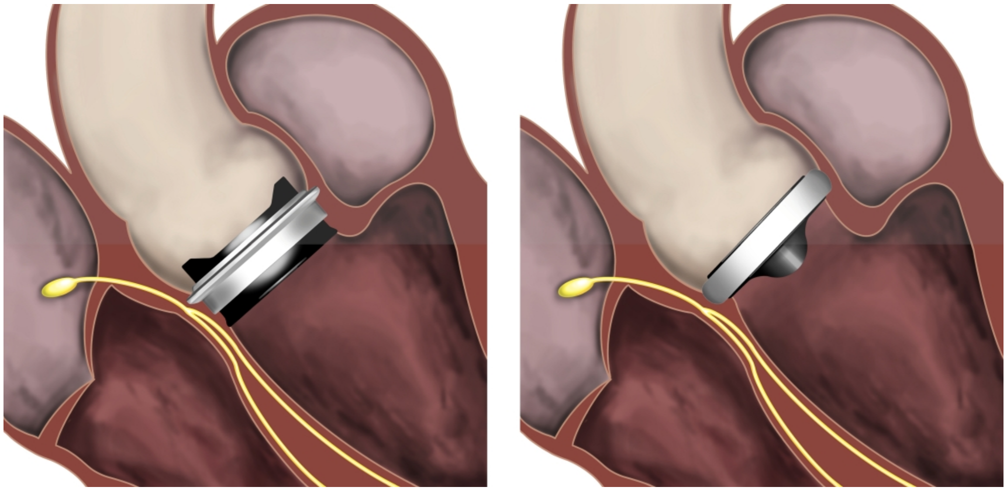

Figure 2. The On-X prosthetic heart valve (On-X Life Technologies, Austin, Texas, USA; left panel) has an elongated subannular valve housing with a flared inlet that protrudes subannulary into the left ventricular outflow tract, in close proximity to the cardiac conduction system. This design distinguishes the On-X prosthetic valve from other contemporary bileaflet mechanical aortic valves, such as the St Jude Regent (St Jude Medical, St Paul, Minnesota, USA; right panel), that are implanted intra-/supraannulary and do not extend subannulary. Illustration: Magnus Dalén.

499x241mm (300 x 300 DPI)

SUPPLEMENTAL MATERIAL

Permanent pacemaker implantation after On-X aortic valve replacement: SWEDEHEART observational cohort study

Magnus Dalén, MD, PhD,[1,2] Michael Persson, MD,[1,2] Natalie Glaser, MD, PhD,[2,3] Ulrik Sartipy, MD, PhD[1,2]

[1]Department of Cardiothoracic Surgery, Karolinska University Hospital, Stockholm, Sweden

[2]Department of Molecular Medicine and Surgery, Karolinska Institutet, Stockholm, Sweden

[3]Department of Cardiology, Stockholm South General Hospital, Stockholm, Sweden

For peer review only

Contents

Supplemental Table 1. Number of patients by year of surgery. 3
Supplemental Table 2. Number of patients according to hospital. 4
Supplemental Table 3. Number of pacemaker implants by year of surgery. 5
Supplemental Table 4. Number of pacemaker implants according to hospital. 6
Supplemental Table 5. Baseline characteristics after IPTW. 7
Supplemental Table 6. Baseline characteristics after overlap weighting. 10
Supplemental Table 7. Height, weight and body surface area. 13
Supplemental Figure 1. Proportion of On-X valves by year of surgery. 14
Supplemental Figure 2. Proportion of On-X valves according to hospital. 15
Supplemental Figure 3. Histogram of propensity scores before and after matching. 16
Supplemental Figure 4. Absolute standardized differences before (hollow circles) and after (filled circles) propensity score matching. 17
Supplemental Figure 5. Proportion of permanent pacemaker implants by year of surgery. 18
Supplemental Figure 6. Proportion of permanent pacemaker implants according to hospital. 19
Supplemental Figure 7. Absolute standardized differences before (hollow circles) and after (filled circles) inverse probability of treatment weighting. 20
Supplemental Figure 8. Absolute standardized differences before (hollow circles) and after (filled circles) overlap weighting. 21
Supplemental Figure 9. Kaplan-Meier estimated pacemaker implantation rate in the inverse probability of treatment weighted population. 22
Supplemental Figure 10. Kaplan-Meier estimated pacemaker implantation rate in the overlap weighted population. 23
Supplemental Figure 11. Crude and adjusted pacemaker implantation rate after On-X valve aortic valve replacement according to alternative approaches for confounding adjustment. 24

For peer review only

Supplemental Table 1. Number of patients by year of surgery.

Year of surgery	Total study population *n = 2602*	On-X valve No *n = 2021*	On-X valve Yes *n = 581*
2005	251 (100%)	244 (97%)	7 (2.8%)
2006	237 (100%)	230 (97%)	7 (3.0%)
2007	215 (100%)	187 (87%)	28 (13%)
2008	214 (100%)	176 (82%)	38 (18%)
2009	152 (100%)	110 (72%)	42 (28%)
2010	166 (100%)	120 (72%)	46 (28%)
2011	133 (100%)	100 (75%)	33 (25%)
2012	145 (100%)	110 (76%)	35 (24%)
2013	157 (100%)	120 (76%)	37 (24%)
2014	158 (100%)	112 (71%)	46 (29%)
2015	141 (100%)	100 (71%)	41 (29%)
2016	221 (100%)	148 (67%)	73 (33%)
2017	193 (100%)	121 (63%)	72 (37%)
2018	219 (100%)	143 (65%)	76 (35%)

Numbers are n (%).

For peer review only

Supplemental Table 2. Number of patients according to hospital.

Hospital	Total study population	On-X valve	
	n = 2602	No *n = 2021*	Yes *n = 581*
1	467 (100%)	413 (88%)	54 (12%)
2	340 (100%)	227 (67%)	113 (33%)
3	329 (100%)	304 (92%)	25 (7.6%)
4	120 (100%)	120 (100%)	0 (0%)
5	398 (100%)	398 (100%)	0 (0%)
6	331 (100%)	243 (73%)	88 (27%)
7	221 (100%)	215 (97%)	6 (2.7%)
8	396 (100%)	101 (26%)	295 (74%)

Numbers are n (%).

For peer review only

Supplemental Table 3. Number of pacemaker implants by year of surgery.

Year of surgery	Total study population *n = 2602*	Pacemaker within 30 days	
		No *n = 2487*	Yes *n = 115*
2005	251 (100%)	243 (97%)	8 (3.2%)
2006	237 (100%)	226 (95%)	11 (4.6%)
2007	215 (100%)	209 (97%)	6 (2.8%)
2008	214 (100%)	207 (97%)	7 (3.3%)
2009	152 (100%)	144 (95%)	8 (5.3%)
2010	166 (100%)	157 (95%)	9 (5.4%)
2011	133 (100%)	130 (98%)	3 (2.3%)
2012	145 (100%)	139 (96%)	6 (4.1%)
2013	157 (100%)	151 (96%)	6 (3.8%)
2014	158 (100%)	153 (97%)	5 (3.2%)
2015	141 (100%)	135 (96%)	6 (4.3%)
2016	221 (100%)	207 (94%)	14 (6.3%)
2017	193 (100%)	182 (94%)	11 (5.7%)
2018	219 (100%)	204 (93%)	15 (6.8%)

Numbers are n (%).

For peer review only

Supplemental Table 4. Number of pacemaker implants according to hospital.

Hospital	Total study population<br>*n = 2602*	Pacemaker within 30 days<br>No<br>*n = 2487*	Pacemaker within 30 days<br>Yes<br>*n = 115*
1	467 (100%)	442 (95%)	25 (5.4%)
2	340 (100%)	316 (93%)	24 (7.1%)
3	329 (100%)	311 (95%)	18 (5.5%)
4	120 (100%)	115 (96%)	5 (4.2%)
5	398 (100%)	389 (98%)	9 (2.3%)
6	331 (100%)	319 (96%)	12 (3.6%)
7	221 (100%)	213 (96%)	8 (3.6%)
8	396 (100%)	382 (96%)	14 (3.5%)

Numbers are n (%).

For peer review only

Supplemental Table 5. Baseline characteristics after IPTW.
Baseline characteristics in patients who underwent mechanical aortic valve replacement with an On-X valve or other mechanical valves in Sweden from 2005 to 2018 before and after inverse probability of treatment weighting.

	Unweighted			IPTW*		
	Control	On-X	SMD	Control	On-X	SMD
Number of patients	2021	581		2573.7	2204.4	
Age (years), mean (SD)	54.1 (9.0)	51.0 (10.7)	0.322	53.5 (9.4)	52.8 (9.6)	0.067
Female sex	525 (26.0)	106 (18.2)	0.187	631.4 (24.5)	517.8 (23.5)	0.024
Non-Nordic birth region	207 (10.2)	32 (5.5)	0.176	238.7 (9.3)	148.1 (6.7)	0.094
Educational level			0.151			0.039
<10 years	588 (29.3)	132 (22.8)		716.6 (28.0)	579.0 (26.4)	
10-12 years	945 (47.1)	303 (52.2)		1230.2 (48.1)	1091.8 (49.8)	
>12 years	474 (23.6)	145 (25.0)		611.6 (23.9)	520.9 (23.8)	
Disposable household income (quartiles)			0.119			0.024
Q1 (low)	520 (25.7)	131 (22.5)		642.7 (25.0)	557.5 (25.3)	
Q2	506 (25.0)	145 (25.0)		648.0 (25.2)	547.9 (24.9)	
Q3	512 (25.3)	138 (23.8)		645.7 (25.1)	535.6 (24.3)	
Q4 (high)	483 (23.9)	167 (28.7)		637.3 (24.8)	563.5 (25.6)	
Married	1065 (52.7)	261 (44.9)	0.156	1318.3 (51.2)	1058.1 (48.0)	0.065
Body mass index (tertiles)			0.012			0.042
T1 (low)	623 (33.5)	185 (33.0)		804.0 (33.6)	743.2 (35.5)	

For peer review only

T2	618 (33.2)	189 (33.7)		798.0 (33.3)	685.6 (32.8)	
T3 (high)	620 (33.3)	187 (33.3)		791.3 (33.1)	663.3 (31.7)	
Atrial fibrillation	183 (9.1)	50 (8.6)	0.016	227.8 (8.9)	210.8 (9.6)	0.025
Heart failure	310 (15.3)	72 (12.4)	0.085	379.4 (14.7)	309.3 (14.0)	0.020
Left ventricular ejection fraction (%)			0.079			0.035
>50	1518 (75.1)	435 (74.9)		1935.0 (75.2)	1679.7 (76.2)	
30-50	389 (19.2)	122 (21.0)		499.8 (19.4)	422.0 (19.1)	
<30	114 (5.6)	24 (4.1)		138.8 (5.4)	102.7 (4.7)	
Chronic obstructive pulmonary disease	136 (6.7)	19 (3.3)	0.159	155.5 (6.0)	96.5 (4.4)	0.075
Diabetes	301 (14.9)	60 (10.3)	0.138	360.6 (14.0)	254.6 (11.5)	0.074
eGFR (ml/min/1,73 m^2)			0.154			0.050
>60	1865 (93.6)	554 (96.9)		2392.7 (94.3)	2066.5 (95.0)	
45-59	85 (4.3)	12 (2.1)		97.5 (3.8)	78.0 (3.6)	
30-44	24 (1.2)	4 (0.7)		27.1 (1.1)	22.7 (1.0)	
<30	18 (0.9)	2 (0.3)		20.3 (0.8)	9.2 (0.4)	
Preoperative dialysis	19 (1.0)	2 (0.4)	0.077	21.5 (0.9)	9.5 (0.5)	0.052
Prior myocardial infarction	193 (9.5)	42 (7.2)	0.084	235.7 (9.2)	173.2 (7.9)	0.047
Prior percutaneous coronary intervention	102 (5.0)	25 (4.3)	0.035	123.8 (4.8)	116.7 (5.3)	0.022
Peripheral vascular disease	208 (10.3)	97 (16.7)	0.188	297.4 (11.6)	294.6 (13.4)	0.055
Hypertension	703 (34.8)	217 (37.3)	0.053	905.7 (35.2)	789.8 (35.8)	0.013
Hyperlipidemia	344 (17.0)	89 (15.3)	0.046	431.7 (16.8)	317.2 (14.4)	0.066
Prior stroke	105 (5.2)	31 (5.3)	0.006	139.3 (5.4)	119.6 (5.4)	0.001

For peer review only

History of cancer	98 (4.8)	30 (5.2)	0.014	126.0 (4.9)	116.2 (5.3)	0.017
Alcohol dependence	69 (3.4)	15 (2.6)	0.049	84.6 (3.3)	50.8 (2.3)	0.059
Liver disease	18 (0.9)	3 (0.5)	0.045	20.9 (0.8)	9.5 (0.4)	0.049
Prior bleeding event	67 (3.3)	15 (2.6)	0.043	81.4 (3.2)	72.6 (3.3)	0.008
Prior endocarditis	92 (4.6)	26 (4.5)	0.004	118.3 (4.6)	116.2 (5.3)	0.031
Emergent operation	32 (1.6)	11 (1.9)	0.024	41.5 (1.6)	36.9 (1.7)	0.005
Coronary artery bypass grafting	363 (18.0)	79 (13.6)	0.120	438.9 (17.1)	296.2 (13.4)	0.101
Valve size (mm)			0.254			0.075
19-21	450 (22.7)	149 (25.8)		589.5 (23.2)	548.5 (25.3)	
23	704 (35.4)	256 (44.3)		944.9 (37.3)	841.0 (38.8)	
25-27	832 (41.9)	173 (29.9)		1001.4 (39.5)	780.5 (36.0)	

Numbers are n (%) unless otherwise specified.
eGFR = estimated glomerular filtration rate, SD = standard deviation, SMD = standardized mean difference
* The overall numbers of patients in each group are not necessarily integers owing to inverse probability of treatment weighting.

For peer review only

Supplemental Table 6. Baseline characteristics after overlap weighting.
Baseline characteristics in patients who underwent mechanical aortic valve replacement with an On-X valve or other mechanical valves in Sweden from 2005 to 2018 before and after overlap weighting.

	Unweighted			Overlap weighting		
	Control	On-X	SMD	Control	On-X	SMD
Number of patients	2021	581		407	407	
Age (years), mean (SD)	54.15 (9.01)	50.97 (10.70)	0.322	51.8 (10.3)	51.8 (10.2)	<0.001
Female sex	525 (26.0)	106 (18.2)	0.187	83 (20.4)	83 (20.4)	<0.001
Non-Nordic birth region	207 (10.2)	32 (5.5)	0.176	25 (6.2)	25 (6.2)	<0.001
Educational level			0.147			<0.001
<10 years	588 (29.1)	132 (22.7)		97 (23.8)	97 (23.8)	
10-12 years	959 (47.5)	304 (52.3)		210 (51.6)	210 (51.6)	
>12 years	474 (23.5)	145 (25.0)		100 (24.6)	100 (24.6)	
Disposable household income (quartiles)			0.119			<0.001
Q1 (low)	520 (25.7)	131 (22.5)		95 (23.4)	95 (23.4)	
Q2	506 (25.0)	145 (25.0)		102 (25.1)	102 (25.1)	
Q3	512 (25.3)	138 (23.8)		98 (24.0)	98 (24.0)	
Q4 (high)	483 (23.9)	167 (28.7)		112 (27.5)	112 (27.5)	
Married	1065 (52.7)	261 (44.9)	0.156	190 (46.7)	190 (46.7)	<0.001
Body mass index (tertiles)			0.053			<0.001
T1 (low)	623 (30.8)	185 (31.8)		131 (32.1)	131 (32.1)	

For peer review only

T2	778 (38.5)	209 (36.0)		149 (36.7)	149 (36.7)	
T3 (high)	620 (30.7)	187 (32.2)		127 (31.2)	127 (31.2)	
Atrial fibrillation	183 (9.1)	50 (8.6)	0.016	35 (8.5)	35 (8.5)	<0.001
Heart failure	310 (15.3)	72 (12.4)	0.085	52 (12.9)	52.4 (12.9)	<0.001
Left ventricular ejection fraction (%)			0.079			<0.001
>50	1518 (75.1)	435 (74.9)		307 (75.4)	307 (75.4)	
30-50	389 (19.2)	122 (21.0)		83 (20.3)	83 (20.3)	
<30	114 (5.6)	24 (4.1)		17 (4.3)	17 (4.3)	
Chronic obstructive pulmonary disease	136 (6.7)	19 (3.3)	0.159	16 (3.8)	16 (3.8)	<0.001
Diabetes	301 (14.9)	60 (10.3)	0.138	45 (11.0)	45 (11.0)	<0.001
eGFR (ml/min/1,73 m^2)			0.153			<0.001
>60	1894 (93.7)	563 (96.9)		392 (96.4)	392 (96.4)	
45-59	85 (4.2)	12 (2.1)		10 (2.4)	10 (2.4)	
30-44	24 (1.2)	4 (0.7)		3 (0.8)	3 (0.8)	
<30	18 (0.9)	2 (0.3)		2 (0.4)	2 (0.4)	
Preoperative dialysis	19 (0.9)	2 (0.3)	0.075	2 (0.4)	2 (0.4)	<0.001
Prior myocardial infarction	193 (9.5)	42 (7.2)	0.084	32 (7.9)	32 (7.9)	<0.001
Prior percutaneous coronary intervention	102 (5.0)	25 (4.3)	0.035	19 (4.6)	19 (4.6)	<0.001
Peripheral vascular disease	208 (10.3)	97 (16.7)	0.188	60 (14.7)	60 (14.7)	<0.001
Hypertension	703 (34.8)	217 (37.3)	0.053	149 (36.6)	149 (36.6)	<0.001
Hyperlipidemia	344 (17.0)	89 (15.3)	0.046	63 (15.6)	63 (15.6)	<0.001
Prior stroke	105 (5.2)	31 (5.3)	0.006	22 (5.4)	22 (5.4)	<0.001

For peer review only

History of cancer	98 (4.8)	30 (5.2)	0.014	22 (5.3)	22 (5.3)	<0.001
Alcohol dependence	69 (3.4)	15 (2.6)	0.049	11 (2.7)	11 (2.7)	<0.001
Liver disease	18 (0.9)	3 (0.5)	0.045	2 (0.6)	2 (0.6)	<0.001
Prior bleeding event	67 (3.3)	15 (2.6)	0.043	11 (2.7)	11 (2.7)	<0.001
Prior endocarditis	92 (4.6)	26 (4.5)	0.004	18 (4.4)	18 (4.4)	<0.001
Emergent operation	32 (1.6)	11 (1.9)	0.024	7 (1.7)	7 (1.7)	<0.001
Coronary artery bypass grafting	363 (18.0)	79 (13.6)	0.120	59 (14.5)	59 (14.5)	<0.001
Valve size (mm)			0.390			<0.001
19	74 (3.7)	32 (5.5)		21 (5.2)	21 (5.2)	
21	376 (18.6)	117 (20.1)		83 (20.4)	83 (20.4)	
23	739 (36.6)	259 (44.6)		176 (43.2)	176 (43.2)	
25	584 (28.9)	157 (27.0)		113 (27.7)	113 (27.7)	
27	248 (12.3)	16 (2.8)		14 (3.5)	14 (3.5)	

Numbers are n (%) unless otherwise specified.
eGFR = estimated glomerular filtration rate, SD = standard deviation, SMD = standardized mean difference

For peer review only

Supplemental Table 7. Height, weight and body surface area.

	Before matching			
	Overall	Control	On-X	SMD
Number of patients	2602	2021	581	
Height, cm	175 (9.4)	175 (9.5)	177 (8.8)	0.216
Weight, kg	86.0 (17.3)	85.5 (17.1)	87.5 (17.8)	0.116
BSA, m^2	2.04 (0.23)	2.03 (0.23)	2.07 (0.23)	0.154

Numbers are mean and standard deviation.
BSA = body surface area, SMD = standardized mean difference

For peer review only

Supplemental Figure 1. Proportion of On-X valves by year of surgery.

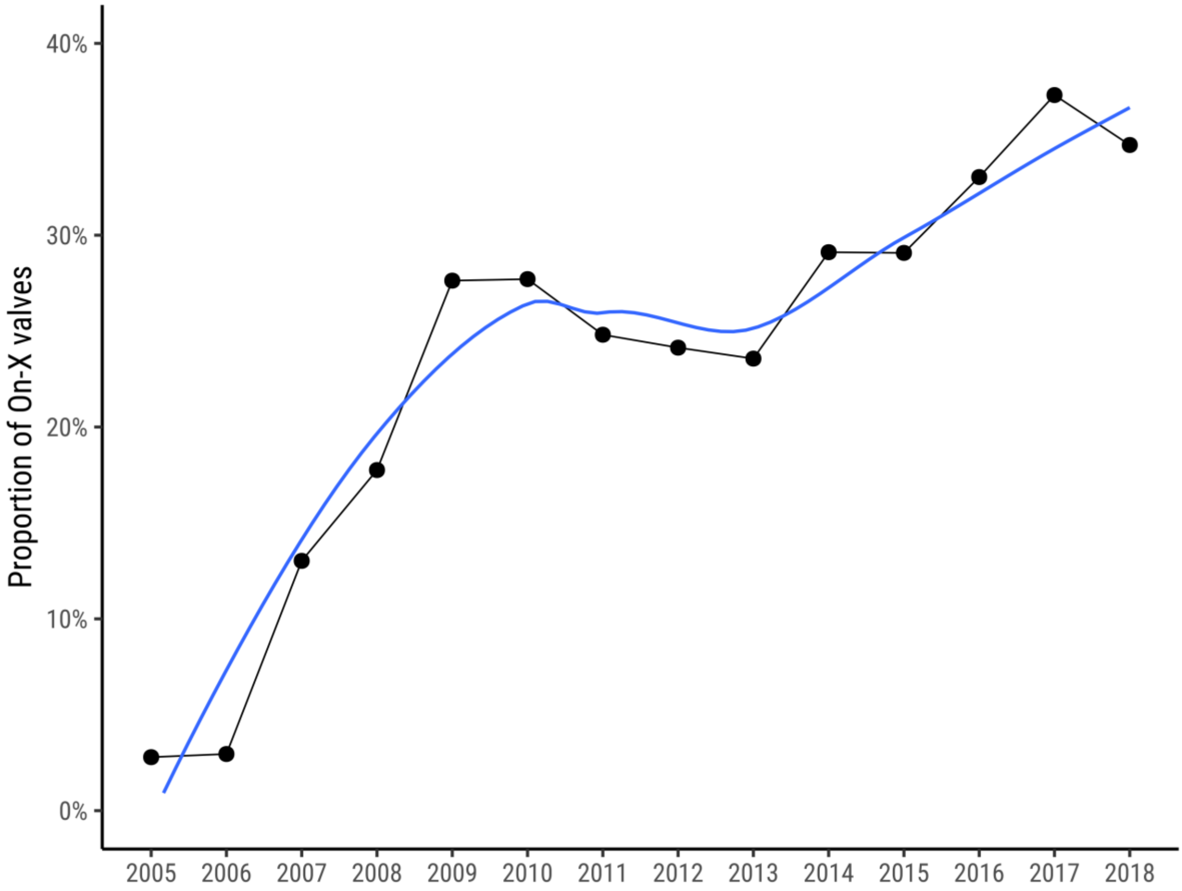

w only

Supplemental Figure 2. Proportion of On-X valves according to hospital.

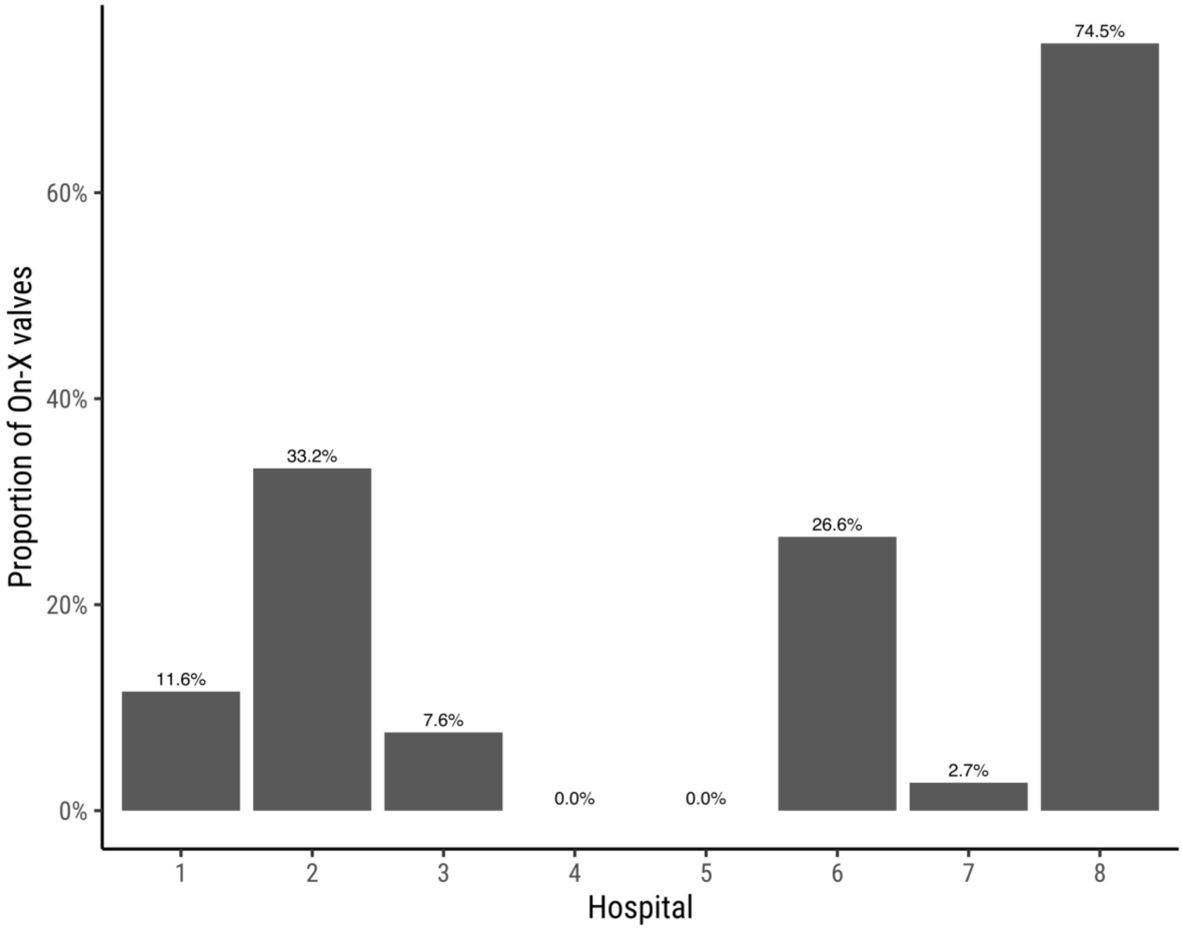

w only

Supplemental Figure 3. Histogram of propensity scores before and after matching.

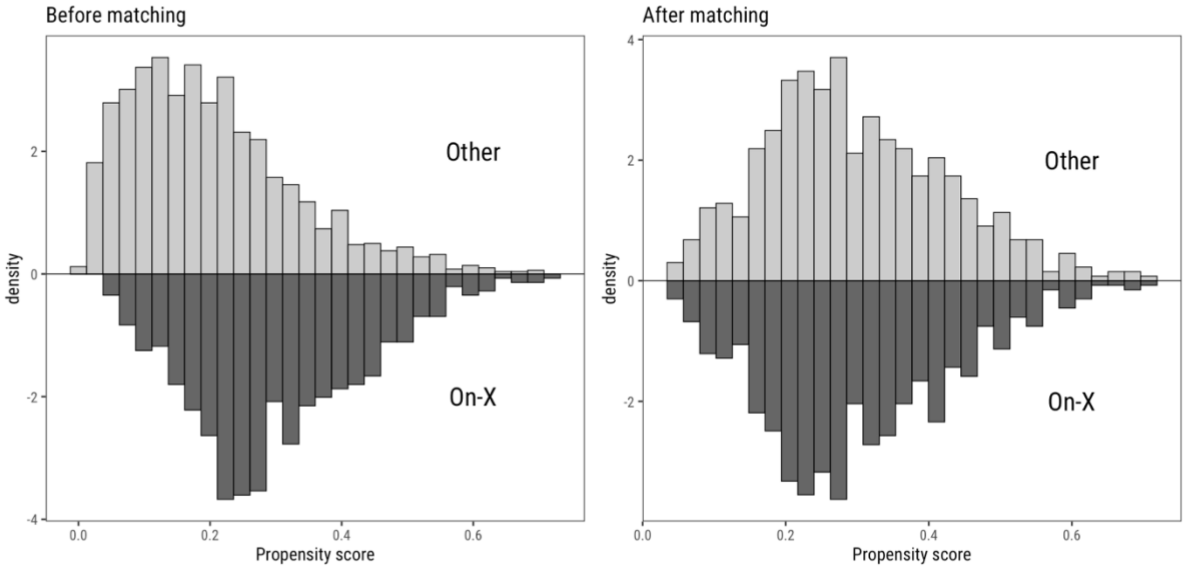

eer review only

Supplemental Figure 4. Absolute standardized differences before (hollow circles) and after (filled circles) propensity score matching.

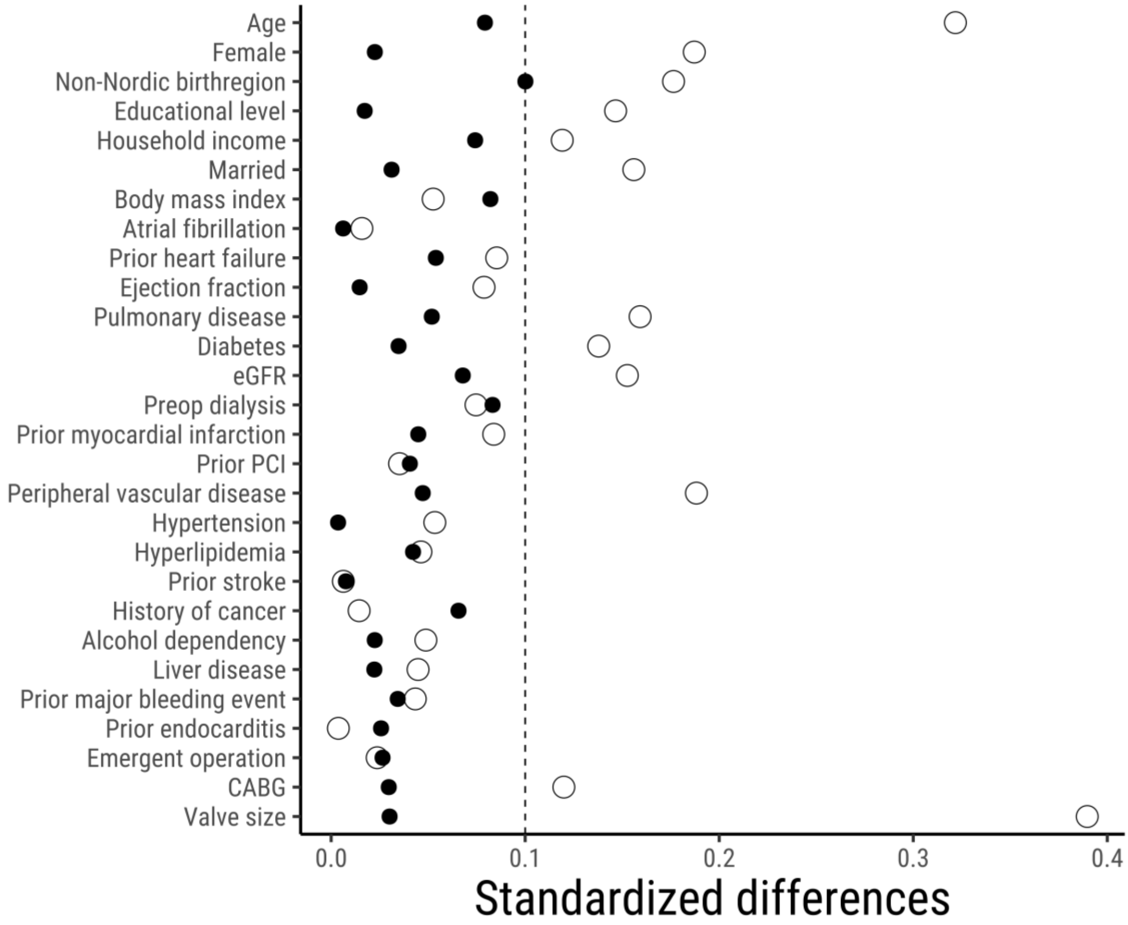

For peer review only - http://bmjopen.bmj.com/site/about/guidelines.xhtml

Supplemental Figure 5. Proportion of permanent pacemaker implants by year of surgery.

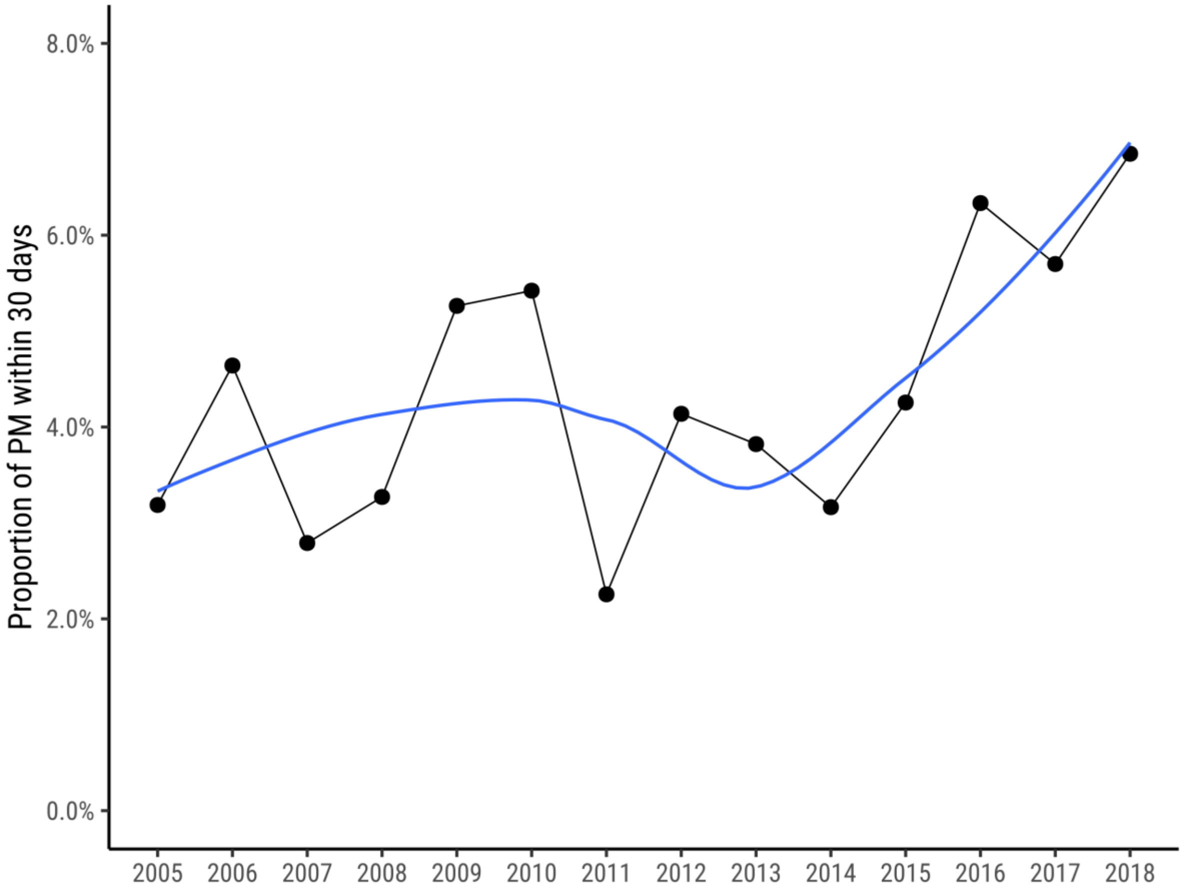

w only

Supplemental Figure 6. Proportion of permanent pacemaker implants according to hospital.

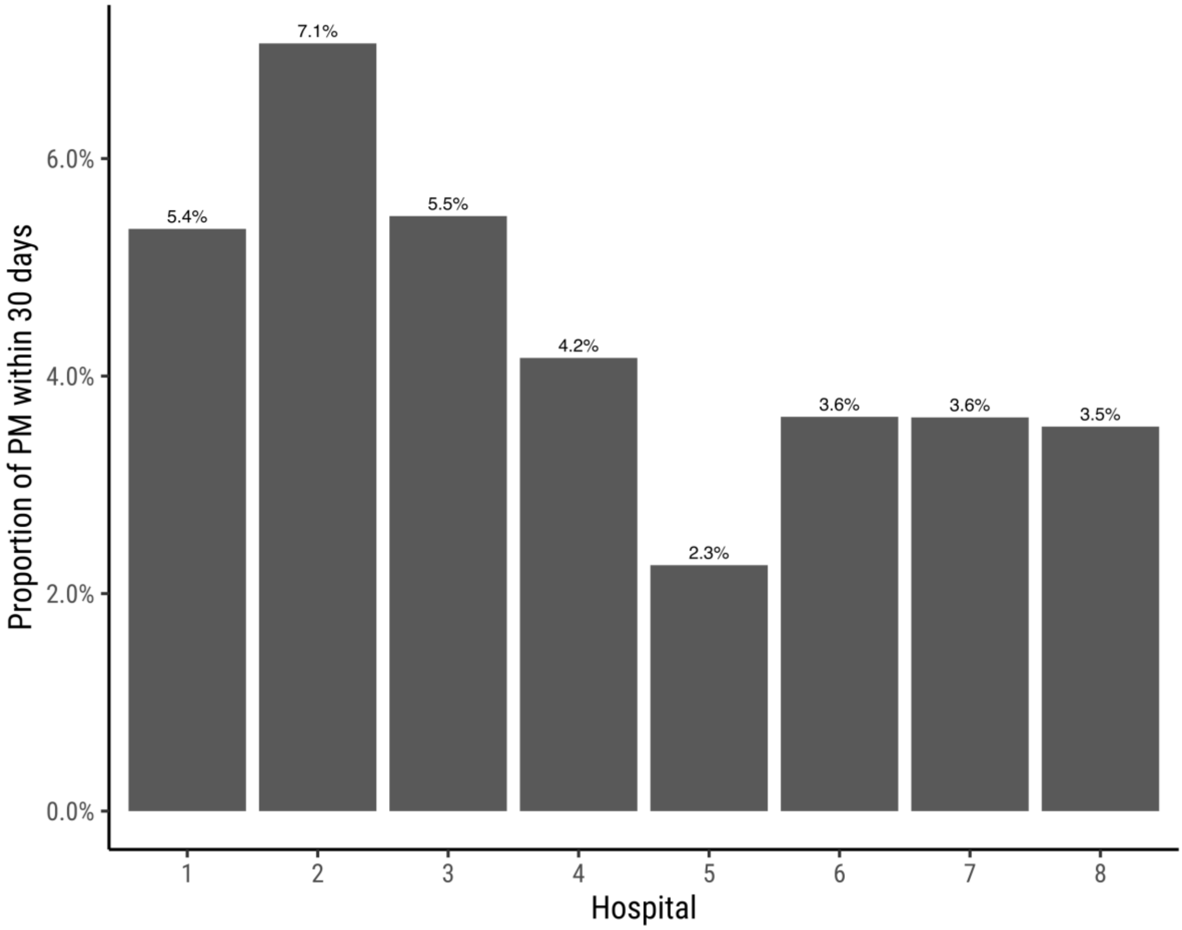

w only

Supplemental Figure 7. Absolute standardized differences before (hollow circles) and after (filled circles) inverse probability of treatment weighting.

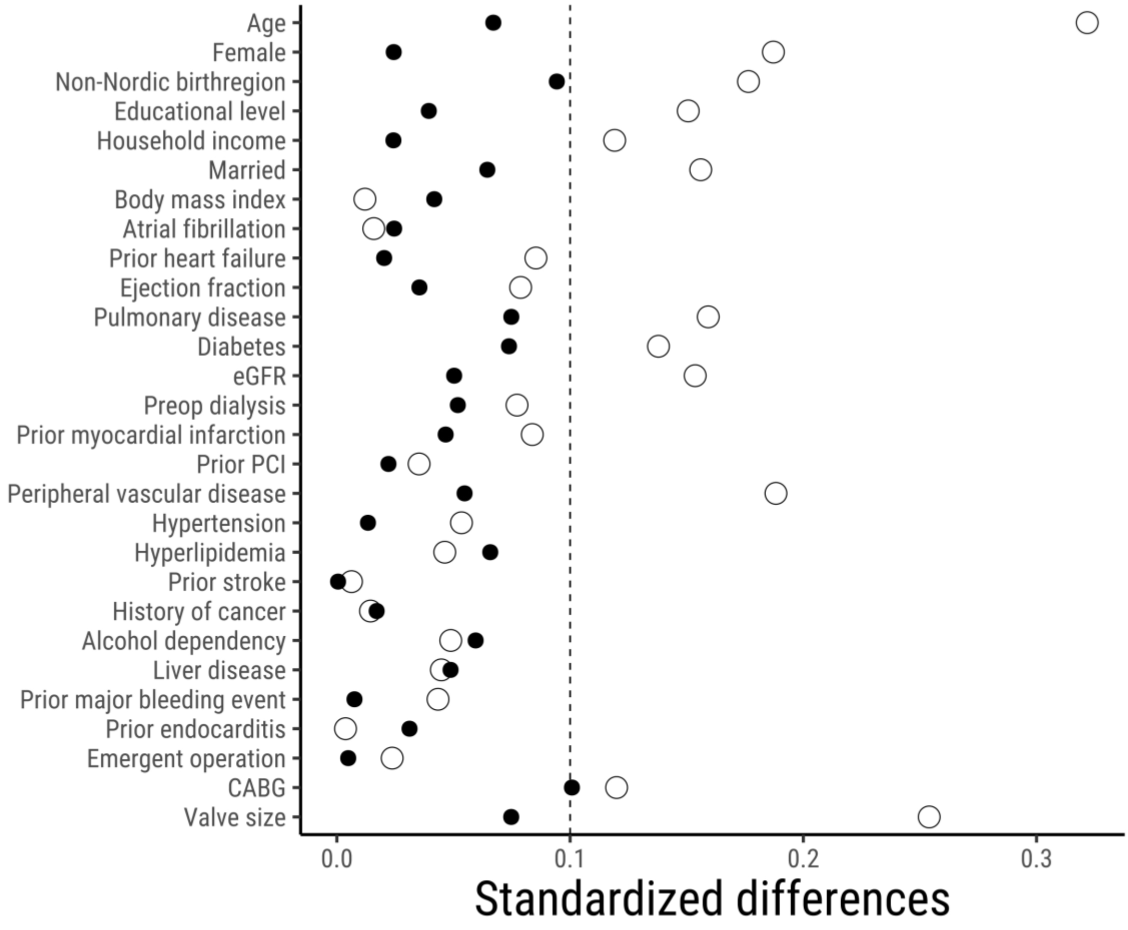

Supplemental Figure 8. Absolute standardized differences before (hollow circles) and after (filled circles) overlap weighting.

Note: This method yields exact covariate balance between treated and reference groups by construction.

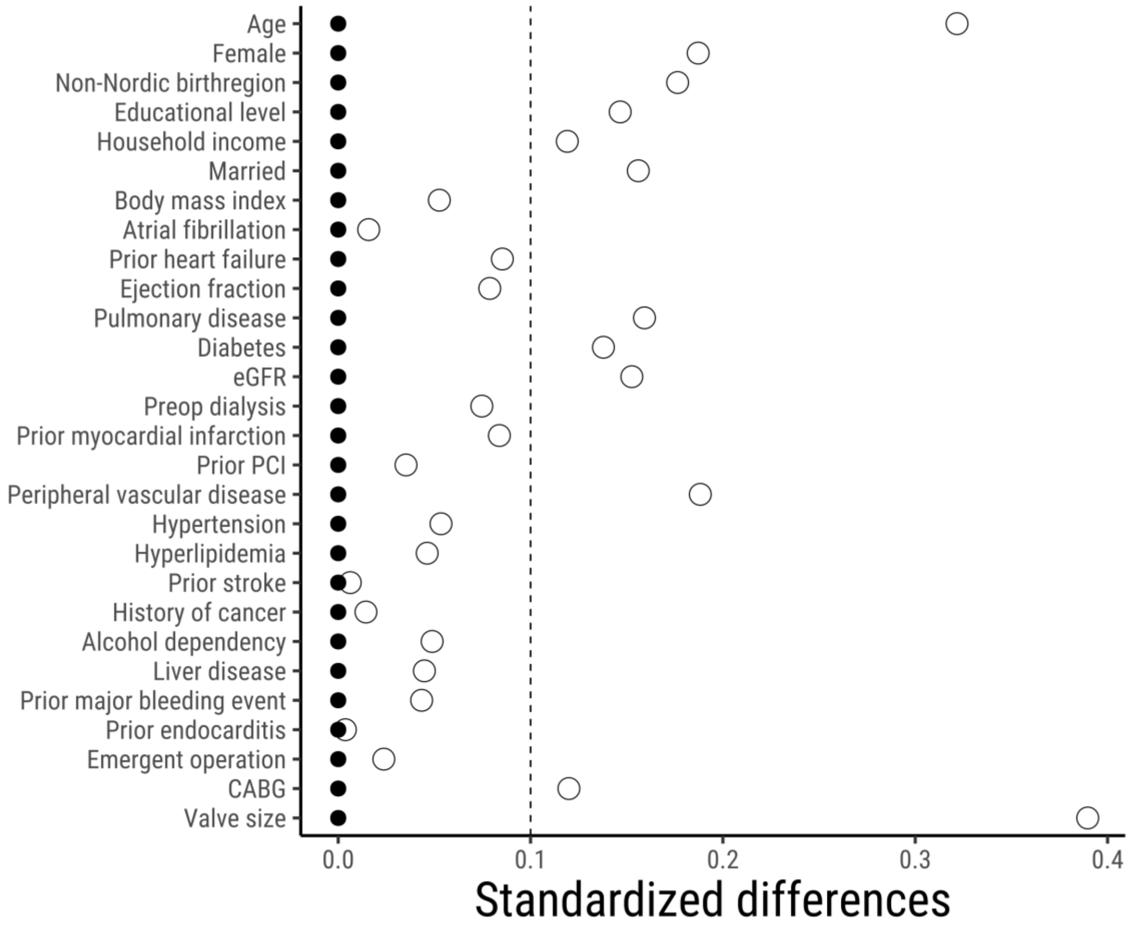

Supplemental Figure 9. Kaplan-Meier estimated pacemaker implantation rate in the inverse probability of treatment weighted population.

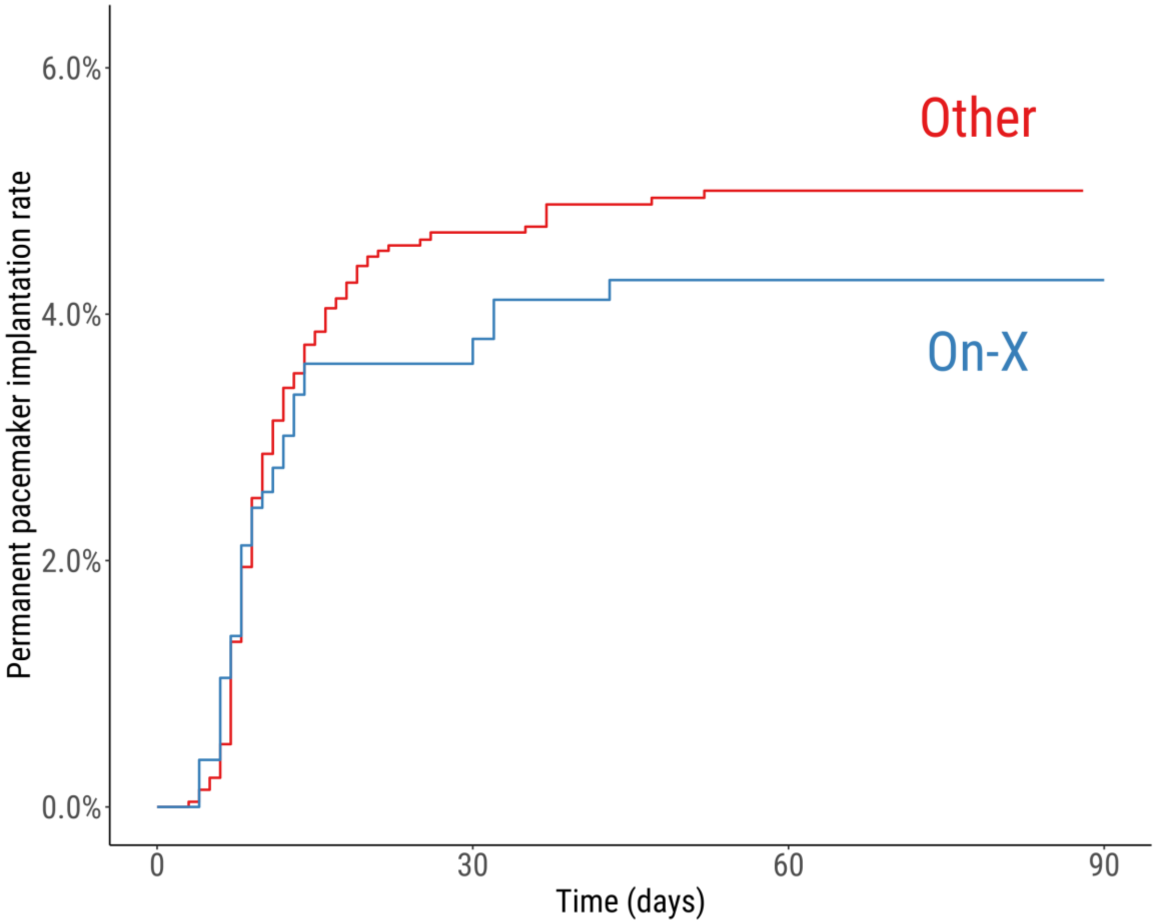

Supplemental Figure 10. Kaplan-Meier estimated pacemaker implantation rate in the overlap weighted population.

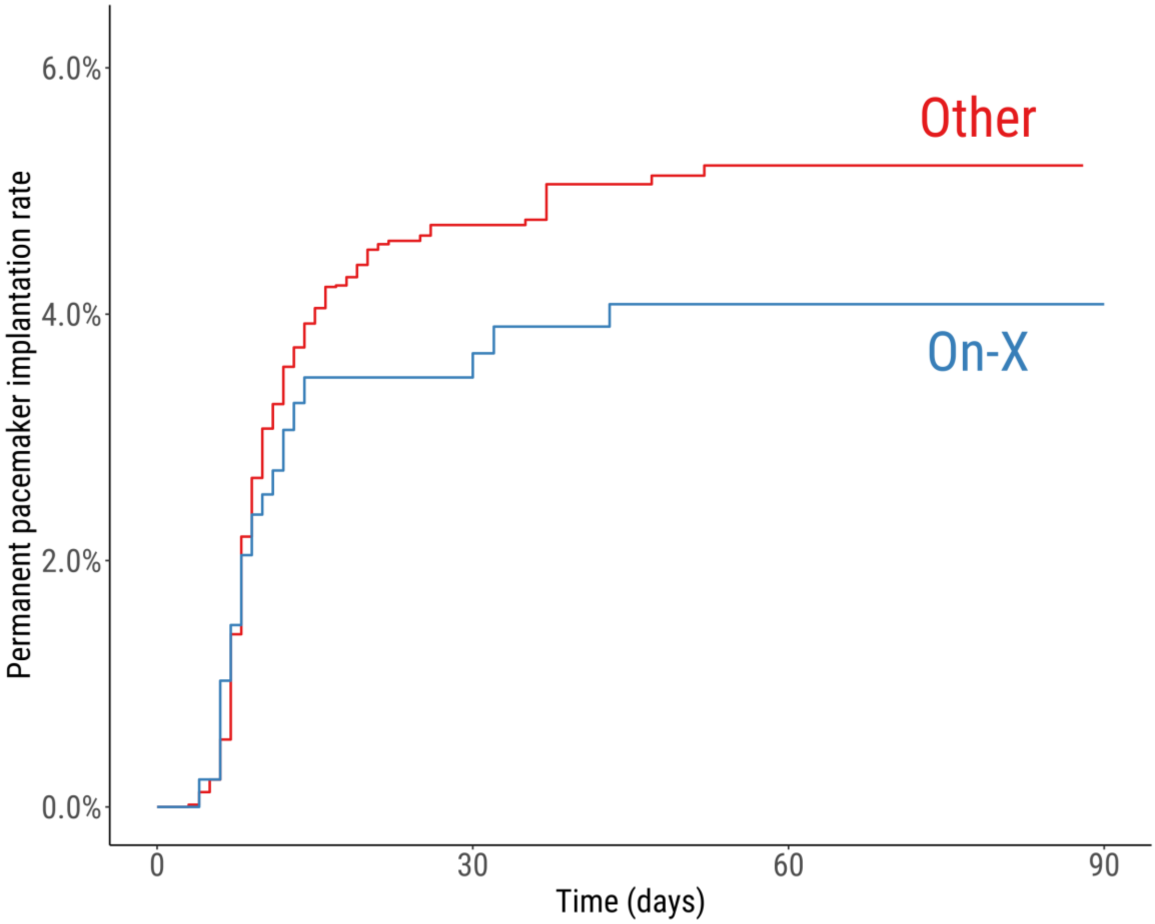

Supplemental Figure 11. Crude and adjusted pacemaker implantation rate after On-X valve aortic valve replacement according to alternative approaches for confounding adjustment.

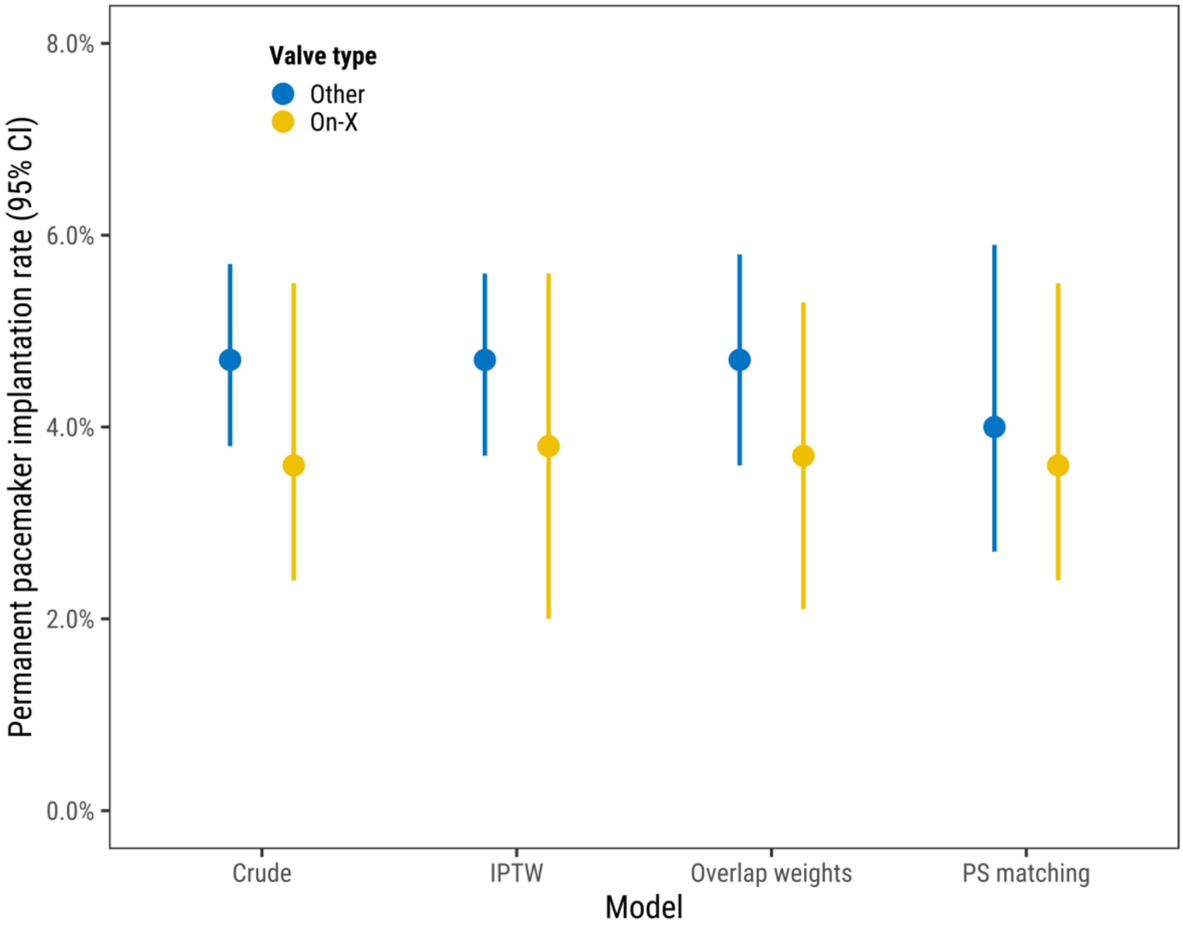

Note: IPTW = inverse probability of treatment weighting, PS = propensity score

Reporting checklist for cohort study.

Based on the STROBE cohort guidelines.

Instructions to authors

Complete this checklist by entering the page numbers from your manuscript where readers will find each of the items listed below.

Your article may not currently address all the items on the checklist. Please modify your text to include the missing information. If you are certain that an item does not apply, please write "n/a" and provide a short explanation.

Upload your completed checklist as an extra file when you submit to a journal.

In your methods section, say that you used the STROBE cohortreporting guidelines, and cite them as:

von Elm E, Altman DG, Egger M, Pocock SJ, Gotzsche PC, Vandenbroucke JP. The Strengthening the Reporting of Observational Studies in Epidemiology (STROBE) Statement: guidelines for reporting observational studies.

		Reporting Item	Page Number
**Title and abstract**			
Title	#1a	Indicate the study's design with a commonly used term in the title or the abstract	1
Abstract	#1b	Provide in the abstract an informative and balanced summary of what was done and what was found	2
**Introduction**			
Background / rationale	#2	Explain the scientific background and rationale for the investigation being reported	4
Objectives	#3	State specific objectives, including any prespecified hypotheses	5
**Methods**			
Study design	#4	Present key elements of study design early in the paper	5
Setting	#5	Describe the setting, locations, and relevant dates, including periods	5

For peer review only

		of recruitment, exposure, follow-up, and data collection	
Eligibility criteria	#6a	Give the eligibility criteria, and the sources and methods of selection of participants. Describe methods of follow-up.	5
Eligibility criteria	#6b	For matched studies, give matching criteria and number of exposed and unexposed	5
Variables	#7	Clearly define all outcomes, exposures, predictors, potential confounders, and effect modifiers. Give diagnostic criteria, if applicable	6
Data sources / measurement	#8	For each variable of interest give sources of data and details of methods of assessment (measurement). Describe comparability of assessment methods if there is more than one group. Give information separately for for exposed and unexposed groups if applicable.	5
Bias	#9	Describe any efforts to address potential sources of bias	9
Study size	#10	Explain how the study size was arrived at	n/a
Quantitative variables	#11	Explain how quantitative variables were handled in the analyses. If applicable, describe which groupings were chosen, and why	6
Statistical methods	#12a	Describe all statistical methods, including those used to control for confounding	
6			
Statistical methods	#12b	Describe any methods used to examine subgroups and interactions	6
Statistical methods	#12c	Explain how missing data were addressed	7
Statistical methods	#12d	If applicable, explain how loss to follow-up was addressed	7
Statistical methods	#12e	Describe any sensitivity analyses	
n/a			
**Results**			
Participants	#13a	Report numbers of individuals at each stage of study—eg numbers potentially eligible, examined for eligibility, confirmed eligible,	8

For peer review only

		included in the study, completing follow-up, and analysed. Give information separately for for exposed and unexposed groups if applicable.	
Participants	#13b	Give reasons for non-participation at each stage	n/a
Participants	#13c	Consider use of a flow diagram	
n/a			
Descriptive data	#14a	Give characteristics of study participants (eg demographic, clinical, social) and information on exposures and potential confounders. Give information separately for exposed and unexposed groups if applicable.	8
Descriptive data	#14b	Indicate number of participants with missing data for each variable of interest	
7			
Descriptive data	#14c	Summarise follow-up time (eg, average and total amount)	
8			
Outcome data	#15	Report numbers of outcome events or summary measures over time. Give information separately for exposed and unexposed groups if applicable.	
8			
Main results	#16a	Give unadjusted estimates and, if applicable, confounder-adjusted estimates and their precision (eg, 95% confidence interval). Make clear which confounders were adjusted for and why they were included	8
Main results	#16b	Report category boundaries when continuous variables were categorized	8
Main results	#16c	If relevant, consider translating estimates of relative risk into absolute risk for a meaningful time period	
n/a			
Other analyses	#17	Report other analyses done—eg analyses of subgroups and interactions, and sensitivity analyses	9

Discussion

For peer review only

Key results	#18	Summarise key results with reference to study objectives	10
Limitations	#19	Discuss limitations of the study, taking into account sources of potential bias or imprecision. Discuss both direction and magnitude of any potential bias.	11
Interpretation	#20	Give a cautious overall interpretation considering objectives, limitations, multiplicity of analyses, results from similar studies, and other relevant evidence.	11
Generalisability	#21	Discuss the generalisability (external validity) of the study results	11
**Other Information**			
Funding	#22	Give the source of funding and the role of the funders for the present study and, if applicable, for the original study on which the present article is based	13

The STROBE checklist is distributed under the terms of the Creative Commons Attribution License CC-BY. This checklist was completed on 13. December 2020 using https://www.goodreports.org/, a tool made by the EQUATOR Network in collaboration with Penelope.ai

For peer review only
